# Prediction of Gene Expression in Embryonic Structures of Drosophila melanogaster

**DOI:** 10.1371/journal.pcbi.0030144

**Published:** 2007-07-20

**Authors:** Anastasia A Samsonova, Mahesan Niranjan, Steven Russell, Alvis Brazma

**Affiliations:** 1 European Bioinformatics Institute, Wellcome Trust Genome Campus, Cambridge, United Kingdom; 2 Department of Computer Science, University of Sheffield, Sheffield, United Kingdom; 3 Department of Genetics, University of Cambridge, Cambridge, United Kingdom; Duke University, United States of America

## Abstract

Understanding how sets of genes are coordinately regulated in space and time to generate the diversity of cell types that characterise complex metazoans is a major challenge in modern biology. The use of high-throughput approaches, such as large-scale in situ hybridisation and genome-wide expression profiling via DNA microarrays, is beginning to provide insights into the complexities of development. However, in many organisms the collection and annotation of comprehensive in situ localisation data is a difficult and time-consuming task. Here, we present a widely applicable computational approach, integrating developmental time-course microarray data with annotated in situ hybridisation studies, that facilitates the de novo prediction of tissue-specific expression for genes that have no in vivo gene expression localisation data available. Using a classification approach, trained with data from microarray and in situ hybridisation studies of gene expression during *Drosophila* embryonic development, we made a set of predictions on the tissue-specific expression of *Drosophila* genes that have not been systematically characterised by in situ hybridisation experiments. The reliability of our predictions is confirmed by literature-derived annotations in FlyBase, by overrepresentation of Gene Ontology biological process annotations, and, in a selected set, by detailed gene-specific studies from the literature. Our novel organism-independent method will be of considerable utility in enriching the annotation of gene function and expression in complex multicellular organisms.

## Author Summary

The task of deciphering the complex transcriptional regulatory networks controlling development is one of the major current challenges for molecular biology. The problem is difficult, if not impossible, to solve without a detailed knowledge of the spatiotemporal dynamics of gene expression. Thus, to understand development, we need to identify and functionally characterize all players in regulatory networks. Data on gene expression dynamics obtained from whole transcriptome microarray experiments, combined with in situ hybridization mRNA localisation patterns for a subset of genes, may provide a route for predicting the localisation of gene expression for those genes for which in situ data has not been generated, as well as suggesting functional information for uncharacterised genes. Here, we report the development of one of the first methods for predicting the localisation of gene expression during *Drosophila* embryogenesis from microarray data. Pooling the subset of genes in the fly genome with in situ data to form functional units, localised in space and time for relevant developmental processes, facilitates the statement of a classification problem, which we address with machine-learning methods. Our approach promotes a richer annotation of biological function for genes in the absence of costly and time-consuming experimental analysis.

## Introduction

As a result of a gradual developmental strategy known as epigenesis, an embryo comprising a few cell types is refined to generate a complex organism composed of many precisely organized anatomical structures. Understanding how the genome is dynamically deployed to generate such cellular diversity is a key challenge in modern biology. Spatiotemporal information on gene expression can provide insights into the biological function of gene products, since genes belonging to the same developmental pathway tend to have similar or correlated expression patterns [1–3]. Until recently, research efforts aimed at deciphering the spatiotemporal dynamics of gene expression have been primarily carried out on a small scale, since they were predefined by either a specific gene network or a single gene of interest.

Advances in molecular tagging and imaging techniques, along with high-throughput experimental methods, offer new possibilities for following developmental processes by investigating the spatiotemporal dynamics of gene expression and helping to reveal gene function at a whole genome scale. High-throughput experimental approaches, such as DNA microarrays and mRNA in situ hybridization, have been used to probe gene expression during development in several model organisms [4–8], including the fruit fly Drosophila melanogaster [9]. Microarray studies, using mRNA samples extracted at various stages of embryonic development, provide time courses of expression for individual genes, while in situ hybridization with specific gene probes identifies spatial and tissue-specific expression of particular target genes. Of these, analysis of the former is mostly automatic, while the latter requires manual annotation by experts. Whole-organism microarrays provide semiquantitative information on changes in gene expression levels during the course of development; however, it is difficult to infer spatial information about gene expression since the technique generally uses RNA extracted from whole embryos. Integrative analysis of in situ expression patterns and microarray gene expression data is one possible way to assist in deciphering the roles that genes play during development and to identify sets of genes involved in similar developmental processes. Data on the dynamics of gene expression obtained with whole genome microarray experiments combined with in situ expression patterns may thus provide a starting point for the prediction of gene expression localization and assignment of putative function for those genes for which in situ data has not been yet generated.

In this report, we describe the development of a computational method for predicting the spatial localization of gene expression from microarray data. Our method provides a route for elucidating the roles a gene may play during development by inference from the spatial annotation of its expression [9]. Our approach, based on a machine-learning framework, allows de novo prediction of gene expression localization by training a classifier on a subset of genes with spatial expression patterns annotated by in situ hybridisation experiments. While a variety of clustering algorithms are popular for the analysis of microarray data, their ability to infer gene function or expression localisation is limited. An alternative method of meta-analysis, classification, has been widely used in the context of diagnostics; i.e., separating patients with a disease from the normal population, (see, for example, [10]), or identifying conserved modules in genetic networks [11]. The problem statement in our case resembles the work of Wong et al. [12] and Brown et al. [13], where the problems of predicting gene interactions and of inferring gene function from high-throughput data, respectively, have been formulated as classification tasks.

To develop our approach, we focused on a system—the development of the *Drosophila* embryo—that has substantial high-quality in situ hybridization data and comprehensive microarray time-course data available. Starting from the anatomical annotation of in situ gene expression patterns obtained by the Berkeley *Drosophila* Genome Project (BDGP; http://www.fruitfly.org) [9], we assembled genes involved in specific developmental process into groups we define as functional units. We then trained a machine-learning algorithm with microarray data to discriminate between the genes associated with a particular developmental process and the remainder of the genes that are not. The most suitable classifiers, along with a set of functional units producing best prediction results, were selected via a multilevel verification procedure. De novo predictions of tissue-specific expression for genes with only microarray data available were confirmed with literature data and with Gene Ontology (GO):Biological Process annotation. Our method provides a generalized route for generating preliminary functional annotations for genes of unknown function.

## Results/Discussion

### Functional Units

To predict gene expression localization, we selected several developmental events taking place at different time intervals during embryogenesis and assembled genes acting in these processes into functional units. We define a functional unit as a set of genes, known to be involved in a particular biological process during a contiguous developmental time interval, expressed in a predefined group of anatomical structures related by developmental lineage. A gene is assigned to the functional unit if, and only if, it is expressed in all anatomical structures in a particular lineage of interest. Thus, a functional unit not only reflects the spatiotemporal dynamics of the expression of a set of genes involved in the biological process under study, but also suggests that a set of genes making up a unit act concordantly in a specified event in organogenesis (see Materials and Methods).

As an example of this approach, we consider assembling genes into a functional unit that reflects the development of the central nervous system (CNS). Formation of the individual tissues of the CNS from their primordial embryonic structures takes place during late embryogenesis, in the 6-h interval spanning stages 9 to 15 of *Drosophila* development [14]. This is a highly ordered process, which can be presented schematically in three steps [15,16]. The primordium of the CNS is established at stage 9 when neuroblasts, which originate from a precisely defined area of the neurogenic ectoderm, delaminate from the ectoderm into the embryo. Neuroblasts are large cells with stem cell properties whose progeny will populate the differentiated CNS. Neuronal differentiation begins at stage 13, with the formation of neurons and initiation of axonal outgrowth, and shortly after this, at stage 14, the ventral nerve cord condenses [14]. Therefore, functional units corresponding to CNS development are compiled from the genes that are expressed in the ventral neuroderm anlage and the anatomical structures that derive from it: ventral nerve cord primordium and lateral cord. Every gene annotated as being expressed in all three of these anatomical structures in the BDGP in situ database is attributed to the *vna2lcord* functional unit.

We constructed and examined a total of 15 functional units encompassing three developmental processes occurring in late embryogenesis, one process occurring in mid-embryogenesis, and one process occurring during early development (see Table 1 for the units considered and Materials and Methods for the construction schema). The genes in the functional units are involved in the formation of the procephalic ectoderm primordium, the development of the mesoderm, and the precursors of the embryonic muscle system, embryonic CNS, and, finally, the embryonic digestive system. The functional units are labeled by an abbreviation that reflects the morphological changes in the organism during embryogenesis (i.e., the initial developmental intermediate structure and the terminal differentiated anatomical structure used to assemble a functional unit; Table 1). To associate microarray data with these functional units, we divided a time course of gene expression [9] into three alternating time windows corresponding to early, middle, and late embryogenesis.

**Table 1:**
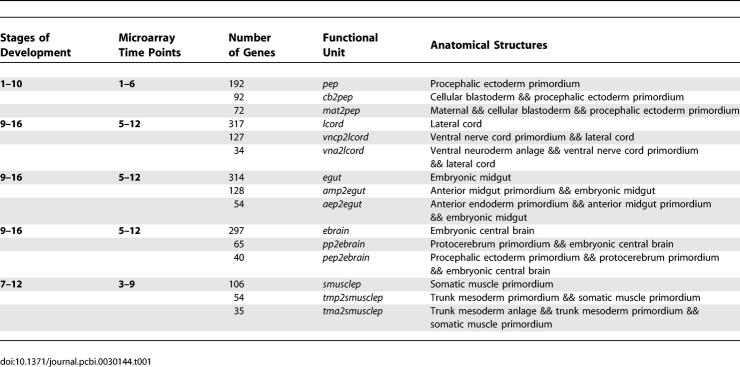
Functional Units Assembled To Predict the Localization of Expression in *Drosophila* Embryogenesis

**Figure 1:**
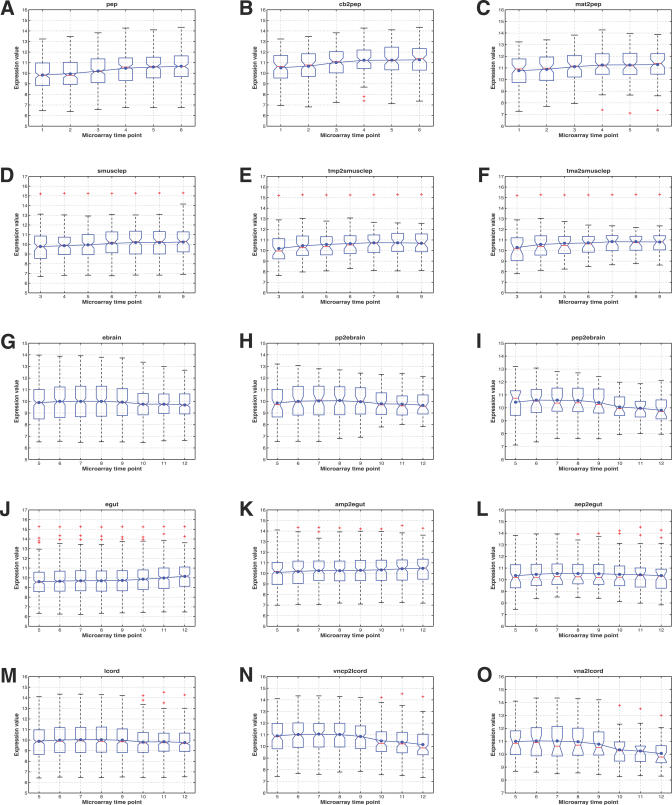
Dynamics of Gene Expression Detected with Microarray Experiment with Respect to Sets of Anatomical Structures Selected to Form Functional Units The boxes have lines at the upper, median, and lower quartile values. Whiskers extend from each end of the box to the adjacent values in the profile data. The most extreme values are within 1.5 times interquartile range from the ends of the box. Outliers (marked with red plus signs) are data beyond the ends of the whiskers. Average microarray profiles are shown in blue continuous lines.

Average microarray profiles constructed for selected functional units (see Figures 1 and S1) show changes in the dynamics of microarray gene expression data attributed to different units. The variability in microarray expression signals decreases as the number of anatomical structures a functional unit is assembled with increases. The expression patterns stored in the BDGP database are very rarely identical; however, there is still a noticeable similarity among expression patterns for many genes [9]. Indeed, the anatomical annotations for genes comprising functional units are not homogeneous and display remarkable diversity. Figures S10 to S14 and Tables S14 to S18 show the variability in annotations of expression patterns for genes attributed to the five most complex functional units. Anatomical annotations for functional units encompassing developmental processes that are spaced several stages apart show very little similarity. In contrast, annotations for the two functional units *(pep2ebrain* and *vna2lcord)* assembled from genes involved in CNS development during late embryogenesis are similar. Furthermore, although genes are rarely attributed to multiple functional units, these two units are exceptional and share many genes (see Figure S15 and Figure S17A and Table S13).

The developmental processes we selected are major events in *Drosophila* embryogenesis, and consequently have been well-characterized by many researchers. This is important since, in order to verify predictions of tissue-specific expression and to estimate the performance of the proposed classification scheme, we needed to choose extensively annotated and studied events during organogenesis. In addition, the choice of these processes was partially predefined by the nature of the in situ dataset available from the BDGP (i.e., by the number of genes involved in each process and by the time span of the developmental processes; see Materials and Methods for details). Furthermore, to demonstrate the flexibility of the method, we selected processes from diverse time intervals during *Drosophila* development, specifically focusing on overlapping developmental processes during late embryogenesis, to determine whether our classification approach is able to separate sets of genes with different tissue-specific annotations but very similar microarray gene expression profiles.

### Classifier Design and Performance

The formulation of our classification scheme uses microarray data as input features and derives class labels as to whether a gene belongs to a functional unit or not (see Figure S2). Because the number of training examples was low, with a few dozen genes in a functional unit being considered, classifier design had to be performed with care to avoid overfitting by classifiers that form complex nonlinear boundaries. We achieved this by extensive cross-validation and bootstrapping to set classifier parameters. Classifiers were evaluated on out-of-sample data (test data) for which BDGP in situ hybridization images exist, and a selected classifier was used in subsequent de novo predictions on genes for which only microarray expression profiles exist. The gene expression profiles utilised for training the classification method were excluded from the test set subsequently used for de novo prediction of gene expression localisation.

**Figure 2:**
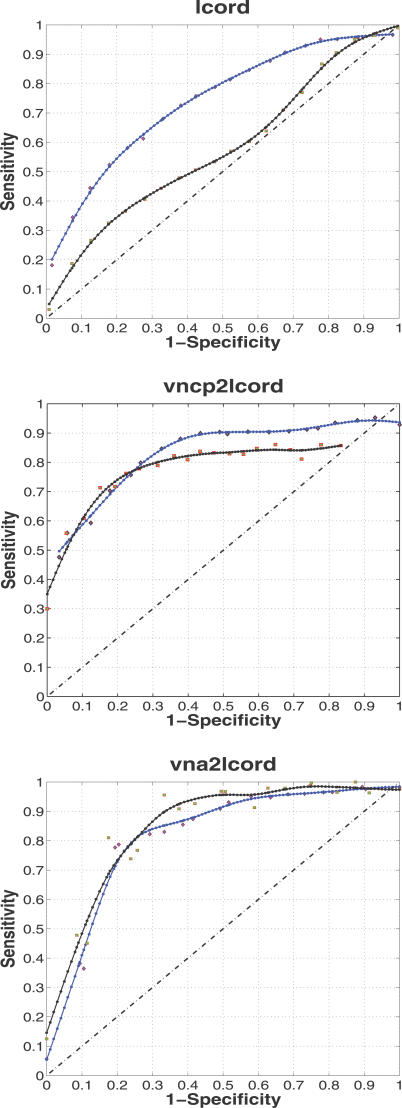
Estimation of Prediction Accuracy for Three Functional Units Constructed with Genes Involved in the Development of Embryonic CNS Discrimination (between the genes in the functional unit and genes that do not belong there) is shown by average ROC curves of SVM classifiers. The black and blue curves (radial basis function [RBF] and second-order polynomial kernels, respectively) are average ROC curves obtained with cubic smoothing spline, which approximate average sensitivity values. The average sensitivity values are green squares (RBF kernel) and magenta diamonds (second-order polynomial kernel). The diagonal line represents a classifier that makes random predictions. The classifier designed with the RBF kernel outperforms the classifier built with the second-order polynomial kernel, reaching peak performance characteristics of 80% sensitivity (true-positive rate) and specificity (1 − false-positive rate). See Tables S2 and S3 and Figure S6 for reference.

The discriminability in the data is higher in functional units with a higher number of anatomical structures in any developmental lineage (i.e., recognition rates for genes in *vna2lcord* are higher than those for *vncp2lcord*), reaching a peak performance of 80% sensitivity and specificity rates (see Figure 2 and Table S3). Therefore, the support vector machine (SVM) classifiers obtained with the five most complex functional units, namely *mat2pep, aep2egut, pep2ebrain, tma2smusclep,* and *vna2lcord* (Table 1), were used as base classifiers to generate de novo localization predictions from microarray data.

### Verification of De Novo Predictions

We carried out a series of verification tests on the results of de novo predictions checking against: (1) literature curations from FlyBase [17]; (2) GO:Biological Process annotations from FlyBase; and (3) manual literature examination for a small number of confident predictions.

FlyBase has curated literature data on the spatiotemporal localization of gene expression for approximately 3,912 gene transcripts. Importantly, the FlyBase annotations are independent from the BDGP in situ hybridization annotations. In FlyBase, expression patterns are annotated via manual curation of published papers. Consequently, the annotation of expression patterns is diverse and both less systematic and less specific than those in the BDGP database. For example, the term “lateral cord” is widely used for annotating BDGP data, but it is never used in FlyBase. The *Drosophila* gross anatomy ontology shows that lateral cord is a “part of” the ventral nerve cord; therefore, the term “ventral nerve cord” is used in our verification analysis. Similarly, if an anatomical structure is used by the BDGP curators but does not have an exact match in FlyBase, we looked either for a higher-level anatomical structure or for a biological process in which the organ is involved. For example, according to anatomy ontology, embryonic brain is a “part of” the procephalic neurogenic region. Therefore, genes for which there is FlyBase curated literature evidence of expression in the procephalic neurogenic region, protocerebrum primordium, and procephalic ectoderm anlage may be used to verify predictions for genes in the *pep2ebrain* functional unit.

**Figure 3:**
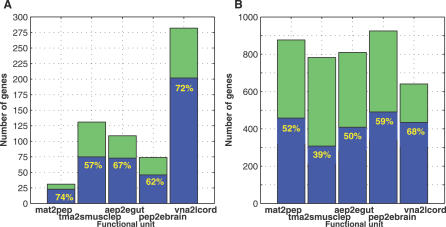
Verification of De Novo Gene Expression Localization Predictions Obtained by Applying Selected SVM Classifiers to Microarray Data (A) Verification using FlyBase literature data. The total height of each bar represents the number of genes associated with each functional unit according to FlyBase. The fraction of these correctly predicted by our classifiers is shown in blue. For the remainder (green fraction), the classifier makes negative predictions. (B) Verifications using the GO:Biological Process hierarchy. Here, the height of the bars corresponds to the total number of positive predictions from the selected classifier. The fraction of these predictions supported by GO annotations is in blue, and the remainder is shown in green. As annotation information does not constitute a complete picture, we cannot reliably estimate true-positive and false-positive rates in the conventional sense from such literature verifications (see Materials and Methods), but these values can be extrapolated from the operating point of the ROC curves.

The results of the prediction verifications are summarized in Figure 3A. Two functional units, *mat2pep* and *vna2lcord,* which encompass the formation of embryonic central brain precursors in early embryogenesis and development of the embryonic CNS, show the best overall literature verification scores of 74% and 72%, respectively. The lowest score is seen with the *tma2smusclep* functional unit (57%). The reason for this low score appears to be a significant disagreement in controlled vocabularies for these tissues between FlyBase and the BDGP databases. For the remaining two functional units considered, the enrichment in confirmed de novo predictions exceeds 60%. Since some tissues are more closely studied than others, calculating the fraction of false-positive predictions is difficult because failure to find annotation support for a prediction may simply reflect the fact that a given gene has yet to have its expression characterised.

The number of genes annotated with FlyBase literature data is small for some of the functional units considered (e.g., *mat2pep* and *pep2ebrain*); hence, only a small proportion of the de novo predictions can be validated by this route. However, since each of the functional units corresponds to a specific developmental process, we performed additional verifications using GO terms [18] drawn from the “Biological Process” category. A total of 14,332 proteins have GO annotations in FlyBase; of these, 2,794 have a GO:Biological Process term [19]. Fortunately, the majority of the GO:Biological Process terms are annotated by database curators rather than inferred by electronic annotation, and are thus more likely to be accurate [18]. We therefore used the GeneMerge software tool [20], which identifies and ranks GO terms that are statistically overrepresented with respect to a background distribution calculated from all genes with annotated GO:Biological Process terms. The statistical significance cutoff is set at a corrected e-score value of 0.05.

On the whole, the data on GO:Biological Process terms representation confirm the prediction results obtained with our classification method, providing support for predictions that have no FlyBase literature annotation (Table 2). For example, the GO:Biological Process annotation for the *mat2pep* functional unit assembled with genes controlling early development is enriched for relevant GO terms, such as blastoderm segmentation, formation of embryonic brain, and CNS precursors (Table 2). However, our analysis revealed several functional units, such as *aep2egut,* for which no overrepresented GO terms were found. FlyBase's QueryBuilder tool (http://flybase.org/cgi-bin/qbgui.fr.html), a user interface that supports powerful searches by offering access to every data field in FlyBase, provides support for the suggestion that there is a deficiency in annotations for the development of the *Drosophila* digestive tract. According to FlyBase's GO:Biological Process annotation, the number of genes known to be involved in endoderm development is 16, while the number of genes known to control midgut development is only 25. We suggest that a paucity of biological knowledge on the development of the digestive system reflects this in poor GO annotation, even for genes where either in situ hybridization data or literature data are available (i.e., in the groups of genes forming the training set and confirmed de novo prediction set, respectively; Table S1).

**Table 2:**
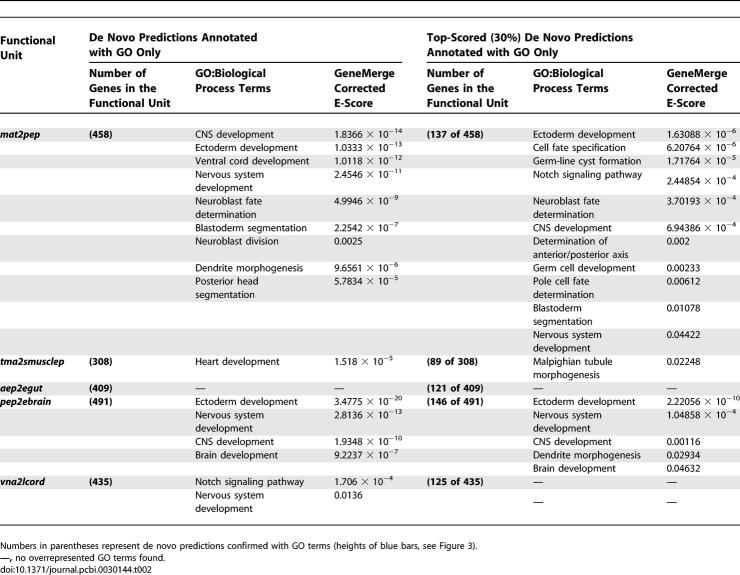
Verification of De Novo Predictions of Localisation of Gene Expression with GO:Biological Process and FlyBase Literature Data

As shown in Figure 3B, the GO annotation provides evidence supporting approximately 40% to 70% of the de novo predictions obtained with our method, depending on the functional unit in question. For the remainder, the genes are either not annotated with a GO term, or the predicted expression localisation is not correct. Although microarray profiles for the genes in the selected functional units are separable with our classifier model, many genes are located in the immediate vicinity of the hyperplane, separating positive and negative predictions (Figure S3). A consequence of the geometric structure of the SVM classifiers is that the prediction confidence increases with the distance of the gene from the hyperplane in multidimensional space. Hence, for every functional unit, we selected the highest-scoring 30% (i.e., those predictions furthest from the hyperplane) and reanalysed this set with GeneMerge. As expected (Table 2), for the majority of the functional units, the GO:Biological Process annotation becomes more specific. For example, genes in the *pep2ebrain* functional unit are enriched with the dendrite morphogenesis GO term, a lower-level GO term compared with brain development. However, for the top predictions in the *vna2lcord* functional unit, no overrepresented GO terms were found. Manual inspection of the GO annotations for these 125 genes (Table S11) indicates that 24% of the predictions are supported with GO:Biological Process terms related to CNS development. Thus, the GO:Biological Process annotation provides additional evidence to support the validity of our method for predicting spatial localization of gene expression.

De novo predictions of localization of expression for the functional units of interest obtained with SVM classifiers can be found in Tables S8–S12. As with the training sets, only a small number of genes associated with de novo localisation predictions belong to multiple functional units (see Figures S16 and S17B). However, the number of genes that, according to de novo predictions are simultaneously attributed to different functional units in late embryogenesis, increases. A surprisingly large overlap in shared de novo predictions is found for genes in the *aep2egut* and *pep2ebrain* functional units (see Table S13). An analysis of the shared genes using GeneMerge revealed significant enrichment for three GO terms: epidermal growth factor receptor signaling pathway, peripheral nervous system development, and plasma membrane. Unfortunately, a paucity of literature or GO annotation data on expression patterns for most of these genes prevents us from coming to an unambiguous conclusion on their role in development. However, we suggest that genes from this set may be largely responsible for cell proliferation, cell migration, cell adhesion, and attachment to the extracellular matrix during the development of both the nervous system and midgut.

### Evidence from Published Experiments

Undoubtedly, an in situ hybridisation is the most reliable test for any prediction of expression localization. We therefore searched the literature for published evidence to support our predictions. Thorough analysis of available literature data, electronic resources, and BDGP in situ experiments produced a list of 38 genes for which the expression localization is very likely to be correctly predicted (Tables S4–S7). In some of these cases, the literature evidence in support of the expression prediction confirms the localization of expression in a higher-level or lower-level anatomical structure. For some of the genes in this list, a BDGP in situ experiment exists; however, due to the problems with the annotations, these genes have not been used in our classifier training set. Recovering such genes further supports the utility of our prediction method.

For example, according to our prediction, *selenide,water dikinase (SelD)* is expressed in anatomical structures that form the digestive system, including the embryonic midgut && anterior midgut primordium && anterior endoderm primordium (*aep2egut* functional unit). Persson et al. [21] report *SelD* expression in the endodermal anlagen, midgut primordium, and in the gastric caecum. The latter structure is a “part of” the embryonic midgut, while the first is a precursor of the anterior endoderm primordium. Therefore, our predicted expression localisation is confirmed.

In the case of *chiffon (chif),* we predict expression in the ventral neuroderm anlage && ventral nerve cord primordium && lateral cord functional unit *(vna2lcord).* The BDGP flagged their in situ experiment for *chif* as ubiquitous expression; as a consequence, *chif* was not part of the training set used for classification. However, the BDGP in situ images are annotated, and clearly show elevated *chif* expression in the ventral nerve cord primordium, the ventral nerve cord, and the ventral ectoderm anlage. Since the BDGP-controlled vocabulary annotates the lateral cord as a “part of” the ventral nerve cord, our prediction of *chif* localization is consistent with the in situ data.

*Pendulin (Pen)* is predicted to belong to the *mat2pep* functional unit, and, in agreement with this, Torok et al. [22] report maternal expression as well as zygotic expression at blastoderm and in precephalic, cephalic, and ventral neuroectoderms. Similarly, we predict that *Minichromosome maintainence 7 (Mcm7)* belongs to the *mat2pep* functional unit; maternal expression is reported by Ohno et al. [23], while the evidence of strong expression in the embryonic central brain and CNS (Feger et al. [24]) implies early ubiquitous expression in precephalic, cephalic, and trunk neuroectoderms.

*wingless (wg)* expression is predicted in the embryonic central brain and its developmental precursors *(pep2ebrain).* Although the in situ experiment for *wg* exists in the BDGP database, the expression in embryonic central brain is not annotated. Therefore, this gene was not used in training of the SVM classifier and can be used to verify prediction results. Baker et al. [25] found *wg* to be expressed in the procephalic lobe, while Shmidt-Ott et al. [26] detected *wg* expression in the antennal, labral, and intercalary segments of the embryonic brain, thus supporting the prediction results obtained.

These observations support the utility of our predictions for particular developmental processes. However, we also noticed that some genes are predicted to be members of more than one functional unit. For example, *homothorax (hth), spitz (spi),* and *bangles and beads (bnb)* are associated with the *mat2pep* and *pep2ebrain* functional units, predictions supported by the published literature (see Tables S4, S6, S8, and S10). These functional units encompass a set of anatomical structures that reflects 16 stages of *Drosophila* embroyogenesis from fertilization to the differentiated embryonic central brain. This suggests that classification experiments designed to predict expression in a wider set of anatomical structures, reflecting developmental lineage from early developmental intermediates to the terminally differentiated anatomical structure, may also be successful. Furthermore, the ability to classify genes according to functional units overcomes the problems of divisive or agglomerative clustering approaches that force genes into a single cluster.

Finally, we note that in the case of *Semaphorin-2a (Sema-2a),* published in situ expression data by Kolodkin et al. [27] contradicts the BDGP in situ experiment. Kolodkin et al. report *Sema-2a* expression beginning at stage 10 and localised primarily in the developing CNS. On the other hand, the BDGP database reports maternally derived expression at the cellular blastoderm but not in the CNS. Our prediction indicates that *Sema-2a* belongs to the *mat2pep* functional unit, supporting the BDGP in situ experiment, and implies localisation of expression in precursors of the developing embryonic central brain, a “part of” the CNS. Again, since the BDGP annotation at later stages of development is ubiquitous, this gene was not used in our training set.

### Conclusions

By formulating a supervised learning framework, we have been able to predict tissue-specific gene expression in some sets of anatomical structures with high accuracy, achieving 80% sensitivity and specificity rates for the sets of anatomical structures we selected.

Our de novo predictions were verified using curated literature data, GO: Biological Process annotations, and published experimental reports. In the case of genes for which annotated in situ expression patterns are available, we achieve a true positive rate of 60%–70% for most of the functional units considered. In addition, we observed clear enrichments for annotations that are assigned to morphogenetic events arising from the processes governed by the products of genes in the functional unit. Finally, prediction results obtained for genes such as *chif, SelD, Pen, Mcm7, wg,* and an additional 33 genes were verified with published in situ hybridization experiments. As we described for the case of *Sema-2a,* our method may also have utility for automatically improving existing annotations in the BDGP or FlyBase databases by detecting potential anomalies or annotation conflicts.

The approach we present in this study allows us to combine qualitative information on gene expression from in situ hybridisation studies or gross anatomy ontologies with semiquantitative descriptions of gene expression dynamics obtained from microarray experiments to identify the tissue localization of gene expression on a large scale. We further note that the classification approach we apply here is superior to other, more frequently used, methods of function prediction from microarray gene expression data, such as cluster analysis or principal component analysis (see Figure S9 and a discussion on results of the benchmarking study on discriminant versus projection methods accompanying this Figure). This is because we are able to use prior knowledge in the form of class labels associated with functional units and infer complex correlations from the microarray measurements [13,28].

The classification approach proposed here is subject to two distinct limitations. First, superior performance results are obtained for genes that are characterized with complex continuous microarray gene expression profiles. Not only are these expression signatures better recognized by our classification method, but also the functional units assembled with such profiles are more homogeneous, facilitating faster classifier training and enhancing the performance of the method. In addition, the predictive power of the method and the range of developmental process for which the localisation of gene expression are possible depend on the time resolution of the microarray experiment (i.e., number of microarray time points available for analysis). Second, many high-throughput in situ experiments are accompanied by high-quality matching microarray datasets; however, this is not always the case. If this analysis were to be repeated for another organism for which spatial expression patterns are documented by in situ photomicrographs only, there may be complications due to data integration issues. For example, synchronization of microarray expression data obtained with different experimental platforms, blending time points, and corresponding expression values from various time courses, etc.

Recognising these limitations, however, our prediction method is versatile and can be applied to any microarray and in situ hybridization data regardless of the organism and biological process under study. The increasing efforts aimed at cataloging the spatiotemporal patterns of gene expression in vertebrates (i.e., Neidlhard et al. [29] and Yoshikawa et al. [30]), where the collection and analysis of in situ data are more difficult than with *Drosophila,* combined with the increasing quantities of available gene expression data, suggest an immediate application for our classification method. The in situ expression patterns, however, should be exhaustively characterized with a list of features in order to facilitate the classification.

In summary, we believe our tool offers a flexible method for increasing the utility of high-throughput gene expression data. The ability to combine different data modalities for integrative data mining is becoming increasingly important as we seek to translate the information encoded in genome sequence into biological function. By facilitating improved functional annotation of transcription units, our method provides an important way of adding value to expression data without the need for expensive experimental approaches.

## Materials and Methods

### Data.

Whole-genome Affymetrix microarray developmental time-course data corresponding to 11,904 genes and in situ hybridization data of 2,500 experiments originating from BDGP were obtained from ftp.fruitfly.org. With the in situ Expression Patterns database release used here (Release 2, 5 April 2004), experiments documented as “junk,” “no staining,” “production problem,” “too weak,” “ubiquitous,” and “maternal” were removed, reducing the set to 1,875 experiments corresponding to 1,565 unique microarray gene expression profiles. The in situ experimental data was annotated using a controlled vocabulary for anatomical structures with photomicrographs ordered according to stages of development. The BDGP annotations were collected by visual inspection of the images, resulting in six temporal classes corresponding to groups of developmental stages.

The whole-genome microarray data (ArrayExpress accession E-RUBN-2 [31]) were produced by BDGP. RNA samples were prepared from whole-embryo homogenates and cDNA-hybridized to Affymetrix GeneChip *Drosophila* Genome Arrays (http://www.affymetrix.com) using standard protocols and equipment. The samples were taken every hour, starting from 30 min after fertilization. The scanned array images were analyzed by Tomancak et al. [9] using the RMA algorithm [32] as implemented in the open source package Bioconductor [33]. In total, there were 36 array scans comprising three independent replicate time series for each gene. The expression levels were averaged among the replicates to obtain final expression values for 12 time points that correspond to 15 developmental stages.

### Construction of the functional units.

Construction of functional units was not automated due to differentiation of tissues and organs, uncertainties in annotation of in situ data, and technical issues arising with the use of microarrays in developmental biology. Identifying correlation dependencies between microarray gene expression profiles and in situ hybridization staining patterns was not straightforward for numerous reasons (see Tomancak et al. [9] for a detailed discussion). The in situ staining used in BDGP experiments is performed with an enzymatic reaction; therefore, the staining intensity is dependent not only on the strength of the probe, but also on the amount of time the color reaction is developed. Short staining times may result in weak ubiquitous expression that may not be detected in a whole-organism microarray experiment. In addition, the total amount of RNA produced by small anatomical structures may not be sufficient to be detected as an expression level change in a whole-organism microarray. Therefore, a selection of anatomical structures in which microarray experiments are expected to be capable of detecting gene expression changes were obtained by visual inspection of in situ photomicrographs and corresponding microarray profiles.

**Figure 4:**
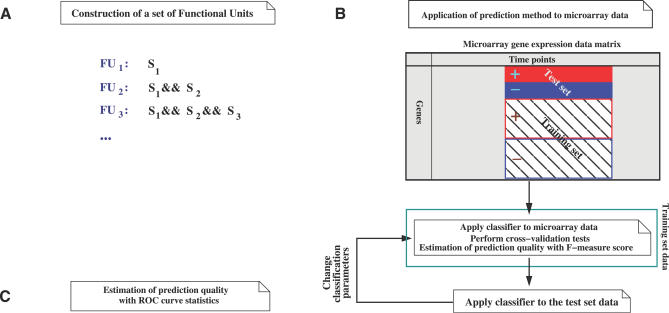
The Three-Stage Computational Process (A) The first stage is a construction of a functional unit. *S_i_*;*i* = 1,…,3 denotes anatomical structures selected to construct a functional unit. For instance, a functional unit corresponding to CNS development contains genes expressed in ventral neuroderm anlage && ventral nerve cord primordium && lateral cord (see Materials and Methods for details). (B) A bootstrapping and cross-validation experiment(s) are invoked to set up internal parameters for SVM classification. Shaded regions within the microarray gene expression data matrix define a time interval and a set of microarray data that corresponds to the functional unit. “+” and “−” represent positively and negatively labeled subsets of microarray expression data. (C) The third step refers to an estimation of prediction quality of the method with the ROC curve statistics.

Each of the above factors contribute to the small number of genes that are known to be involved in a specific developmental process of interest and in which variations in annotation of expression patterns are accompanied by changes in expression dynamics obtained in a microarray experiment. To choose a set of anatomical structures to assemble a functional unit, we selected microarray gene expression profiles that exhibit fluctuations during continuous intervals in the course of development (six to eight consecutive time points on the microarray time course; see Table 1 and Figures 4 and S2) and identified structures that display strong staining on the in situ photomicrograph and therefore are likely to contribute to the expression signal. A functional unit is assembled with a subset of structures linked by morphology and involved in a developmental process of interest.

**Figure 5:**
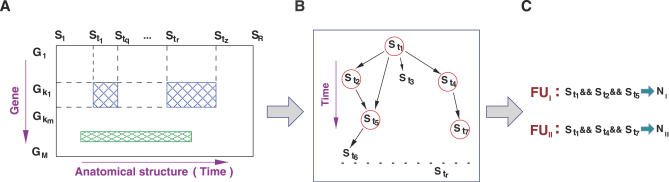
Construction of a Functional Unit (A) Results of the cluster analysis reported by Tomancak et al. [9] with the binary in situ gene expression matrix. This matrix constructed by parsing the BGDP Expression Patterns database allows the identification of groups of anatomical structures involved in key developmental processes during *Drosophila* embryogenesis (blue and green rectangles). (B) The developmental lineage of anatomical structures corresponding to the developmental process of interest is revealed by means of *Drosophila* gross anatomy ontology. Arrows represent “part of” and “develops from” relationships. Large anatomical structures are identified (red circles) by visual inspection of in situ photomicrographs. (C) Large anatomical structures related by morphological changes in embryogenesis are assembled into lists. A set of genes expressed in all anatomical structures comprising a list is called a functional unit (*FU_i_*). *N_i_* is a number of genes in functional unit *FU_i_*.

Functional units were constructed starting from the cluster analysis reported by Tomancak et al. [9], which identified sets of anatomical structures involved in key biological processes (see Figure 5A). To perform a clustering analysis with the in situ data, Tomancak et al. transformed the textual annotation of expression patterns into a binary matrix. Each gene in this matrix is annotated with respect to the presence or absence of expression with the in situ hybridization study. The output of the clustering analysis revealed developmental processes that are widely represented in the in situ dataset along with lists of genes associated with each process. Denoting a group of anatomical structures by *S_t_* = 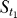 ,…,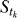 (Figure 5, shown in blue) and the corresponding sets of genes involved in the development of these structures by *G* = 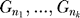 : (1) from the *Drosophila* gross anatomy ontology (http://obo.sourceforge.net/browse.html), we obtained the morphogenetic hierarchy of anatomical structures (Figure 5B); (2) by visual inspection of the in situ expression patterns and corresponding microarray gene expression profiles, we selected anatomical structures that were considered capable of producing sufficient amount of mRNA for reliable microarray measurements; (3) anatomical structures linked by “part of” or “develops from” relationships in the gross anatomy ontology were assembled into lists 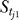 && 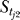 && 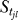 ; and (4) to facilitate reliable training of the learning methods, we restricted ourselves to developmental processes for which there are a reasonable number (at least 30) of genes known to be involved.

Functional units are the sets of genes that satisfy the above criteria.

### Classifier design.

SVMs are powerful class prediction techniques that have been used in a range of biological applications, including microarray data analysis problems [13,34–37]. Given a functional unit, we designed SVM classifiers in the space of microarray gene expression data (Figure 4B) using the implementation SVM*light* [38]. We used polynomial and radial basis function kernels to construct classifiers and optimized kernel width and cost-insensitive margin parameters in a cross-validation loop (Figure 4B) to maximize the F1 measure [39]. This is an appropriate objective function given a high imbalance between the numbers in each class. Data was partitioned at random into 50%–25%–25% for training, validation, and test purposes: the first 50% for optimizing the SVM weight parameters, the second 25% for cross-validation to select kernel width and margin parameters, and the final 25% to draw receiver operating characteristic (ROC) curves quantifying the performance of each classifier.

For many years now, since the pioneering works of Swets [40,41], ROC curves and the area under the ROC curve measure have been used to evaluate the performance of machine learning methods. We averaged the ROC curves of all bootstrap partitions to find a reliable operating point. Following Flach [42], we used vertical averaging of the ROC curves in 20 bins along the false-positive axis and used cubic spline interpolation to obtain an average ROC curve [43–46]. An operating point on this average curve was chosen by the projection method [47]. From among all the classifiers we designed, we selected the classifier closest to this operating point to perform de novo predictions.

With the resulting classifiers, we were able to identify genes that belonged to the defined functional units at a high level of accuracy. Discrimination achieved between genes in a functional unit and the remaining genes [48] is shown in the receiver operating characteristics curves in Figure 2. To confirm that the data distributions require nonlinear class boundaries achieved by SVMs, we also designed linear classifiers by the Fisher linear discriminant analysis (FLDA) method [49] and found significant gains in performance of the SVM classifiers in terms of the areas under the ROC curves. The result of comparison of the areas under the ROC curves measures either for the whole range of specificities or for the selected interval of specificities [43,44] and determines the classifier that discriminates in the given data space in the best possible way. The optimal point of the ROC curve that gives the best parameters of the classifier is calculated according to a standard projection method as described in [47]. The FLDA classifiers generally had lower average performances, but also showed lower variability across different bootstrap partitions of the data (Figures S4–S8).

We have verified de novo predictions generated by the classifier using both literature data curated by FlyBase and functional GO annotations. The verification is constrained by two factors: (1) the comparatively small number of genes with expression patterns annotated by FlyBase curators, and (2) possible ambiguities in GO annotations. Apart from these considerations, there is an inherent asymmetry in the functional annotation of genes. When a gene is reported to be expressed in a certain set of tissues, we may be confident that this is true. However, when such an association is absent, it may simply reflect the fact that experiments have not been conducted or the results have not been reported in the relevant databases. We thus estimate true-positive and false-positive rates for the de novo localisation predictions obtained with our classifier using the operating point of the ROC curves constructed to evaluate performance with the BDGP data (see Figures 2, S4–S8). In this case, both microarray and anatomical annotation data are available. These estimates are reliable given the assumption that the genes annotated in the BDGP in situ hybridization data are a representative sample of the *Drosophila* genome. The robustness of the classification model has been evaluated in extensive cross-validation and bootstrapping steps, rather than assessed on a specific pair of training and test sets; therefore, we expect the extrapolations to be reliable. The true-positive and false-positive rates are in the range of 70%–85% and 15%–30%, respectively, depending on the functional unit in question.

## Supporting Information

Dataset S1 GO:Biological Process Annotation for De Novo Prediction of Localization of Expression for the *mat2pep* Functional Unit A 30% fraction of genes are characterized with high prediction scores.
(40 KB XLS)

Dataset S2 GO:Biological Process Annotation for De Novo Prediction of Localization of Expression for the *tma2smusclep* Functional Unit A 30% fraction of genes are characterized with high prediction scores.
(28 KB XLS)

Dataset S3 GO:Biological Process Annotation for De Novo Prediction of Localization of Expression for the *pep2ebrain* Functional Unit A 30% fraction of genes are characterized with high prediction scores.
(42 KB XLS)

Dataset S4 GO:Biological Process Annotation for De Novo Prediction of Localization of Expression for the *vna2lcord* Functional Unit A 30% fraction of genes are characterized with high prediction scores.
(37 KB XLS)

Dataset S5 GO:Biological Process Annotation for De Novo Prediction of Localization of Expression for the *aep2egut* Functional Unit A 30% fraction of genes are characterized with high prediction scores.
(35 KB XLS)

Figure S1 Average Microarray Gene Expression Profiles with Respect to Sets of Anatomical Structures Selected to Form Functional Units (170 KB EPS)

Figure S2 Supervised Classification of Microarray Gene Expression Data (12 KB EPS)

Figure S3 Histograms of Distance from Separating Hyperplane for Genes for Which De Novo Predictions Are Made (75 KB EPS)

Figure S4 Estimation of Prediction Accuracy for Three Functional Units Constructed with Genes Involved in Early Development of *Drosophila*
(107 KB EPS)

Figure S5 Estimation of Prediction Accuracy for Three Functional Units Constructed with Genes Involved in Mesoderm Development in *Drosophila*
(108 KB EPS)

Figure S6 Estimation of Prediction Accuracy for Three Functional Units Constructed with Genes Involved in Intestinal Tract Development in *Drosophila*
(108 KB EPS)

Figure S7 Estimation of Prediction Accuracy for Three Functional Units Constructed with Genes Involved in Brain Development in *Drosophila*
(107 KB EPS)

Figure S8 Estimation of Prediction Accuracy for Three Functional Units Constructed with Genes Involved in CNS Development in *Drosophila*
(108 KB EPS)

Figure S9 Benchmarking Study on Discriminant Versus Projection Methods (PCA and FLDA) Applied to Prediction of Tissue-Specific Expression for Two Functional Units Constructed with Genes Involved in *Drosophila* CNS Development (174 KB EPS)

Figure S10 Diversity of Anatomical Annotation for Genes Comprising the *mat2pep* Functional Unit (1.1 MB EPS)

Figure S11 Diversity of Anatomical Annotation for Genes Comprising the *tma2smusclep* Functional Unit (1.3 MB EPS)

Figure S12 Diversity of Anatomical Annotation for Genes Comprising the *aep2egut* Functional Unit (1.8 MB EPS)

Figure S13 Diversity of Anatomical Annotation for Genes Comprising the *pep2ebrain* Functional Unit (2.2 MB EPS)

Figure S14 Diversity of Anatomical Annotation for Genes Comprising the *vna2lcord* Functional Unit (2.1 MB EPS)

Figure S15 Genes from the Training Set and Their Affiliation to the Five Most Complex Functional Units (33 KB EPS)

Figure S16 Genes from the De Novo Prediction Sets and Their Affiliation to the Five Most Complex Functional Units (70 KB EPS)

Figure S17 Number of Genes Associated with One or Many Functional Units from the Most Complex Set (22 KB EPS)

Table S1 GO Overrepresentation Scores for the Groups of Genes that Have Been Used to Train the SVM classifier, in Which Expression Pattern Is Documented in the BDGP Database (Training Set), and for Those Genes in Which In Situ Expression Pattern Is Annotated in FlyBase (83 KB PDF)

Table S2 Performance Measures for SVM and FLDA Classifiers Applied to Functional Units (78 KB PDF)

Table S3 Parameters of the Best Classifier Found with the Projection Method, and *Sensitivity* and *1-Specificity* Scores Obtained when Applying the Prediction Method for the Test Set Data (75 KB PDF)

Table S4 De Novo Predictions of Localization of Gene Expression for Functional Unit *mat2pep:* Procephalic Ectoderm Primordium && Cellular Blastoderm && Maternal Confirmed by Published Experiments (59 KB PDF)

Table S5 De Novo Predictions of Localization of Gene Expression for Functional Unit *vna2lcord:* Ventral Neuroderm Anlage && Ventral Nerve Cord Primordium && Lateral Cord Confirmed by Published Experiments (43 KB PDF)

Table S6 De Novo Predictions of Localization of Gene Expression for Functional Unit *pep2ebrain:* Embryonic Central Brain && Protocerebrum Primordium && Procephalic Ectoderm Primordium Confirmed by Published Experiments (63 KB PDF)

Table S7 De Novo Predictions of Localization of Gene Expression for Functional Unit *aep2egut:* Embryonic Midgut && Anterior Midgut Primordium && Anterior Endoderm Primordium Confirmed by Published Experiments (49 KB PDF)

Table S8 De Novo Prediction of Localization of Expression for the *mat2pep* Functional Unit A 30% fraction of genes are characterized with high prediction scores.
(80 KB PDF)

Table S9 De Novo Prediction of Localization of Expression for the *tma2smusclep* Functional Unit A 30% fraction of genes are characterized with high prediction scores.
(70 KB PDF)

Table S10 De Novo Prediction of Localization of Expression for the *pep2ebrain* Functional Unit A 30% fraction of genes are characterized with high prediction scores.
(80 KB PDF)

Table S11 De Novo Prediction of Localization of Expression for the *vna2lcord* Functional Unit A 30% fraction of genes are characterized with high prediction scores.
(79 KB PDF)

Table S12 De Novo Prediction of Localization of Expression for the *aep2egut* Functional Unit A 30% fraction of genes are characterized with high prediction scores.
(79 KB PDF)

Table S13 Number of Genes Simultaneously Attributed to Two Functional Units Encompassing Developmental Processes in Late Embryogenesis (50 KB PDF)

Table S14 Diversity of Anatomical Annotations of Genes Comprising the Functional Unit *mat2pep*
(94 KB PDF)

Table S15 Diversity of Anatomical Annotations of Genes Comprising the Functional Unit *tma2smusclep*
(79 KB PDF)

Table S16 Diversity of Anatomical Annotations of Genes Comprising the Functional Unit *vna2lcord*
(105 KB PDF)

Table S17 Diversity of Anatomical Annotations of Genes Comprising the Functional Unit *aep2egut*
(104 KB PDF)

Table S18 Diversity of Anatomical Annotations of Genes Comprising the Functional Unit *pep2ebrain*
(119 KB PDF)

### Accession Numbers

The Gene Ontology (http://www.geneontology.org) terms from the Biological Process category discussed in this paper are assigned with the following accession numbers: blastoderm segmentation (GO:0007350), endoderm development (GO:0007492), midgut development (GO:0007494), dendrite morphogenesis (GO:0048813), brain development (GO:0007420), epidermal growth factor receptor signaling pathway (GO: 0007173), peripheral nervous system development (GO:0007422). The Gene Ontology accession number for the term “plasma membrane” from the Cellular Component category is GO:005886.

The FlyBase (http://www.flybase.net) accession numbers for the genes discussed in this paper are *SelD* (FBgn0020615), *chif* (FBgn0000307), *Pen* (FBgn0011823), *Mcm7* (FBgn0020633), *wg* (FBgn0004009), *hth* (FBgn0001235), *spi* (FBgn0005672), *bnb* (FBgn0001090), and *Sema-2a* (FBgn0011260).

## Abbreviations

BDGP: Berkeley *Drosophila* genome project
CNS: central nervous system
FLDA: Fisher linear discriminant analysis
GO: Gene Ontology
ROC: receiver operating characteristic
SVM: support vector machine
