# Supplementary material for: Prediction of Gene Expression in Embryonic Structures of Drosophila melanogaster
Source: PLoS Comput Biol. 2007 Jul 20;3(7):e144. doi: 10.1371/journal.pcbi.0030144 (PMC1924873; doi:10.1371/journal.pcbi.0030144)
Supplement: Supplementary file 1 [file pcbi.0030144.st001.pdf]

Supplemental information for A.Samsonova *et al.* Prediction of gene expression in embryonic structures of *Drosophila melanogaster*.

Functional unit	**Training Set**		**Confirmed *de novo* Predictions**	
mat2pep	**(72)**		**(23)**	
	∅	∅	gastrulation	1.218E-7
			[‖]e & m interaction	2.445E-6
			ectoderm development	0.00223
tma2smusclep	**(35)**		**(75)**	
	myogenesis	1.78404E-4	mesoderm development	2.5152E-8
			heart development	9.7760E-5
			myoblast fusion	2.1031E-4
aep2egut	**(54)**		**(73)**	
	∅	∅	∅	∅
pep2ebrain	**(40)**		**(46)**	
	[‡‡]CNS development	1.59837E-5	nervous system development	2.4105E-7
	[††]NB fate determination	3.30863E-4	[**]GMC fate determination	8.6352E-4
	ectoderm development	0.04427	ectoderm development	0.0024
vna2lcord	**(34)**		**(202)**	
	CNS development	4.61455E-6	CNS development	9.3945E-25
	NB fate determination	1.35891E-4	ectoderm development	3.0268E-20
	ventral cord development	0.01054	ventral cord development	6.3021E-15
			nervous system development	1.1861E-11
			NB fate determination	8.8756E-9
			axon guidance	5.012E-8

[‡‡]Central nervous system
[‖]ectoderm & mesoderm interaction
[**]ganglion mother cell fate determination
[††]neuroblast fate determination

Table 1: GO over-representation scores for the groups of genes that have been used to train the SVM classifier, i.e. where expression pattern is documented in the BDGP database (Training set) and for those genes where *in situ* expression pattern is annotated in FlyBase. Each cell in the table contains the number of genes in every functional unit and GO:Biological Process terms that are detected to be over-represented in it. The over-representation scores (e-scores) are calculated with GeneMerge software. ∅ indicates that no over-represented GO terms are found for a given functional unit.
