# Supplementary material for: Prediction of Gene Expression in Embryonic Structures of Drosophila melanogaster
Source: PLoS Comput Biol. 2007 Jul 20;3(7):e144. doi: 10.1371/journal.pcbi.0030144 (PMC1924873; doi:10.1371/journal.pcbi.0030144)
Supplement: Supplementary file 2 [file pcbi.0030144.st002.pdf]

Functional units	SVM: Second order polynomial kernel vs. RBF kernel			FLDA
	interval	AUC_{P2}	AUC_{RBF}	AUC_{FLDA}
procephalic ectoderm primordium	[0.1; 0.25]§§<br>[0.05; 0.75]	0.0689<br>0.4456	0.0644<br>0.5019	0.0724<br>0.4726
procephalic ectoderm primordium && cellular blastoderm	[0.1; 0.5]§§<br>[0.05; 0.75]	0.3426<br>0.6080	0.3399<br>0.5962	0.27885<br>0.50597
procephalic ectoderm primordium && cellular blastoderm && maternal	[0.05; 0.75]	0.6439	0.6157	0.52397
somatic muscle primordium	[0.05; 0.85]	0.4635	0.5340	0.42789
somatic muscle primordium && trunk mesoderm primordium	[0.15; 0.4]§§<br>[0.05; 0.85]	0.1193<br>0.4508	0.1351<br>0.5435	0.12897<br>0.53123
somatic muscle primordium && trunk mesoderm primordium && trunk mesoderm anlage	[0.15; 0.4]§§<br>[0.05; 0.85]	0.1461<br>0.5257	0.1521<br>0.5282	0.1457<br>0.545725
embryonic central brain	[0.1; 0.9]	0.5402	0.4894	0.55671
embryonic central brain && protocerebrum primordium	[0.1; 0.9]	0.6166	0.5603	0.5378
embryonic central brain && protocerebrum primordium && procephalic ectoderm primordium	[0.1; 0.9]<br>[0.1; 0.35]§§	0.5981<br>0.1921	0.6126<br>0.1409	0.62716<br>0.14218
embryonic midgut	[0.05; 0.9]	0.5284	0.5650	0.45706
embryonic midgut && anterior midgut primordium	[0.05; 0.9]<br>[0.05; 0.5]§§	0.6529<br>0.2782	0.6339<br>0.2982	0.49321<br>0.17307
embryonic midgut && anterior midgut primordium && anterior endoderm primordium	[0.05; 0.9]<br>[0.05; 0.5]§§	0.6927<br>0.3279	0.6526<br>0.3198	0.53429<br>0.18929
lateral cord	[0.05; 0.8]	0.5332	0.3801	0.46408
lateral cord && ventral nerve cord primordium	[0.05; 0.8]<br>[0.05; 0.3]§§	0.6144<br>0.1644	0.5870<br>0.1707	0.63909<br>0.14939
lateral cord && ventral nerve cord primordium && ventral neuroderm anlage	[0.05; 0.8]<br>[0.05; 0.3]§§	0.6138<br>0.1538	0.6389<br>0.1625	0.57924<br>0.12837

Table 2: Performance measures for SVM and FLDA classifiers applied to functional units. P^2 denotes second order polynomial kernel and §§ symbol indicates an interval containing an x-coordinate of the operating point.
