# Supplementary material for: Prediction of Gene Expression in Embryonic Structures of Drosophila melanogaster
Source: PLoS Comput Biol. 2007 Jul 20;3(7):e144. doi: 10.1371/journal.pcbi.0030144 (PMC1924873; doi:10.1371/journal.pcbi.0030144)
Supplement: Supplementary file 3 [file pcbi.0030144.st003.pdf]

Functional units	Parameters of the classifier	*Se* and 1-*Sp* scores for the test data	
		Se	1-*Sp*
mat2pep	$\mathbf{P}^2$ c=0.55; s=1.4; r=1.2; j=1.1	0.8888	0.1905
tma2smusclep	**RBF** c=0.35; g=9.0; j= 3.7	0.875	0.1538
aep2egut	$\mathbf{P}^2$ c=0.1; s=3.0; r=0.7; j=1.2	0.8571	0.1176
pep2ebrain	$\mathbf{P}^2$ c=1.1; s=2.2; r=1.8; j=0.7	0.8571	0.1111
vna2lcord	**RBF** c=0.25; g=0.9; j=0.9	0.875	0.2

Table 3: Parameters of the best classifier found with the projection method and *Sensitivity* and *1-Specificity* scores obtained when applying the prediction method for the test set data. Here s,r and g are kernel parameters, while c is the parameter that controls a degree of training error tolerated. j denotes the misclassification cost factor. Kernel function used in classification (a) RBF: $K_{\mathbf{RBF}}(\vec{x}_1;\vec{x}_2) = \exp(-g(\vec{x}_1 - \vec{x}_2)^2)$ and (b) Second order polynomial ($\mathbf{P}^2$) $K_{\mathbf{P}^2}(\vec{x}_1;\vec{x}_2) = (s\,\vec{x}_1 \cdot \vec{x}_2 + r)^2$
