# Supplementary material for: Prediction of Gene Expression in Embryonic Structures of Drosophila melanogaster
Source: PLoS Comput Biol. 2007 Jul 20;3(7):e144. doi: 10.1371/journal.pcbi.0030144 (PMC1924873; doi:10.1371/journal.pcbi.0030144)
Supplement: Supplementary file 4 [file pcbi.0030144.st004.pdf]

De novo predictions of localization of gene expression confirmed by published experiments

Table 4: **Functional unit mat2pep**: procephalic ectoderm primordium && cellular blastoderm && maternal

Gene name	Prediction Rank	Expression	Reference
bnb	14	St.59; blastoderm ubiquitous.<br>St.8; Elevated posterior midgut invagination.<br>St.9; Dorsal epidermis, mesectoderm,<br>supra and subesophageal ganglia, brain<br>St.13; epidermal.	Ng *et al.* [1], Fig.5&6
mira	20	Maternal.<br>Blastoderm.<br>St.8; procephalic ventral neurectoderrm.<br>St.10; ventral nerve cord, brain,<br>posterior midgut.<br>St.13; brain and CNS.	Shen *et al.* [2], Fig 2.<br>Ikeshima-Kataoka *et al.* [3], Fig 2.a
rost	23	Maternal.<br>Blastoderm,<br>(Northern + statement, no image of blastoderm)<br>St.12: lateral and dorsal head, mesoderm.	Paulat *et al.* [4], Fig.3
BicD	34	Maternal. Ubiquitous.<br>Fades by end of blastoderm.<br>St.13; mesoderm and ventral midline.	Suter *et al.* [5], Fig.4
hth	35	Blastoderm.<br>St.6-7; ectoderm NOT procephalic<br>(posterior to cephalic furrow).<br>St.10-11; clypeolabrum, mandibular, labial.<br>St.14; CNS.	Kurant *et al.* [6], Fig.5<br>Rieckhof *et al.* [7], Fig.7<br><br>Nagao *et al.* [8], Fig.1
spi	47	Maternal.<br>Blastoderm.<br>St.12; enriched in mesodem (ubiquitous).<br>St.13; enriched in brain and CNS) (ubiquitous).	Rieckhof *et al.* [9] Fig.7
emb	57	Maternal.<br>Ubiquitous.<br>Blastoderm.	Collier *et al.* [10], Fig.2
Dl	66	Maternal.<br>Preblastoderm.<br>Blastoderm.<br>Procephalic and ventral neuroectoderm.	Haenlin *et al.* [11], Fig.5&6<br>Kooh *et al.* [12]<br>Kopczynski *et al.* [13]
D	71	NO maternal.<br>Preblastoderm.<br>Blastoderm.<br>Procepahlic and ventral neuroectoderm.	Russell *et al.* [14], Fig.3<br>Nambu *et al.* [15], Fig.5
CycB	78	Maternal.	Lehner *et al.* [16], Fig.4

continued on next page...

Table 4 continued from previous page.			
Gene name	Prediction Rank	Expression	Reference
		Ubiquitous.<br>Decreasing.<br>St.8-9 Cephalic and ventral neuroectoderm	
CycB3	103	Maternal.<br>Ubiquitous.<br>Blastoderm fading.<br>From St.7 cephalic and trunk mesoderm.	Jacobs *et al.* [17], Fig.4
gmc	104	NO maternal.<br>St.5; Blastoderm anteriorventral patch.<br>St.7; Anterior cephalic furrow and cephalic mesoderm analage<br>St.10; cephalic mesoderm.<br>St.10;glial precursors,<br>St.11; glia	Jones *et al.* [18], Fig.2<br>Bernardoni *et al.* [19], Fig.2& 3

References

[1] Ng SC, Perkins LA, Conboy G, Perrimon N, Fishman MC (1989) A drosophila gene expressed in the embryonic cns shares one conserved domain with the mammalian gap-43. Development 105:629–38.

[2] Shen CP, Jan LY, Jan YN (1997) Miranda is required for the asymmetric localization of prospero during mitosis in drosophila. Cell 90:449–58.

[3] Ikeshima-Kataoka H, Skeath JB, Nabeshima Y, Doe CQ, Matsuzaki F (1997) Miranda directs prospero to a daughter cell during drosophila asymmetric divisions. Nature 390:625–9.

[4] Paululat A, Goubeaud A, Damm C, Knirr S, Burchard S, et al. (1997) The mesodermal expression of rolling stone (rost) is essential for myoblast fusion in drosophila and encodes a potential transmembrane protein. J Cell Biol 138:337–48.

[5] Suter B, Romberg L, Steward R (1989) Bicaudal-d, a drosophila gene involved in developmental asymmetry: localized transcript accumulation in ovaries and sequence similarity to myosin heavy chain tail domains. Genes Dev 3:1957–1968.

[6] Kurant E, Pai CY, Sharf R, Halachmi N, Sun YH, et al. (1998) Dorsotonals/homothorax, the drosophila homologue of meis1, interacts with extradenticle in patterning of the embryonic pns. Development 125:1037–48.

[7] Rieckhof G, Casares F, Ryoo H, Abu-Shaar M, Mann R (1997) Nuclear translocation of extradenticle requires homothorax, which encodes an extradenticle-related homeodomain protein. Cell 91:171–183.

[8] Nagao T, Endo K, Kawauchi H, Walldorf U, Furukubo-Tokunaga K (2000) Patterning defects in the primary axonal scaffolds caused by the mutations of the extradenticle and homothorax genes in the embryonic drosophila brain. Development Genes and Evolution 210:289–299.

[9] Rutledge BJ, Zhang K, Bier E, Jan YN, Perrimon N (1992) The drosophila spitz gene encodes a putative egf-like growth factor involved in dorsal-ventral axis formation and neurogenesis. Genes Dev 6:1503–17.

[10] Collier S, Chan HY, Toda T, McKimmie C, Johnson G, et al. (2000) The drosophila embargoed gene is required for larval progression and encodes the functional homolog of schizosaccharomyces crm1. Genetics 155:1799–807.

[11] Haenlin M, B K, Campos-Ortega J (1990) The pattern of transcription of the neurogenic gene delta of drosophila melanogaster. Development 110:905–914.

[12] Kooh PJ, Fehon RG, Muskavitch MA (1993) Implications of dynamic patterns of delta and notch expression for cellular interactions during drosophila development. Development 117:493–507.

[13] Kopczynski C, Muskavitch M (1989) Complex spatio-temporal accumulation of alternative transcripts from the neurogenic gene delta during drosophila embryogenesis. Development 107:623–636.

[14] Russell SR, Sanchez-Soriano N, Wright CR, Ashburner M (1996) The dichaete gene of drosophila melanogaster encodes a sox-domain protein required for embryonic segmentation. Development 122:3669–76.

[15] Nambu P, Nambu J (1996) The drosophila fish-hook gene encodes a hmg domain protein essential for segmentation and cns development. Development 122:3467–75.

[16] Lehner CF, O'Farrell PH (1990) The roles of drosophila cyclins a and b in mitotic control. Cell 61:535–47.

[17] Jacobs HW, Knoblich JA, Lehner CF (1998) Drosophila cyclin b3 is required for female fertility and is dispensable for mitosis like cyclin b. Genes Dev 12:3741–51.

[18] Jones BW, Fetter RD, Tear G, Goodman CS (1995) glial cells missing: a genetic switch that controls glial versus neuronal fate. Cell 82:1013–23.

[19] Bernardoni R, Vivancos V, Giangrande A (1997) glide/gcm is expressed and required in the scavenger cell lineage. Dev Biol 191:118–130.
