# Supplementary material for: Prediction of Gene Expression in Embryonic Structures of Drosophila melanogaster
Source: PLoS Comput Biol. 2007 Jul 20;3(7):e144. doi: 10.1371/journal.pcbi.0030144 (PMC1924873; doi:10.1371/journal.pcbi.0030144)
Supplement: Supplementary file 5 [file pcbi.0030144.st005.pdf]

De novo predictions of localization of gene expression confirmed by published experiments

Table 5: **Functional unit vna2lcord**: ventral neuroderm anlage && ventral nerve cord primordium && lateral cord

Gene name	Prediction Rank	Expression	Reference
svp	23	St.8 Neuroblasts (neuroderm anlage).<br>Extensive NB expression.	Doe *et al.* [1], Fig.5<br>Broadus *et al.* [2], Fig.2

References

[1] Doe CQ (1992) Molecular markers for identified neuroblasts and ganglion mother cells in the drosophila central nervous system. Development 116:855–63.

[2] Broadus J, Doe C (1995) Evolution of neuroblast identity: seven-up and prospero expression reveal homologous and divergent neuroblast fates in drosophila and schistocerca. Development 121:3989–96.
