# Supplementary material for: Prediction of Gene Expression in Embryonic Structures of Drosophila melanogaster
Source: PLoS Comput Biol. 2007 Jul 20;3(7):e144. doi: 10.1371/journal.pcbi.0030144 (PMC1924873; doi:10.1371/journal.pcbi.0030144)
Supplement: Supplementary file 6 [file pcbi.0030144.st006.pdf]

De novo predictions of localization of gene expression confirmed by published experiments

Table 6: **Functional unit pep2ebrain**: embryonic central brain && protocerebrum primordium && procephalic ectoderm primordium

Gene name	Prediction Rank	Expression	Reference
gro	1	Maternal ubiquitous<br>St.8; broad distribution including procephalic ectoderm<br>St.11; brain and trunk CNS	Maier *et al.* [1], Fig.3<br>Hartley *et al.* [2], Fig.8
Nrt	13	St. 6; Dorsal and ventral ectoderm.<br>St 7; ventral furrow (mesoderm), cephalic furrow.<br>St. 9; Brain and trunk CNS.	Hortsch *et al.* [3], Fig.1,2&3
vvl	21	St.10; Midline and tracheal placodes<br>St.10-11; procephalon, clypeolabrum, labium (minor component of expression)	Anderson *et al.* [4],Fig.2
hth	26	Blastoderm.<br>St.6-7; ectodermNOT procephalic (posterior to cephalic furrow).<br>St.10-11; clypeolabrum, mandibular, labial.<br>St.14; CNS	Kurant *et al.* [5], Fig.5<br>Rieckhof *et al.* [6], Fig.7<br>Nagao *et al.* [7], Fig.1
18w	27	Blastoderm, procephalic region.<br>St.5; cepahalic furrow<br>St.8; procephalic ectoderm<br>St.12; clypeolabrum	Eldon *et al.* [8], Fig.1<br>Chiang *et al.* [9]
mts	28	procephalic neuroblasts<br>supraesophageal ganglion	Mayer-Jaekel *et al.* [10], Fig.7&8
mnb	33	Late (St.16) condensed trunk and brain CNS, supraesophageal ganglia	Tejedor *et al.* [11]. Fig.9
Fur1	76	Ventral nerve cord and brain.<br>St.14; antennomaxillary complex, subesophageal ganglion, clipeolabrum.	Hayflick *et al.* [12], Fig.9<br>Roebrook *et al.* [13], Fig.9
comm	78	Blastoderm pair rule pattern including procephalic ectoderm.<br>St.910; procephalic ectoderm and ventral nerve cord primordia	Tear *et al.* [14], Fig.4<br>BDGP
N	93	Blastoderm, neurogenic region.<br>Procephalic epidermis.<br>Supraesophageal ganglia.<br>Optic lobe primordia.	Hartley *et al.* [15], Fig.2
slp1	97	St.5; Blastoderm, Procephalic region.<br>St.6; Procephalon, head and cephalic furrow.<br>St.10; procephalon, clypeolabrum, gnathal<br>Ventral nerve cord and brain	Grossniklaus *et al.* [16], Fig.4

continued on next page...

Table 6 continued from previous page.			
Gene name	Prediction Rank	Expression	Reference
vnd	98	Blastoderm onwards:<br>ventral and procephalic neuroectoderm.<br>Brain and trunk neuroblasts.	Jimenez *et al.* [17], Fig.3
Mvl	101	Procephalic lobe, brain	Rodrigues *et al.* [18], Fig.7
disco	104	labrum, cephalic furrow.<br>procephalic.<br>gnathal segment.<br>clypolabrum.<br>optic and antennal brain lobes.	Lee *et al.* [19], Fig.1&2
tgo	112	Brain and ventral nerve cord.<br>Supraesophagheal ganglia.<br>Maxillary, labial,<br>mandibular and antennal segments.<br>antennal complex.	Sonnenfeld *et al.* [20], Fig.3<br>Emmons *et al.* [21], Fig.5
Nrk	128	St.11; cephalic and trunk neuroectoderm.<br>Brain and ventral nerve cord.	Oishi *et al.* [22], Fig.4
bnb	137	St.5-9; blastoderm ubiquitous.<br>St.8; Elevated posterior midgut invagination<br>St.9; Dorsal epidermis, mesectoderm.<br>supra and subesophageal ganglia.<br>Brain.<br>St13; epidermal.	Ng *et al.* [23], Fig.5&6
rst	144	Ventral midline.<br>Mandibular, maxillary and labial segments.<br>Clypeolabrum	Ramos *et al.* [24], Fig.4

References

[1] Maier D, Marte BM, Schafer W, Yu Y, Preiss A (1993) Drosophila evolution challenges postulated redundancy in the e(spl) gene complex. Proc Natl Acad Sci USA 90:5464–8.

[2] Hartley D, Preiss A, Artavanis-Tsakonas S (1988) A deduced gene product from the drosophila neurogenic locus, enhancer of split, shows homology to mammalian g-protein beta subunit. Cell 55:785–95.

[3] Hortsch M, Patel N, Bieber A, Traquina Z, Goodman C (1990) Drosophila neurotactin, a surface glycoprotein with homology to serine esterases, is dynamically expressed during embryogenesis. Development 110:1327–40.

[4] Anderson M, Perkins G, Chittick P, Shrigley R, Johnson W (1995) drifter, a drosophila pou-domain transcription factor, is required for correct differentiation and migration of tracheal cells and midline glia. Genes Dev 9:123–137.

[5] Kurant E, Pai CY, Sharf R, Halachmi N, Sun YH, et al. (1998) Dorsotonals/homothorax, the drosophila homologue of meis1, interacts with extradenticle in patterning of the embryonic pns. Development 125:1037–48.

[6] Rieckhof G, Casares F, Ryoo H, Abu-Shaar M, Mann R (1997) Nuclear translocation of extradenticle requires homothorax, which encodes an extradenticle-related homeodomain protein. Cell 91:171–183.

[7] Nagao T, Endo K, Kawauchi H, Walldorf U, Furukubo-Tokunaga K (2000) Patterning defects in the primary axonal scaffolds caused by the mutations of the extradenticle and homothorax genes in the embryonic drosophila brain. Development Genes and Evolution 210:289–299.

[8] Eldon E, Kooyer S, D'Evelyn D, Duman M, Lawinger P, et al. (1994) The drosophila 18 wheeler is required for morphogenesis and has striking similarities to toll. Development 120:885–99.

[9] Chiang C, Beachy P (1994) Expression of a novel toll-like gene spans the parasegment boundary and contributes to hedgehog function in the adult eye of drosophila. Mech Dev 47:225–239.

[10] Mayer-Jaekel RE, Baumgartner S, Bilbe G, Ohkura H, Glover DM, et al. (1992) Molecular cloning and developmental expression of the catalytic and 65-kda regulatory subunits of protein phosphatase 2a in drosophila. Mol Biol Cell 3:287–98.

[11] Tejedor F, Zhu XR, Kaltenbach E, Ackermann A, Baumann A, et al. (1995) minibrain: a new protein kinase family involved in postembryonic neurogenesis in drosophila. Neuron 14:287–301.

[12] Hayflick J, Wolfgang W, Forte M, Thomas G (1992) A unique kex2-like endoprotease from drosophila melanogaster is expressed in the central nervous system during early embryogenesis. J Neurosci 12:705–717.

[13] Roebroek AJ, Creemers JW, Pauli IG, Bogaert T, Van de Ven WJ (1993) Generation of structural and functional diversity in furin-like proteins in drosophila melanogaster by alternative splicing of the dfur1 gene. EMBO J 12:1853–70.

[14] Tear G, Harris R, Sutaria S, Kilomanski K, Goodman C, et al. (1996) commissureless controls growth cone guidance across the cns midline in drosophila and encodes a novel membrane protein. Neuron 12:501–514.

[15] Hartley DA, Xu TA, Artavanis-Tsakonas S (1987) The embryonic expression of the notch locus of drosophila melanogaster and the implications of point mutations in the extracellular egf-like domain of the predicted protein. EMBO J 6:3407–17.

[16] Grossniklaus U, Pearson RK, Gehring WJ (1992) The drosophila sloppy paired locus encodes two proteins involved in segmentation that show homology to mammalian transcription factors. Genes Dev 6:1030–51.

[17] Jimenez F, Martin-Morris L, Velasco L, Chu H, Sierra J, et al. (1995) vnd, a gene required for early neurogenesis of drosophila, encodes a homeodomain protein. EMBO J 14:3487–3495.

[18] Rodrigues V, Cheah PY, Ray K, Chia W (1995) malvolio, the drosophila homologue of mouse nramp-1 (bcg), is expressed in macrophages and in the nervous system and is required for normal taste behaviour. EMBO J 14:3007–20.

[19] Lee K, Freeman M, Steller H (1991) Expression of the disconnected gene during development of drosophila melanogaster. EMBO J 10:817–826.

[20] Sonnenfeld M, Ward M, Nystrom G, Mosher J, Stahl S, et al. (1997) The drosophila tango gene encodes a bhlh-pas protein that is orthologous to mammalian arnt and controls cns midline and tracheal development. Development 124:4571–4582.

[21] Emmons RB, Duncan D, Estes PA, Kiefel P, Mosher JT, et al. (1999) The spineless-aristapedia and tango bhlh-pas proteins interact to control antennal and tarsal development in drosophila. Development 126:3937–45.

[22] Oishi I, Sugiyama S, Liu ZJ, Yamamura H, Nishida Y, et al. (1997) A novel drosophila receptor tyrosine kinase expressed specifically in the nervous system. unique structural features and implication in developmental signaling. J Biol Chem 272:11916–23.

[23] Ng SC, Perkins LA, Conboy G, Perrimon N, Fishman MC (1989) A drosophila gene expressed in the embryonic cns shares one conserved domain with the mammalian gap-43. Development 105:629–38.

[24] Ramos RG, Igloi GL, Lichte B, Baumann U, Maier D, et al. (1993) The irregular chiasm c-roughest locus of drosophila, which affects axonal projections and programmed cell death, encodes a novel immunoglobulin-like protein. Genes Dev 7:2533–47.
