# Supplementary material for: Prediction of Gene Expression in Embryonic Structures of Drosophila melanogaster
Source: PLoS Comput Biol. 2007 Jul 20;3(7):e144. doi: 10.1371/journal.pcbi.0030144 (PMC1924873; doi:10.1371/journal.pcbi.0030144)
Supplement: Supplementary file 7 [file pcbi.0030144.st007.pdf]

De novo predictions of localization of gene expression confirmed by published experiments

Table 7: **Functional unit aep2egut**: embryonic midgut && anterior midgut primordium && anterior endoderm primordium

Gene name	Prediction Rank	Expression	Reference
ab	4	Anterior and posterior midgut<br>Proventriculus<br>foregut<br>Stomatogastric nervous system<br>ventral midline	Hu *et al.* [1], Fig.4
nop5	10	Midgut primordium<br>Midgut	Vorbruggen *et al.* [2], Fig.2
HLHm7	26	Posterior midgut primordium<br>stomatogastric nervous system	Knust *et al.* [3], Fig.5&7
CG6712	101	Embryonic gut	Lyko *et al.* [4], Fig.2

References

[1] Hu S, Fambrough D, Atashi JR, Goodman CS, Crews ST (1995) The drosophila abrupt gene encodes a btb-zinc finger regulatory protein that controls the specificity of neuromuscular connections. Genes Dev 9:2936–48.

[2] Vorbruggen G, Onel S, Jackle H (2000) Restricted expression and subnuclear localization of the drosophila gene dnop5, a member of the nop/sik family of the conserved rrna processing factors. Mech Dev 90:305–8.

[3] Knust E, Tietze K, Campos-Ortega J (1987) Molecular analysis of the neurogenic locus enhancer of split of drosophila melanogaster. EMBO J 6:4113–412.

[4] Lyko F, Whittaker AJ, Orr-Weaver TL, Jaenisch R (2000) The putative drosophila methyltransferase gene ddnmt2 is contained in a transposon-like element and is expressed specifically in ovaries. Mech Dev 95:215–7.
