# Supplementary material for: Prediction of Gene Expression in Embryonic Structures of Drosophila melanogaster
Source: PLoS Comput Biol. 2007 Jul 20;3(7):e144. doi: 10.1371/journal.pcbi.0030144 (PMC1924873; doi:10.1371/journal.pcbi.0030144)
Supplement: Supplementary file 8 [file pcbi.0030144.st008.pdf]

De novo prediction of localization of expression for the 30% fraction of genes characterized with high prediction scores

The *de novo* predictions of localization of expression for the 30% fraction of genes characterized with high prediction scores obtained for each functional unit are verified with Gene Ontology terms from the Biological Process category. As a rule a gene is annotated with many GO:Biological Process terms. The *de novo* prediction of localization of expression for a gene is confirmed with GO term (the respective gene is marked with "yes" sign in the tables below), if and only if, the gene is annotated with at least one GO:Biological process term relevant to the developmental process the anatomical structures comprising the functional unit under study are known to be involved in. If the GO:Biological process annotation does not contain evidence in support of prediction of localization of expression a gene is assigned with "Not Annotated" ("NA") symbol in the tables. The GO:Biological Process annotation for *Drosophila* genes can be found in the data support files for this manuscript or in FlyBase database [1] at www.flybase.net.

Table 8: **Results of prediction of localization of gene expression for functional unit mat2pep**: procephalic ectoderm primordium && cellular blastoderm && maternal

Prediction Rank	Supported with GO:Biological Process annotation	Gene ID	Gene name	Confidence Score (distance from the hyperplane)
1	yes	FBgn0011761	*dhd*	53.9952
2	NA	FBgn0015565	*yin*	52.5244
3	yes	FBgn0003015	*osk*	51.6832
4	yes	FBgn0000615	*exu*	44.1211
5	yes	FBgn0004649	*yl*	32.2157
6	yes	FBgn0013272	*Gp150*	30.9283
7	NA	FBgn0010288	*Uch*	30.2230
8	yes	FBgn0017561	*Ork1*	28.2992
9	yes	FBgn0002962	*nos*	27.6248
10	yes	FBgn0026320	*Tom*	26.5294
11	NA	FBgn0004873	*hts*	23.6667
12	NA	FBgn0015776	*nrv1*	22.4076
13	yes	FBgn0000166	*bcd*	22.1905
14	yes	FBgn0001090	*bnb*	21.7687
15	yes	FBgn0000216	*Brd*	21.7446
16	yes	FBgn0004364	*18w*	20.1106
17	NA	FBgn0000182	*BicC*	19.1090
18	NA	FBgn0005671	*Vha55*	18.9957
19	yes	FBgn0003970	*vas*	18.4916
20	yes	FBgn0021776	*mira*	18.1972
21	yes	FBgn0023094	*cyc*	16.2562
22	yes	FBgn0000180	*bib*	16.0548
23	NA	FBgn0011705	*rost*	15.8194
24	NA	FBgn0028519	*CG4500*	15.4161

continued on next page...

Table 8 continued from previous page.

Prediction Rank	Supported with GO:Biological Process annotation	Gene ID	Gene name	Confidence Score (distance from the hyperplane)
25	NA	FBgn0005771	*noc*	15.3789
26	yes	FBgn0004236	*αTub67C*	15.3351
27	yes	FBgn0002633	*HLHm7*	15.2871
28	NA	FBgn0000392	*cup*	15.2272
29	NA	FBgn0029174	*FKBP59*	14.8504
30	NA	FBgn0003187	*qua*	14.6951
31	yes	FBgn0010452	*trn*	14.2813
32	NA	FBgn0012037	*Ance*	14.2331
33	yes	FBgn0005695	*gcl*	14.0786
34	NA	FBgn0000183	*BicD*	13.7730
35	yes	FBgn0001235	*hth*	13.6069
36	NA	FBgn0000562	*egl*	13.5788
37	NA	FBgn0000097	*aop*	13.2934
38	NA	FBgn0004914	*Hnf4*	13.2717
39	yes	FBgn0000251	*cad*	13.1426
40	NA	FBgn0003204	*ras*	13.1191
41	yes	FBgn0000575	*emc*	12.9465
42	NA	FBgn0002673	*twe*	12.8778
43	NA	FBgn0019686	*lok*	12.7563
44	NA	FBgn0024846	*p38b*	12.5961
45	yes	FBgn0002629	*m4*	12.4736
46	yes	FBgn0000577	*en*	11.5534
47	NA	FBgn0005672	*spi*	11.5020
48	NA	FBgn0003023	*otu*	11.4709
49	yes	FBgn0014163	*fax*	11.3598
50	yes	FBgn0002631	*HLHm5*	11.2983
51	NA	FBgn0015239	*Hr78*	10.9673
52	NA	FBgn0002528	*LanB2*	10.6957
53	yes	FBgn0001254	*ImpE2*	10.5246
54	yes	FBgn0002561	*l(1)sc*	10.5130
55	yes	FBgn0003733	*tor*	10.2383
56	NA	FBgn0021765	*scu*	10.1786
57	NA	FBgn0020497	*emb*	9.7753
58	NA	FBgn0004872	*piwi*	9.5132
59	yes	FBgn0003117	*pnr*	9.4732
60	NA	FBgn0010316	*dap*	9.4639
61	yes	FBgn0014133	*bif*	9.4065
62	NA	FBgn0024234	*gbb*	9.3837
63	yes	FBgn0001987	*Gli*	9.3539
64	NA	FBgn0002069	*Aats-asp*	9.3367
65	yes	FBgn0011592	*fra*	9.2284
66	yes	FBgn0000463	*Dl*	8.8257
67	NA	FBgn0032693	*Cyp310a1*	8.7707
68	yes	FBgn0000011	*ab*	8.7675
69	NA	FBgn0004101	*bs*	8.6142
70	yes	FBgn0010105	*comm*	8.5711

continued on next page...

Table 8 continued from previous page.				
Prediction Rank	Supported with GO:Biological Process annotation	Gene ID	Gene name	Confidence Score (distance from the hyperplane)
71	yes	FBgn0000411	*D*	8.5129
72	yes	FBgn0000492	*Dr*	8.4884
73	yes	FBgn0004009	*wg*	8.4225
74	NA	FBgn0004362	*HmgD*	8.3760
75	NA	FBgn0002948	*nod*	8.2316
76	NA	FBgn0003507	*srp*	8.1902
77	NA	FBgn0011823	*Pen*	7.9986
78	NA	FBgn0000405	*CycB*	7.9496
79	yes	FBgn0000591	*E(spl)*	7.8492
80	NA	FBgn0003483	*spn-E*	7.6450
81	NA	FBgn0003884	α*Tub84B*	7.5595
82	NA	FBgn0001308	*Khc*	7.3953
83	NA	FBgn0001215	*Hrb98DE*	7.3923
84	yes	FBgn0002526	*LanA*	7.2772
85	yes	FBgn0002732	*m*α	7.2556
86	yes	FBgn0001325	*Kr*	7.2540
87	NA	FBgn0020633	*Mcm7*	7.1390
88	NA	FBgn0011604	*Iswi*	7.0895
89	yes	FBgn0003495	*spz*	7.0775
90	yes	FBgn0001075	*ft*	6.9668
91	NA	FBgn0015218	*eIF-4E*	6.9414
92	NA	FBgn0001257	*ImpL2*	6.8665
93	NA	FBgn0000150	*awd*	6.8446
94	yes	FBgn0004170	*sc*	6.7448
95	yes	FBgn0003430	*slp1*	6.4661
96	NA	FBgn0024923	*TER94*	6.4253
97	yes	FBgn0015380	*drl*	6.4185
98	NA	FBgn0020545	*kraken*	6.4003
99	yes	FBgn0005634	*zip*	6.3568
100	NA	FBgn0015765	*Mpk2*	6.3489
101	NA	FBgn0020369	*Pros45*	6.3325
102	NA	FBgn0003942	*RpS27A*	6.3138
103	NA	FBgn0015625	*CycB3*	6.3066
104	yes	FBgn0014179	*gmc*	6.2684
105	yes	FBgn0015805	*Rpd3*	6.1754
106	NA	FBgn0002524	*lace*	6.0995
107	NA	FBgn0003887	β*Tub56D*	6.0608
108	NA	FBgn0004237	*Hrb87F*	6.0451
109	yes	FBgn0003525	*stg*	5.9833
110	NA	FBgn0001941	*ifc*	5.9770
111	NA	FBgn0003471	β*-Spec*	5.9538
112	NA	FBgn0000927	*fs(1)Ya*	5.9130
113	NA	FBgn0000042	*Act5C*	5.8574
114	NA	FBgn0002283	*l(3)73Ah84D*	5.7829
115	NA	FBgn0003885	α*Tub84D*	5.7761
116	yes	FBgn0001148	*gsb*	5.7600

continued on next page...

Table 8 continued from previous page.				
Prediction Rank	Supported with GO:Biological Process annotation	Gene ID	Gene name	Confidence Score (distance from the hyperplane)
117	yes	FBgn0010349	*Dhc64C*	5.7546
118	yes	FBgn0011598	*grp*	5.7446
119	NA	FBgn0004381	*Klp68D*	5.7117
120	yes	FBgn0001123	*G-sα60A*	5.6853
121	yes	FBgn0011817	*nmo*	5.6682
122	NA	FBgn0020910	*RpL3*	5.6398
123	yes	FBgn0003498	*sqd*	5.5945
124	yes	FBgn0011260	*Sema-2a*	5.5443
125	NA	FBgn0015295	*shark*	5.5302
126	NA	FBgn0020622	*Pi3K21B*	5.5179
127	NA	FBgn0010438	*mtSSB*	5.4838
128	NA	FBgn0010410	*RpL27A*	5.4172
129	NA	FBgn0014857	*His3.3A*	5.3835
130	yes	FBgn0000308	*chic*	5.3499
131	NA	FBgn0004867	*sop*	5.3374
132	NA	FBgn0000158	*bam*	5.3253
133	yes	FBgn0004893	*bowl*	5.2975
134	NA	FBgn0003124	*polo*	5.2972
135	NA	FBgn0024939	*RpL8*	5.2501
136	NA	FBgn0011361	*mtacp1*	5.2215
137	yes	FBgn0010382	*CycE*	5.1200

References

[1] Drysdale RA, Crosby MA (2005) FlyBase: genes and gene models. Nucleic Acids Res 33:D390–5.
