# Supplementary material for: Prediction of Gene Expression in Embryonic Structures of Drosophila melanogaster
Source: PLoS Comput Biol. 2007 Jul 20;3(7):e144. doi: 10.1371/journal.pcbi.0030144 (PMC1924873; doi:10.1371/journal.pcbi.0030144)
Supplement: Supplementary file 9 [file pcbi.0030144.st009.pdf]

De novo prediction of localization of expression for the 30% fraction of genes characterized with high prediction scores

The *de novo* predictions of localization of expression for the 30% fraction of genes characterized with high prediction scores obtained for each functional unit are verified with Gene Ontology terms from the Biological Process category. As a rule a gene is annotated with many GO:Biological Process terms. The *de novo* prediction of localization of expression for a gene is confirmed with GO term (the respective gene is marked with "yes" sign in the tables below), if and only if, the gene is annotated with at least one GO:Biological process term relevant to the developmental process the anatomical structures comprising the functional unit under study are known to be involved in. If the GO:Biological process annotation does not contain evidence in support of prediction of localization of expression a gene is assigned with "Not Annotated" ("NA") symbol in the tables. The GO:Biological Process annotation for *Drosophila* genes can be found in the data support files for this manuscript or in FlyBase database [1] at www.flybase.net.

Table 9: **Results of prediction of localization of gene expression for functional unit tma2smusclep**: somatic muscle primordium && trunk mesoderm primordium && trunk mesoderm anlage

Prediction Rank	Supported with GO:Biological Process annotation	Gene ID	Gene name	Confidence Score (distance from the hyperplane)
1	NA	FBgn0024939	*RpL8*	0.9409
2	NA	FBgn0000356	*Cp16*	0.8065
3	yes	FBgn0001085	*fz*	0.6730
4	NA	FBgn0004428	*LysE*	0.6160
5	NA	FBgn0003301	*rut*	0.6044
6	NA	FBgn0004597	*CycC*	0.5519
7	NA	FBgn0003425	*sli*	0.5250
8	NA	FBgn0020414	*Idgf3*	0.5183
9	NA	FBgn0005776	*Pp2A-29B*	0.4790
10	yes	FBgn0004198	*ct*	0.4676
11	NA	FBgn0004050	*z*	0.4566
12	NA	FBgn0003520	*stau*	0.4273
13	NA	FBgn0004509	*Fur1*	0.3838
14	yes	FBgn0013343	*Syx1A*	0.3608
15	NA	FBgn0016696	*Pitslre*	0.3468
16	yes	FBgn0003986	*vnd*	0.3324
17	NA	FBgn0000221	*brn*	0.3080
18	NA	FBgn0019940	*Rh6*	0.2742
19	NA	FBgn0023495	*Lip3*	0.2714
20	NA	FBgn0001258	*ImpL3*	0.2707
21	NA	FBgn0002565	*Lsp2*	0.2696
22	NA	FBgn0027945	*ppl*	0.2658
23	yes	FBgn0014388	*sty*	0.2647
24	NA	FBgn0010110	*east*	0.2646

continued on next page...

Table 9 continued from previous page.

Prediction Rank	Supported with GO:Biological Process annotation	Gene ID	Gene name	Confidence Score (distance from the hyperplane)
25	NA	FBgn0001565	*Hlc*	0.2469
26	NA	FBgn0020261	*pcm*	0.2422
27	NA	FBgn0025739	*pon*	0.2327
28	NA	FBgn0010435	*emp*	0.2314
29	NA	FBgn0000409	*Cyt-c-p*	0.2244
30	NA	FBgn0011828	*Pxn*	0.2196
31	NA	FBgn0001079	*fu*	0.2188
32	NA	FBgn0010583	*dock*	0.2088
33	NA	FBgn0002948	*nod*	0.2030
34	NA	FBgn0003996	*w*	0.1966
35	NA	FBgn0015754	*Lis-1*	0.1914
36	NA	FBgn0003372	*Sgs1*	0.1896
37	NA	FBgn0003979	*Vm26Aa*	0.1884
38	NA	FBgn0011771	*Hem*	0.1859
39	NA	FBgn0000634	*Fas1*	0.1805
40	NA	FBgn0003660	*Syb*	0.1787
41	NA	FBgn0016718	*Reg-3*	0.1776
42	yes	FBgn0000606	*eve*	0.1774
43	NA	FBgn0004364	*18w*	0.1687
44	NA	FBgn0004379	*Klp67A*	0.1627
45	NA	FBgn0025741	*plexA*	0.1610
46	NA	FBgn0015954	*kuz*	0.1603
47	NA	FBgn0020224	*Cbl*	0.1500
48	NA	FBgn0003177	*pyd*	0.1444
49	yes	FBgn0003499	*sr*	0.1436
50	NA	FBgn0011706	*rpr*	0.1407
51	NA	FBgn0004574	*Rop*	0.1386
52	NA	FBgn0004449	*Ten-m*	0.1367
53	NA	FBgn0010410	*RpL27A*	0.1290
54	NA	FBgn0003302	*rux*	0.1288
55	NA	FBgn0020910	*RpL3*	0.1242
56	yes	FBgn0003749	*trh*	0.1236
57	NA	FBgn0025185	*az2*	0.1167
58	NA	FBgn0024754	*Flo*	0.1109
59	yes	FBgn0005592	*btl*	0.1080
60	NA	FBgn0026418	*Hsc70Cb*	0.0938
61	yes	FBgn0005558	*ey*	0.0903
62	yes	FBgn0000719	*fog*	0.0866
63	NA	FBgn0005624	*Psc*	0.0848
64	NA	FBgn0020391	*Nrk*	0.0829
65	NA	FBgn0015617	*Cdk7*	0.0818
66	NA	FBgn0004867	*sop*	0.0812
67	NA	FBgn0003124	*polo*	0.0787
68	NA	FBgn0020440	*Fak56D*	0.0787
69	NA	FBgn0004427	*LysD*	0.0785
70	yes	FBgn0003209	*raw*	0.0764

continued on next page...

Table 9 continued from previous page.				
Prediction Rank	Supported with GO:Biological Process annotation	Gene ID	Gene name	Confidence Score (distance from the hyperplane)
71	yes	FBgn0000659	*fkh*	0.0762
72	NA	FBgn0011818	*oaf*	0.0758
73	NA	FBgn0020378	*Sp1*	0.0744
74	NA	FBgn0020278	*loco*	0.0732
75	NA	FBgn0025680	*cry*	0.0728
76	NA	FBgn0005771	*noc*	0.0702
77	yes	FBgn0003310	*S*	0.0655
78	yes	FBgn0000014	*abd-A*	0.0639
79	NA	FBgn0026181	*rok*	0.0636
80	NA	FBgn0013809	*Dhc16F*	0.0636
81	NA	FBgn0003210	*rb*	0.0621
82	NA	FBgn0003411	*sisA*	0.0616
83	yes	FBgn0001180	*hb*	0.0612
84	NA	FBgn0010109	*dpn*	0.0595
85	NA	FBgn0000075	*amd*	0.0589
86	NA	FBgn0004622	*Takr99D*	0.0576
87	NA	FBgn0015295	*shark*	0.0494
88	NA	FBgn0011672	*Mvl*	0.0489
89	NA	FBgn0000566	*Eip55E*	0.0466

References

[1] Drysdale RA, Crosby MA (2005) FlyBase: genes and gene models. Nucleic Acids Res 33:D390–5.
