# Supplementary material for: Prediction of Gene Expression in Embryonic Structures of Drosophila melanogaster
Source: PLoS Comput Biol. 2007 Jul 20;3(7):e144. doi: 10.1371/journal.pcbi.0030144 (PMC1924873; doi:10.1371/journal.pcbi.0030144)
Supplement: Supplementary file 10 [file pcbi.0030144.st010.pdf]

De novo prediction of localization of expression for the 30% fraction of genes characterized with high prediction scores

The *de novo* predictions of localization of expression for the 30% fraction of genes characterized with high prediction scores obtained for each functional unit are verified with Gene Ontology terms from the Biological Process category. As a rule a gene is annotated with many GO:Biological Process terms. The *de novo* prediction of localization of expression for a gene is confirmed with GO term (the respective gene is marked with "yes" sign in the tables below), if and only if, the gene is annotated with at least one GO:Biological process term relevant to the developmental process the anatomical structures comprising the functional unit under study are known to be involved in. If the GO:Biological process annotation does not contain evidence in support of prediction of localization of expression a gene is assigned with "Not Annotated" ("NA") symbol in the tables. The GO:Biological Process annotation for *Drosophila* genes can be found in the data support files for this manuscript or in FlyBase database [1] at www.flybase.net.

Table 10: **Results of prediction of localization of gene expression for functional unit pep2ebrain**: embryonic central brain && protocerebrum primordium && procephalic ectoderm primordium

Prediction Rank	Supported with GO:Biological Process annotation	Gene ID	Gene name	Confidence Score (distance from the hyperplane)
1	yes	FBgn0001139	*gro*	13.8571
2	NA	FBgn0020496	*CtBP*	12.2987
3	yes	FBgn0014163	*fax*	12.1705
4	NA	FBgn0003498	*sqd*	11.8179
5	NA	FBgn0015622	*Cnx99A*	11.4479
6	yes	FBgn0010333	*Rac1*	11.4310
7	NA	FBgn0011760	*ctp*	11.2622
8	NA	FBgn0020910	*RpL3*	10.5409
9	NA	FBgn0010410	*RpL27A*	10.1206
10	NA	FBgn0003885	*αTub84D*	9.7893
11	NA	FBgn0014857	*His3.3A*	9.5983
12	yes	FBgn0003053	*peb*	9.5226
13	yes	FBgn0004108	*Nrt*	9.5063
14	NA	FBgn0001105	*Gβ13F*	9.4496
15	yes	FBgn0014184	*Oda*	9.3732
16	yes	FBgn0004907	*14-3-3ζ*	9.3489
17	NA	FBgn0004867	*sop*	9.2384
18	NA	FBgn0024939	*RpL8*	9.0460
19	NA	FBgn0000097	*aop*	8.6087
20	NA	FBgn0020615	*SelD*	8.5423
21	yes	FBgn0003995	*vvl*	8.4689
22	yes	FBgn0024250	*brk*	8.4506
23	NA	FBgn0015805	*Rpd3*	8.3159
24	NA	FBgn0028519	*CG4500*	8.1256

continued on next page...

Table 10 continued from previous page.				
Prediction Rank	Supported with GO:Biological Process annotation	Gene ID	Gene name	Confidence Score (distance from the hyperplane)
25	yes	FBgn0000308	*chic*	7.9656
26	yes	FBgn0001235	*hth*	7.8935
27	NA	FBgn0004364	*18w*	7.6850
28	NA	FBgn0004177	*mts*	7.5565
29	NA	FBgn0011817	*nmo*	7.5545
30	yes	FBgn0000413	*da*	7.5429
31	NA	FBgn0003471	*β-Spec*	7.5041
32	yes	FBgn0004606	*zfh1*	7.3404
33	yes	FBgn0002777	*mnb*	7.3242
34	NA	FBgn0004237	*Hrb87F*	7.2800
35	NA	FBgn0003941	*RpL40*	7.2751
36	NA	FBgn0003377	*Sgs7*	7.2304
37	NA	FBgn0003942	*RpS27A*	7.2268
38	yes	FBgn0003326	*sca*	7.1966
39	NA	FBgn0000405	*CycB*	7.0991
40	NA	FBgn0003501	*Src64B*	7.0519
41	yes	FBgn0003310	*S*	6.9174
42	NA	FBgn0003002	*opa*	6.9040
43	NA	FBgn0020369	*Pros45*	6.8997
44	NA	FBgn0003866	*tsh*	6.8960
45	NA	FBgn0003177	*pyd*	6.6653
46	NA	FBgn0004133	*blow*	6.6602
47	yes	FBgn0002945	*nkd*	6.6601
48	NA	FBgn0003887	*βTub56D*	6.5789
49	NA	FBgn0004362	*HmgD*	6.5614
50	NA	FBgn0004427	*LysD*	6.5324
51	NA	FBgn0015763	*lic*	6.5020
52	NA	FBgn0015239	*Hr78*	6.4408
53	NA	FBgn0013343	*Syx1A*	6.4082
54	NA	FBgn0023441	*fus*	6.3593
55	NA	FBgn0003963	*ush*	6.3319
56	NA	FBgn0000042	*Act5C*	6.2664
57	NA	FBgn0004003	*wbl*	6.1980
58	yes	FBgn0003892	*ptc*	6.1305
59	yes	FBgn0001077	*ftz*	6.0059
60	NA	FBgn0014011	*Rac2*	5.9803
61	NA	FBgn0000150	*awd*	5.9343
62	yes	FBgn0004053	*zen*	5.9191
63	NA	FBgn0014870	*Psi*	5.8882
64	NA	FBgn0003118	*pnt*	5.8771
65	yes	FBgn0026181	*rok*	5.7088
66	yes	FBgn0003651	*svp*	5.6886
67	NA	FBgn0005278	*M(2)21AB*	5.6299
68	NA	FBgn0027343	*fz3*	5.5626
69	NA	FBgn0003210	*rb*	5.5013
70	yes	FBgn0052372	*CG32372*	5.4465

continued on next page...

Table 10 continued from previous page.				
Prediction Rank	Supported with GO:Biological Process annotation	Gene ID	Gene name	Confidence Score (distance from the hyperplane)
71	yes	FBgn0004179	*Csp*	5.4383
72	yes	FBgn0000273	*Pka-C1*	5.4012
73	NA	FBgn0000046	*Act87E*	5.3949
74	NA	FBgn0041186	*Slbp*	5.3550
75	NA	FBgn0015625	*CycB3*	5.3462
76	NA	FBgn0004509	*Fur1*	5.3093
77	yes	FBgn0001104	*G-i*α*65A*	5.3028
78	yes	FBgn0010105	*comm*	5.2662
79	yes	FBgn0000340	*cno*	5.2191
80	yes	FBgn0005592	*btl*	5.1844
81	yes	FBgn0002973	*numb*	5.1702
82	yes	FBgn0024836	*stan*	5.1629
83	yes	FBgn0014343	*mirr*	5.1539
84	NA	FBgn0004425	*LysB*	5.1538
85	yes	FBgn0015380	*drl*	5.1158
86	NA	FBgn0000043	*Act42A*	5.1015
87	NA	FBgn0024234	*gbb*	5.0911
88	yes	FBgn0000499	*dsh*	5.0850
89	NA	FBgn0004828	*His3.3B*	5.0326
90	NA	FBgn0002989	*okr*	5.0274
91	NA	FBgn0016696	*Pitslre*	5.0183
92	NA	FBgn0012037	*Ance*	4.9827
93	yes	FBgn0004647	*N*	4.8935
94	NA	FBgn0001565	*Hlc*	4.8580
95	NA	FBgn0004399	*psq*	4.8255
96	NA	FBgn0003512	*Sry-*δ	4.8240
97	yes	FBgn0003430	*slp1*	4.8109
98	yes	FBgn0003986	*vnd*	4.7892
99	NA	FBgn0014028	*SdhB*	4.7829
100	NA	FBgn0038224	*CG3321*	4.7388
101	NA	FBgn0011672	*Mvl*	4.7353
102	yes	FBgn0005672	*spi*	4.7237
103	NA	FBgn0004456	*mew*	4.7229
104	yes	FBgn0000459	*disco*	4.7048
105	NA	FBgn0001404	*egh*	4.7037
106	yes	FBgn0000570	*elav*	4.6986
107	NA	FBgn0024923	*TER94*	4.6936
108	yes	FBgn0003749	*trh*	4.6703
109	NA	FBgn0015218	*eIF-4E*	4.6636
110	NA	FBgn0014388	*sty*	4.5951
111	NA	FBgn0025140	*pit*	4.5889
112	yes	FBgn0015014	*tgo*	4.5820
113	yes	FBgn0003391	*shg*	4.5685
114	NA	FBgn0001257	*ImpL2*	4.5494
115	NA	FBgn0020378	*Sp1*	4.5112
116	NA	FBgn0004551	*Ca-P60A*	4.5034

continued on next page...

Table 10 continued from previous page.				
Prediction Rank	Supported with GO:Biological Process annotation	Gene ID	Gene name	Confidence Score (distance from the hyperplane)
117	NA	FBgn0000615	*exu*	4.4831
118	NA	FBgn0032408	*CG6712*	4.4170
119	NA	FBgn0004449	*Ten-m*	4.4159
120	yes	FBgn0004009	*wg*	4.3906
121	NA	FBgn0010278	*Ssrp*	4.3492
122	NA	FBgn0002413	*dco*	4.3335
123	NA	FBgn0000568	*Eip75B*	4.2971
124	yes	FBgn0003165	*pum*	4.2872
125	NA	FBgn0013811	*Dhc62B*	4.2754
126	yes	FBgn0013765	*cnn*	4.2496
127	NA	FBgn0013974	*Gyc76C*	4.2472
128	yes	FBgn0020391	*Nrk*	4.2173
129	NA	FBgn0005624	*Psc*	4.2062
130	NA	FBgn0011715	*Snr1*	4.2045
131	yes	FBgn0003719	*tld*	4.1924
132	yes	FBgn0010575	*sbb*	4.1730
133	yes	FBgn0013272	*Gp150*	4.1729
134	NA	FBgn0003068	*per*	4.1613
135	NA	FBgn0004426	*LysC*	4.1175
136	NA	FBgn0024811	*Crk*	4.0998
137	NA	FBgn0001090	*bnb*	4.0960
138	yes	FBgn0003660	*Syb*	4.0479
139	yes	FBgn0010341	*Cdc42*	4.0164
140	NA	FBgn0015617	*Cdk7*	4.0100
141	NA	FBgn0004897	*fd96Ca*	3.9875
142	NA	FBgn0020278	*loco*	3.9615
143	yes	FBgn0001319	*kn*	3.9563
144	yes	FBgn0003285	*rst*	3.9456
145	NA	FBgn0000566	*Eip55E*	3.9351
146	NA	FBgn0017418	*ari-1*	3.9311

References

[1] Drysdale RA, Crosby MA (2005) FlyBase: genes and gene models. Nucleic Acids Res 33:D390–5.
