# Supplementary material for: Prediction of Gene Expression in Embryonic Structures of Drosophila melanogaster
Source: PLoS Comput Biol. 2007 Jul 20;3(7):e144. doi: 10.1371/journal.pcbi.0030144 (PMC1924873; doi:10.1371/journal.pcbi.0030144)
Supplement: Supplementary file 11 [file pcbi.0030144.st011.pdf]

De novo prediction of localization of expression for the 30% fraction of genes characterized with high prediction scores

The *de novo* predictions of localization of expression for the 30% fraction of genes characterized with high prediction scores obtained for each functional unit are verified with Gene Ontology terms from the Biological Process category. As a rule a gene is annotated with many GO:Biological Process terms. The *de novo* prediction of localization of expression for a gene is confirmed with GO term (the respective gene is marked with "yes" sign in the tables below), if and only if, the gene is annotated with at least one GO:Biological process term relevant to the developmental process the anatomical structures comprising the functional unit under study are known to be involved in. If the GO:Biological process annotation does not contain evidence in support of prediction of localization of expression a gene is assigned with "Not Annotated" ("NA") symbol in the tables. The GO:Biological Process annotation for *Drosophila* genes can be found in the data support files for this manuscript or in FlyBase database [1] at www.flybase.net.

Table 11: **Results of prediction of localization of gene expression for functional unit vna2lcord**: lateral cord && ventral nerve cord primordium && ventral neuroderm anlage

Prediction Rank	Supported with GO:Biological Process annotation	Gene ID	Gene name	Confidence Score (distance from the hyperplane)
1	NA	FBgn0002989	*okr*	0.5567
2	yes	FBgn0005654	*lat*	0.5146
3	NA	FBgn0001150	*gt*	0.5085
4	NA	FBgn0029088	*disp*	0.5055
5	yes	FBgn0003391	*shg*	0.5043
6	NA	FBgn0002283	*l(3)73Ah*	0.4992
7	NA	FBgn0000307	*chif*	0.4947
8	NA	FBgn0005596	*yemα*	0.4883
9	yes	FBgn0026181	*rok*	0.4755
10	NA	FBgn0019686	*lok*	0.4653
11	yes	FBgn0002932	*neur*	0.4530
12	NA	FBgn0003302	*rux*	0.4433
13	NA	FBgn0015763	*lic*	0.4284
14	NA	FBgn0014076	*Vm32E*	0.4283
15	yes	FBgn0000340	*cno*	0.4271
16	NA	FBgn0020249	*stck*	0.4251
17	NA	FBgn0003079	*phl*	0.4249
18	yes	FBgn0004053	*zen*	0.4164
19	yes	FBgn0000577	*en*	0.4162
20	NA	FBgn0003377	*Sgs7*	0.4120
21	NA	FBgn0014870	*Psi*	0.4033
22	NA	FBgn0000233	*btd*	0.3991
23	yes	FBgn0003651	*svp*	0.3989
24	NA	FBgn0000359	*Cp36*	0.3808

continued on next page...

Table 11 continued from previous page.

Prediction Rank	Supported with GO:Biological Process annotation	Gene ID	Gene name	Confidence Score (distance from the hyperplane)
25	NA	FBgn0003231	*ref(2)P*	0.3803
26	yes	FBgn0027343	*fz3*	0.3772
27	NA	FBgn0000146	*aub*	0.3766
28	NA	FBgn0000287	*salr*	0.3746
29	NA	FBgn0010357	*βTry*	0.3729
30	yes	FBgn0004179	*Csp*	0.3715
31	NA	FBgn0004399	*psq*	0.3696
32	NA	FBgn0003285	*rst*	0.3686
33	yes	FBgn0024250	*brk*	0.3672
34	yes	FBgn0015773	*NetA*	0.3633
35	yes	FBgn0003892	*ptc*	0.3485
36	yes	FBgn0014133	*bif*	0.3432
37	NA	FBgn0010278	*Ssrp*	0.3425
38	NA	FBgn0004636	*R*	0.3398
39	NA	FBgn0024234	*gbb*	0.3378
40	NA	FBgn0000927	*fs(1)Ya*	0.3369
41	NA	FBgn0000392	*cup*	0.3311
42	NA	FBgn0003200	*rap*	0.3291
43	NA	FBgn0002948	*nod*	0.3249
44	NA	FBgn0004897	*fd96Ca*	0.3208
45	NA	FBgn0004378	*Klp61F*	0.3173
46	NA	FBgn0015621	*Clp*	0.3115
47	NA	FBgn0000588	*esc*	0.3106
48	NA	FBgn0023441	*fus*	0.3089
49	NA	FBgn0039914	*mav*	0.3000
50	yes	FBgn0001978	*stc*	0.2996
51	NA	FBgn0016126	*CaMKI*	0.2965
52	NA	FBgn0004597	*CycC*	0.2951
53	NA	FBgn0003124	*polo*	0.2948
54	NA	FBgn0003117	*pnr*	0.2924
55	yes	FBgn0000221	*brn*	0.2903
56	NA	FBgn0022786	*Hira*	0.2900
57	NA	FBgn0003979	*Vm26Aa*	0.2900
58	NA	FBgn0001079	*fu*	0.2897
59	yes	FBgn0003169	*put*	0.2860
60	NA	FBgn0015553	*tos*	0.2815
61	NA	FBgn0003507	*srp*	0.2785
62	NA	FBgn0011672	*Mvl*	0.2701
63	NA	FBgn0013531	*MED20*	0.2608
64	NA	FBgn0013725	*phyl*	0.2496
65	NA	FBgn0027945	*ppl*	0.2490
66	NA	FBgn0003141	*pr*	0.2396
67	NA	FBgn0017551	*Rca1*	0.2391
68	NA	FBgn0020299	*stumps*	0.2305
69	NA	FBgn0025185	*az2*	0.2260
70	yes	FBgn0004110	*tin*	0.2223

continued on next page...

Table 11 continued from previous page.

Prediction Rank	Supported with GO:Biological Process annotation	Gene ID	Gene name	Confidence Score (distance from the hyperplane)
71	NA	FBgn0000404	*CycA*	0.2194
72	yes	FBgn0004795	*retn*	0.2190
73	NA	FBgn0002736	*mago*	0.2139
74	NA	FBgn0000490	*dpp*	0.2134
75	NA	FBgn0004644	*hh*	0.2109
76	yes	FBgn0025739	*pon*	0.2091
77	NA	FBgn0003310	*S*	0.2086
78	NA	FBgn0003177	*pyd*	0.2044
79	NA	FBgn0004379	*Klp67A*	0.2010
80	NA	FBgn0000719	*fog*	0.1994
81	NA	FBgn0027375	*RecQ5*	0.1967
82	yes	FBgn0010433	*ato*	0.1907
83	NA	FBgn0000061	*al*	0.1810
84	NA	FBgn0004367	*mei-41*	0.1795
85	yes	FBgn0005592	*btl*	0.1789
86	yes	FBgn0000163	*baz*	0.1775
87	NA	FBgn0013987	*MAPk-Ak2*	0.1750
88	NA	FBgn0000447	*Dhod*	0.1659
89	NA	FBgn0004838	*Hrb27C*	0.1635
90	NA	FBgn0003483	*spn-E*	0.1544
91	yes	FBgn0002631	*HLHm5*	0.1543
92	NA	FBgn0000356	*Cp16*	0.1524
93	yes	FBgn0013272	*Gp150*	0.1511
94	NA	FBgn0003250	*Rh4*	0.1501
95	NA	FBgn0000277	*CecA2*	0.1481
96	NA	FBgn0001565	*Hlc*	0.1470
97	NA	FBgn0020496	*CtBP*	0.1467
98	NA	FBgn0004360	*Wnt2*	0.1453
99	yes	FBgn0000368	*crb*	0.1443
100	NA	FBgn0004956	*os*	0.1442
101	NA	FBgn0003567	*su(Hw)*	0.1428
102	NA	FBgn0003204	*ras*	0.1424
103	NA	FBgn0019664	*pan*	0.1417
104	yes	FBgn0001325	*Kr*	0.1416
105	NA	FBgn0013269	*FK506-bp1*	0.1414
106	yes	FBgn0002552	*lin*	0.1395
107	NA	FBgn0002962	*nos*	0.1394
108	NA	FBgn0015513	*mbc*	0.1365
109	yes	FBgn0003520	*stau*	0.1360
110	NA	FBgn0002936	*ninaA*	0.1354
111	NA	FBgn0015816	*Slh*	0.1274
112	NA	FBgn0004895	*fd64A*	0.1251
113	NA	FBgn0027081	*Aats-thr*	0.1226
114	NA	FBgn0000409	*Cyt-c-p*	0.1225
115	NA	FBgn0000427	*dec-1*	0.1209
116	NA	FBgn0011293	*a10*	0.1204

continued on next page...

Table 11 continued from previous page.				
Prediction Rank	Supported with GO:Biological Process annotation	Gene ID	Gene name	Confidence Score (distance from the hyperplane)
117	yes	FBgn0000413	*da*	0.1192
118	yes	FBgn0003300	*run*	0.1188
119	NA	FBgn0002940	*ninaE*	0.1182
120	NA	FBgn0003205	*Ras85D*	0.1181
121	NA	FBgn0027094	*Aats-ala*	0.1172
122	NA	FBgn0003411	*sisA*	0.1162
123	NA	FBgn0003742	*tra2*	0.1145
124	NA	FBgn0003900	*twi*	0.1136
125	NA	FBgn0015011	*Ahcy89E*	0.1132

References

[1] Drysdale RA, Crosby MA (2005) FlyBase: genes and gene models. Nucleic Acids Res 33:D390–5.
