# Supplementary material for: Prediction of Gene Expression in Embryonic Structures of Drosophila melanogaster
Source: PLoS Comput Biol. 2007 Jul 20;3(7):e144. doi: 10.1371/journal.pcbi.0030144 (PMC1924873; doi:10.1371/journal.pcbi.0030144)
Supplement: Supplementary file 12 [file pcbi.0030144.st012.pdf]

De novo prediction of localization of expression for the 30% fraction of genes characterized with high prediction scores

The *de novo* predictions of localization of expression for the 30% fraction of genes characterized with high prediction scores obtained for each functional unit are verified with Gene Ontology terms from the Biological Process category. As a rule a gene is annotated with many GO:Biological Process terms. The *de novo* prediction of localization of expression for a gene is confirmed with GO term (the respective gene is marked with "yes" sign in the tables below), if and only if, the gene is annotated with at least one GO:Biological process term relevant to the developmental process the anatomical structures comprising the functional unit under study are known to be involved in. If the GO:Biological process annotation does not contain evidence in support of prediction of localization of expression a gene is assigned with "Not Annotated" ("NA") symbol in the tables. The GO:Biological Process annotation for *Drosophila* genes can be found in the data support files for this manuscript or in FlyBase database [1] at www.flybase.net.

Table 12: **Results of prediction of localization of gene expression for functional unit aep2egut**: embryonic midgut && anterior midgut primordium && anterior endoderm primordium

Prediction Rank	Supported with GO:Biological Process annotation	Gene ID	Gene name	Confidence Score (distance from the hyperplane)
1	NA	FBgn0000044	*Act57B*	16.6596
2	NA	FBgn0003885	*αTub84D*	8.4762
3	NA	FBgn0014163	*fax*	8.4354
4	NA	FBgn0000011	*ab*	8.4297
5	NA	FBgn0039039	*lmd*	7.7851
6	NA	FBgn0011823	*Pen*	6.9544
7	NA	FBgn0000405	*CycB*	6.9287
8	NA	FBgn0004362	*HmgD*	6.9259
9	NA	FBgn0026320	*Tom*	6.9029
10	NA	FBgn0026196	*nop5*	6.8920
11	NA	FBgn0004878	*cas*	6.8777
12	NA	FBgn0000097	*aop*	6.8657
13	NA	FBgn0000216	*Brd*	6.8473
14	NA	FBgn0002629	*m4*	6.7890
15	NA	FBgn0004867	*sop*	6.7395
16	NA	FBgn0014857	*His3.3A*	6.7336
17	NA	FBgn0010410	*RpL27A*	6.5147
18	NA	FBgn0005655	*mus209*	6.4904
19	NA	FBgn0015625	*CycB3*	6.4639
20	NA	FBgn0003887	*βTub56D*	6.3336
21	NA	FBgn0003498	*sqd*	6.2360
22	NA	FBgn0024939	*RpL8*	6.2005
23	NA	FBgn0003941	*RpL40*	6.1453
24	NA	FBgn0003884	*αTub84B*	6.1352

continued on next page...

Table 12 continued from previous page.				
Prediction Rank	Supported with GO:Biological Process annotation	Gene ID	Gene name	Confidence Score (distance from the hyperplane)
25	NA	FBgn0020633	*Mcm7*	6.0494
26	NA	FBgn0002633	*HLHm7*	5.9549
27	NA	FBgn0021776	*mira*	5.9171
28	NA	FBgn0001139	*gro*	5.6689
29	NA	FBgn0004237	*Hrb87F*	5.5946
30	yes	FBgn0003411	*sisA*	5.5579
31	NA	FBgn0003942	*RpS27A*	5.5111
32	NA	FBgn0002638	*Bj1*	5.4976
33	NA	FBgn0011584	*Trp1*	5.3630
34	NA	FBgn0002413	*dco*	5.0303
35	NA	FBgn0020615	*SelD*	5.0220
36	NA	FBgn0002732	$m\alpha$	4.9196
37	NA	FBgn0020496	*CtBP*	4.8394
38	NA	FBgn0002921	$Atp\alpha$	4.8299
39	NA	FBgn0004378	*Klp61F*	4.8208
40	NA	FBgn0001281	*janB*	4.7170
41	NA	FBgn0003719	*tld*	4.6614
42	NA	FBgn0015380	*drl*	4.6467
43	yes	FBgn0000591	*E(spl)*	4.6306
44	NA	FBgn0015622	*Cnx99A*	4.5528
45	NA	FBgn0014861	*Mcm2*	4.5094
46	NA	FBgn0004907	$14\text{-}3\text{-}3\zeta$	4.4646
47	NA	FBgn0010433	*ato*	4.3922
48	NA	FBgn0015805	*Rpd3*	4.3803
49	NA	FBgn0003691	*th*	4.3800
50	NA	FBgn0013272	*Gp150*	4.3479
51	NA	FBgn0000046	*Act87E*	4.3477
52	NA	FBgn0011715	*Snr1*	4.2299
53	NA	FBgn0004828	*His3.3B*	4.1680
54	NA	FBgn0013469	*klu*	4.1622
55	NA	FBgn0000413	*da*	4.1576
56	NA	FBgn0004636	*R*	4.0478
57	NA	FBgn0004106	*cdc2*	4.0410
58	NA	FBgn0024923	*TER94*	3.9968
59	NA	FBgn0003124	*polo*	3.9956
60	NA	FBgn0004595	*pros*	3.9105
61	NA	FBgn0011604	*Iswi*	3.8957
62	NA	FBgn0003525	*stg*	3.8165
63	NA	FBgn0015550	*tap*	3.7878
64	NA	FBgn0024332	*Mcm3*	3.7866
65	NA	FBgn0004177	*mts*	3.7673
66	NA	FBgn0015229	*glec*	3.7168
67	NA	FBgn0001257	*ImpL2*	3.6398
68	NA	FBgn0010278	*Ssrp*	3.5924
69	NA	FBgn0013753	*Bgb*	3.5870
70	NA	FBgn0004493	$DNApol\text{-}\alpha 18$	3.5407
				continued on next page...

Table 12 continued from previous page.

Prediction Rank	Supported with GO:Biological Process annotation	Gene ID	Gene name	Confidence Score (distance from the hyperplane)
71	NA	FBgn0000497	*ds*	3.5136
72	NA	FBgn0004647	*N*	3.5050
73	NA	FBgn0003512	*Sry-δ*	3.5018
74	NA	FBgn0004551	*Ca-P60A*	3.4836
75	NA	FBgn0002914	*Myb*	3.4300
76	NA	FBgn0020493	*Dad*	3.4174
77	NA	FBgn0010300	*brat*	3.3966
78	NA	FBgn0038224	*CG3321*	3.3749
79	NA	FBgn0013725	*phyl*	3.3490
80	NA	FBgn0001104	*G-iα65A*	3.3225
81	NA	FBgn0005672	*spi*	3.2968
82	NA	FBgn0002069	*Aats-asp*	3.2846
83	NA	FBgn0011598	*grp*	3.2834
84	NA	FBgn0015954	*kuz*	3.2575
85	NA	FBgn0000229	*bsk*	3.2497
86	NA	FBgn0011817	*nmo*	3.2496
87	NA	FBgn0015903	*apt*	3.2448
88	NA	FBgn0000411	*D*	3.2290
89	NA	FBgn0025678	*CaBP1*	3.1524
90	NA	FBgn0023441	*fus*	3.1465
91	NA	FBgn0024227	*ial*	3.1337
92	NA	FBgn0003310	*S*	3.1335
93	yes	FBgn0003118	*pnt*	3.1193
94	NA	FBgn0014388	*sty*	3.1125
95	NA	FBgn0010105	*comm*	3.1119
96	NA	FBgn0003471	*β-Spec*	3.0846
97	NA	FBgn0004179	*Csp*	3.0249
98	NA	FBgn0002970	*nub*	3.0199
99	NA	FBgn0001983	*wor*	3.0164
100	NA	FBgn0004364	*18w*	2.9854
101	NA	FBgn0032408	*CG6712*	2.9555
102	NA	FBgn0005642	*wdn*	2.9525
103	NA	FBgn0001180	*hb*	2.9436
104	NA	FBgn0000212	*brm*	2.9212
105	NA	FBgn0004880	*scrt*	2.8824
106	NA	FBgn0000180	*bib*	2.8742
107	NA	FBgn0010110	*east*	2.8660
108	NA	FBgn0004107	*cdc2c*	2.8652
109	NA	FBgn0003886	*αTub85E*	2.8649
110	NA	FBgn0001105	*Gβ13F*	2.8620
111	yes	FBgn0010333	*Rac1*	2.8592
112	yes	FBgn0001148	*gsb*	2.8323
113	NA	FBgn0002989	*okr*	2.8061
114	yes	FBgn0011763	*Dp*	2.8045
115	NA	FBgn0016917	*Stat92E*	2.8001
116	NA	FBgn0002736	*mago*	2.7870

continued on next page...

Table 12 continued from previous page.

Prediction Rank	Supported with GO:Biological Process annotation	Gene ID	Gene name	Confidence Score (distance from the hyperplane)
117	NA	FBgn0022073	*Thor*	2.7854
118	NA	FBgn0002715	*mei-S332*	2.7754
119	NA	FBgn0028554	*xl6*	2.7697
120	NA	FBgn0013269	*FK506-bp1*	2.7691
121	NA	FBgn0003302	*rux*	2.7409

References

[1] Drysdale RA, Crosby MA (2005) FlyBase: genes and gene models. Nucleic Acids Res 33:D390–5.
