# Supplementary material for: Prediction of Gene Expression in Embryonic Structures of Drosophila melanogaster
Source: PLoS Comput Biol. 2007 Jul 20;3(7):e144. doi: 10.1371/journal.pcbi.0030144 (PMC1924873; doi:10.1371/journal.pcbi.0030144)
Supplement: Supplementary file 13 [file pcbi.0030144.st013.pdf]

**Set of Genes**	**Functional units**		
	**pep2ebrain and aep2egut**	**aep2egut and vna2lcord**	**vna2lcord and pep2ebrain**
Training Set	**2 genes** [5% and 3.7%]	**1 gene** [1.8% and 2.9%]	**17 genes** [50% and 42.5%]
Top scored 30% of *de novo* predictions	**52 genes** [35.6% and 42.9%]	**17 genes** [14% and 13.6%]	**27 genes** [21.6% and 18.5%]

Table 13: Number of genes simultaneously attributed to two functional units encompassing developmental processes in the late embryogenesis. Genes shared between functional units are displayed in each column. Figures expressed as a percentage show a fraction of shared genes relative to the total number of genes associated with a functional unit. An arrangement of percentage figures reflects the order in which functional units are listed in the top row of the table.
