# Supplementary material for: Prediction of Gene Expression in Embryonic Structures of Drosophila melanogaster
Source: PLoS Comput Biol. 2007 Jul 20;3(7):e144. doi: 10.1371/journal.pcbi.0030144 (PMC1924873; doi:10.1371/journal.pcbi.0030144)
Supplement: Supplementary file 14 [file pcbi.0030144.st014.pdf]

Table 14: **Diversity of anatomical annotations of genes comprising functional unit mat2pep**: procephalic ectoderm primordium && cellular blastoderm && maternal

Anatomical Structures	Genes
ventral ectoderm anlage;	
	FBgn0037878 CG6693 CG6693 LD05521
	FBgn0010342 Map60 CG1825 LD02709
	FBgn0036518 RhoGAP71E CG32149 LD04071
	FBgn0036494 Toll-6 CG7250 LD08841
	FBgn0011760 ctp CG6998 LD24056
	FBgn0039537 CG5590 CG5590 GH01709
	FBgn0028554 xl6 CG10203 LD46359
	FBgn0002932 neur CG11988 LD45505
	FBgn0039338 XNP CG4548 SD07188
	FBgn0052066 CG32066 CG32066 GH09096
	FBgn0037207 CG11100 CG11100 SD09884
	FBgn0053097 CG33097 CG33097 GH12404
	FBgn0031493 CG3605 CG3605 LD45152
	FBgn0015903 apt CG5393 LD45581
	FBgn0030468 CG1622 CG1622 GM12605
	FBgn0052956 CG32956 CG32956 RE06926
	FBgn0040230 dbo CG6224 RE13447
	FBgn0001086 fzy CG4274 RE29723
	FBgn0033749 achintya CG8819 RE51781
	FBgn0032847 Taf18 CG10756 RE56212
	FBgn0011638 La CG10922 RE69138
	FBgn0011715 Snr1 CG1064 GH08712
	FBgn0019637 Atu CG1433 LD30655
	FBgn0032130 CG3838 CG3838 LD21447
	FBgn0015903 apt CG5393 LD45581
	FBgn0011766 E2f CG6376 GH16721
	FBgn0015930 dpld CG1624 LD39167
	FBgn0000611 exd CG8933 LD03509
	FBgn0038192 CG9924 CG9924 GM07940
	FBgn0029867 CG3847 CG3847 GM08802
	FBgn0003346 Sd CG9999 LD16356
	FBgn0038476 CG5175 CG5175 LD09231
	FBgn0037066 CG7752 CG7752 LD15904
	FBgn0010300 brat CG10719 LD16270
	FBgn0038222 CG3307 CG3307 LD12042
	FBgn0052434 CG32434 CG32434 RE02556
	FBgn0038118 NULL CG7855 RE03551
mesoderm anlage in statu nascendi;	
	FBgn0000611 exd CG8933 LD03509
	FBgn0003346 Sd CG9999 LD16356
gap;	
	FBgn0040271 Sulf1 CG6725 SD04414
	continued on next page...

Table 14 continued from previous page.	
Anatomical Structures	Genes
visual anlage in statu nascendi;	
	FBgn0000611 exd CG8933 LD03509<br>FBgn0003346 Sd CG9999 LD16356
segmentally repeated;	
	FBgn0032130 CG3838 CG3838 LD21447
pole cell;	
	FBgn0039211 CG13620 CG13620 SD03914<br>FBgn0046687 Tre1 CG3171 RE07751
antennal primordium2;	
	FBgn0003346 Sd CG9999 LD16356
head mesoderm anlage;	
	FBgn0024332 Mcm3 CG4206 LD07144<br>FBgn0010226 GstS1 CG8938 LD21131<br>FBgn0037707 RnpS1 CG16788 LD23870<br>FBgn0039537 CG5590 CG5590 GH01709<br>FBgn0002932 neur CG11988 LD45505<br>FBgn0035205 CG2469 CG2469 LD24034<br>FBgn0052956 CG32956 CG32956 RE06926<br>FBgn0032847 Taf18 CG10756 RE56212<br>FBgn0011638 La CG10922 RE69138<br>FBgn0011715 Snr1 CG1064 GH08712<br>FBgn0032130 CG3838 CG3838 LD21447<br>FBgn0051365 CG31365 CG31365 LD28458<br>FBgn0000611 exd CG8933 LD03509<br>FBgn0003346 Sd CG9999 LD16356<br>FBgn0027620 Acf1 CG1966 LD09043<br>FBgn0038476 CG5175 CG5175 LD09231<br>FBgn0038222 CG3307 CG3307 LD12042
hindgut anlage in statu nascendi;	
	FBgn0040230 dbo CG6224 RE13447<br>FBgn0000611 exd CG8933 LD03509<br>FBgn0003346 Sd CG9999 LD16356
procephalic ectoderm primordium;	
	FBgn0037878 CG6693 CG6693 LD05521<br>NULL NULL NULL LD14406<br>FBgn0004378 Klp61F CG9191 LD15641<br>FBgn0010342 Map60 CG1825 LD02709<br>FBgn0036518 RhoGAP71E CG32149 LD04071<br>FBgn0027783 SMC2 CG10212 LD05471<br>FBgn0024332 Mcm3 CG4206 LD07144<br>FBgn0036494 Toll-6 CG7250 LD08841<br>FBgn0003346 Sd CG9999 LD16356<br>FBgn0003520 stau CG5753 LD13210<br>FBgn0020255 ran CG1404 GM14354<br>FBgn0010226 GstS1 CG8938 LD21131<br>FBgn0037707 RnpS1 CG16788 LD23870
	continued on next page...

Table 14 continued from previous page.

Anatomical Structures	Genes
	FBgn0011760 ctp CG6998 LD24056<br>FBgn0029629 CG8636 CG8636 LD24347<br>FBgn0039537 CG5590 CG5590 GH01709<br>FBgn0003862 trx CG8651 LD39445<br>FBgn0028554 xl6 CG10203 LD46359<br>FBgn0004868 Gdi CG4422 LD46767<br>FBgn0039250 CG11120 CG11120 LD43724<br>FBgn0002932 neur CG11988 LD45505<br>FBgn0039338 XNP CG4548 SD07188<br>FBgn0052066 CG32066 CG32066 GH09096<br>FBgn0037207 CG11100 CG11100 SD09884<br>FBgn0053097 CG33097 CG33097 GH12404<br>FBgn0031493 CG3605 CG3605 LD45152<br>FBgn0015903 apt CG5393 LD45581<br>FBgn0030468 CG1622 CG1622 GM12605<br>FBgn0040271 Sulf1 CG6725 SD04414<br>FBgn0035205 CG2469 CG2469 LD24034<br>FBgn0027948 msps CG5000 GH18634<br>FBgn0052956 CG32956 CG32956 RE06926<br>FBgn0040230 dbo CG6224 RE13447<br>FBgn0036973 CG5585 CG5585 RE17089<br>FBgn0001086 fzy CG4274 RE29723<br>FBgn0013347 TfIIA-S CG5163 RE44302<br>FBgn0033749 achintya CG8819 RE51781<br>FBgn0032847 Taf18 CG10756 RE56212<br>FBgn0011638 La CG10922 RE69138<br>FBgn0017550 Rga CG2161 GM14102<br>FBgn0015396 jumu CG4029 LD24749<br>FBgn0011715 Snr1 CG1064 GH08712<br>FBgn0022720 zf30C CG3998 LD23102<br>FBgn0019637 Atu CG1433 LD30655<br>FBgn0039350 CG17383 CG17383 LD33329<br>FBgn0032130 CG3838 CG3838 LD21447<br>FBgn0051365 CG31365 CG31365 LD28458<br>FBgn0015903 apt CG5393 LD45581<br>FBgn0011766 E2f CG6376 GH16721<br>FBgn0005642 wdn CG1454 LD40824<br>FBgn0039211 CG13620 CG13620 SD03914<br>FBgn0015930 dpld CG1624 LD39167<br>FBgn0000611 exd CG8933 LD03509<br>FBgn0029903 CG4532 CG4532 LD08951<br>FBgn0038192 CG9924 CG9924 GM07940<br>FBgn0029867 CG3847 CG3847 GM08802<br>FBgn0003346 Sd CG9999 LD16356<br>FBgn0027620 Acf1 CG1966 LD09043<br>FBgn0038476 CG5175 CG5175 LD09231

continued on next page...

Table 14 continued from previous page.

Anatomical Structures	Genes
	FBgn0037066 CG7752 CG7752 LD15904
	FBgn0010300 brat CG10719 LD16270
	FBgn0038222 CG3307 CG3307 LD12042
	FBgn0052434 CG32434 CG32434 RE02556
	FBgn0038118 NULL CG7855 RE03551
	FBgn0046687 Tre1 CG3171 RE07751
	FBgn0031878 CG9188 CG9188 RE16905
	FBgn0045064 bwa CG13969 RE26924
	FBgn0032919 CG9253 CG9253 RE27528
	FBgn0029798 CG4078 CG4078 RE31401
	FBgn0030507 CG11164 CG11164 RE50056
	FBgn0037770 CG5358 CG5358 RE68504
	FBgn0029512 Aos1 CG12276 LD33652
subset;	
	FBgn0036494 Toll-6 CG7250 LD08841
	FBgn0037207 CG11100 CG11100 SD09884
	FBgn0015903 apt CG5393 LD45581
	FBgn0040271 Sulf1 CG6725 SD04414
	FBgn0027948 msps CG5000 GH18634
	FBgn0040230 dbo CG6224 RE13447
	FBgn0015396 jumu CG4029 LD24749
	FBgn0039350 CG17383 CG17383 LD33329
	FBgn0015903 apt CG5393 LD45581
	FBgn0011766 E2f CG6376 GH16721
	FBgn0039211 CG13620 CG13620 SD03914
	FBgn0038192 CG9924 CG9924 GM07940
	FBgn0010300 brat CG10719 LD16270
	FBgn0052434 CG32434 CG32434 RE02556
	FBgn0045064 bwa CG13969 RE26924
external foregut primordium;	
	FBgn0029629 CG8636 CG8636 LD24347
	FBgn0015903 apt CG5393 LD45581
	FBgn0038192 CG9924 CG9924 GM07940
	FBgn0003346 Sd CG9999 LD16356
dorsal ectoderm primordium;	
	FBgn0036494 Toll-6 CG7250 LD08841
	FBgn0015903 apt CG5393 LD45581
	FBgn0040271 Sulf1 CG6725 SD04414
	FBgn0032847 Taf18 CG10756 RE56212
	FBgn0015396 jumu CG4029 LD24749
	FBgn0015903 apt CG5393 LD45581
	FBgn0000611 exd CG8933 LD03509
	FBgn0003346 Sd CG9999 LD16356
	FBgn0052434 CG32434 CG32434 RE02556
endoderm anlage in statu nascendi;	
	FBgn0003346 Sd CG9999 LD16356

continued on next page...

Table 14 continued from previous page.	
Anatomical Structures	Genes
head epidermis primordium;	
	FBgn0015903 apt CG5393 LD45581
	FBgn0015903 apt CG5393 LD45581
	FBgn0003346 Sd CG9999 LD16356
ventral neuroderm anlage;	
	FBgn0036494 Toll-6 CG7250 LD08841
	FBgn0037707 RnpS1 CG16788 LD23870
	FBgn0002932 neur CG11988 LD45505
	FBgn0040271 Sulf1 CG6725 SD04414
	FBgn0040230 dbo CG6224 RE13447
	FBgn0032130 CG3838 CG3838 LD21447
	FBgn0015903 apt CG5393 LD45581
	FBgn0000611 exd CG8933 LD03509
	FBgn0003346 Sd CG9999 LD16356
	FBgn0038118 NULL CG7855 RE03551
	FBgn0046687 Tre1 CG3171 RE07751
	FBgn0031878 CG9188 CG9188 RE16905
	FBgn0030507 CG11164 CG11164 RE50056
	FBgn0037770 CG5358 CG5358 RE68504
	FBgn0029512 Aos1 CG12276 LD33652
trunk mesoderm anlage;	
	FBgn0024332 Mcm3 CG4206 LD07144
	FBgn0010226 GstS1 CG8938 LD21131
	FBgn0037707 RnpS1 CG16788 LD23870
	FBgn0039537 CG5590 CG5590 GH01709
	FBgn0003862 trx CG8651 LD39445
	FBgn0002932 neur CG11988 LD45505
	FBgn0052066 CG32066 CG32066 GH09096
	FBgn0037207 CG11100 CG11100 SD09884
	FBgn0031493 CG3605 CG3605 LD45152
	FBgn0035205 CG2469 CG2469 LD24034
	FBgn0052956 CG32956 CG32956 RE06926
	FBgn0032847 Taf18 CG10756 RE56212
	FBgn0011638 La CG10922 RE69138
	FBgn0011715 Snr1 CG1064 GH08712
	FBgn0032130 CG3838 CG3838 LD21447
	FBgn0051365 CG31365 CG31365 LD28458
	FBgn0000611 exd CG8933 LD03509
	FBgn0003346 Sd CG9999 LD16356
	FBgn0027620 Acf1 CG1966 LD09043
	FBgn0038476 CG5175 CG5175 LD09231
	FBgn0038222 CG3307 CG3307 LD12042
anterior endoderm anlage;	
	FBgn0036518 RhoGAP71E CG32149 LD04071
	FBgn0010226 GstS1 CG8938 LD21131
	FBgn0037707 RnpS1 CG16788 LD23870

continued on next page...

Table 14 continued from previous page.

Anatomical Structures	Genes
	FBgn0029629 CG8636 CG8636 LD24347
	FBgn0039537 CG5590 CG5590 GH01709
	FBgn0002932 neur CG11988 LD45505
	FBgn0053097 CG33097 CG33097 GH12404
	FBgn0031493 CG3605 CG3605 LD45152
	FBgn0032847 Taf18 CG10756 RE56212
	FBgn0011638 La CG10922 RE69138
	FBgn0032130 CG3838 CG3838 LD21447
	FBgn0051365 CG31365 CG31365 LD28458
	FBgn0015903 apt CG5393 LD45581
	FBgn0000611 exd CG8933 LD03509
	FBgn0003346 Sd CG9999 LD16356
	FBgn0038476 CG5175 CG5175 LD09231
	FBgn0038222 CG3307 CG3307 LD12042
	FBgn0037770 CG5358 CG5358 RE68504
ventral nerve cord primordium;	
	FBgn0037878 CG6693 CG6693 LD05521
	NULL NULL NULL LD14406
	FBgn0004378 Klp61F CG9191 LD15641
	FBgn0010342 Map60 CG1825 LD02709
	FBgn0036518 RhoGAP71E CG32149 LD04071
	FBgn0027783 SMC2 CG10212 LD05471
	FBgn0024332 Mcm3 CG4206 LD07144
	FBgn0036494 Toll-6 CG7250 LD08841
	FBgn0003346 Sd CG9999 LD16356
	FBgn0003520 stau CG5753 LD13210
	FBgn0020255 ran CG1404 GM14354
	FBgn0037707 RnpS1 CG16788 LD23870
	FBgn0039537 CG5590 CG5590 GH01709
	FBgn0003862 trx CG8651 LD39445
	FBgn0028554 xl6 CG10203 LD46359
	FBgn0004868 Gdi CG4422 LD46767
	FBgn0039250 CG11120 CG11120 LD43724
	FBgn0002932 neur CG11988 LD45505
	FBgn0039338 XNP CG4548 SD07188
	FBgn0052066 CG32066 CG32066 GH09096
	FBgn0053097 CG33097 CG33097 GH12404
	FBgn0031493 CG3605 CG3605 LD45152
	FBgn0030468 CG1622 CG1622 GM12605
	FBgn0040271 Sulf1 CG6725 SD04414
	FBgn0035205 CG2469 CG2469 LD24034
	FBgn0027948 msps CG5000 GH18634
	FBgn0040230 dbo CG6224 RE13447
	FBgn0036973 CG5585 CG5585 RE17089
	FBgn0013347 TfIIA-S CG5163 RE44302
	FBgn0033749 achintya CG8819 RE51781

continued on next page...

Table 14 continued from previous page.	
Anatomical Structures	Genes
	FBgn0017550 Rga CG2161 GM14102<br>FBgn0015396 jumu CG4029 LD24749<br>FBgn0011715 Snr1 CG1064 GH08712<br>FBgn0019637 Atu CG1433 LD30655<br>FBgn0032130 CG3838 CG3838 LD21447<br>FBgn0051365 CG31365 CG31365 LD28458<br>FBgn0015903 apt CG5393 LD45581<br>FBgn0011766 E2f CG6376 GH16721<br>FBgn0005642 wdn CG1454 LD40824<br>FBgn0015930 dpld CG1624 LD39167<br>FBgn0000611 exd CG8933 LD03509<br>FBgn0029903 CG4532 CG4532 LD08951<br>FBgn0038192 CG9924 CG9924 GM07940<br>FBgn0003346 Sd CG9999 LD16356<br>FBgn0027620 Acf1 CG1966 LD09043<br>FBgn0038476 CG5175 CG5175 LD09231<br>FBgn0010300 brat CG10719 LD16270<br>FBgn0038222 CG3307 CG3307 LD12042<br>FBgn0052434 CG32434 CG32434 RE02556<br>FBgn0038118 NULL CG7855 RE03551<br>FBgn0046687 Tre1 CG3171 RE07751<br>FBgn0031878 CG9188 CG9188 RE16905<br>FBgn0029798 CG4078 CG4078 RE31401<br>FBgn0030507 CG11164 CG11164 RE50056<br>FBgn0029512 Aos1 CG12276 LD33652
visual primordium;	
	FBgn0002932 neur CG11988 LD45505<br>FBgn0015903 apt CG5393 LD45581<br>FBgn0000611 exd CG8933 LD03509<br>FBgn0003346 Sd CG9999 LD16356<br>FBgn0052434 CG32434 CG32434 RE02556
foregut anlage in statu nascendi;	
	FBgn0000611 exd CG8933 LD03509<br>FBgn0003346 Sd CG9999 LD16356
procephalic ectoderm anlage;	
	FBgn0037878 CG6693 CG6693 LD05521<br>FBgn0010342 Map60 CG1825 LD02709<br>FBgn0036494 Toll-6 CG7250 LD08841<br>FBgn0037707 RnpS1 CG16788 LD23870<br>FBgn0011760 ctp CG6998 LD24056<br>FBgn0028554 xl6 CG10203 LD46359<br>FBgn0002932 neur CG11988 LD45505<br>FBgn0039338 XNP CG4548 SD07188<br>FBgn0053097 CG33097 CG33097 GH12404<br>FBgn0015903 apt CG5393 LD45581<br>FBgn0030468 CG1622 CG1622 GM12605

continued on next page...

Table 14 continued from previous page.	
Anatomical Structures	Genes
	FBgn0052956 CG32956 CG32956 RE06926<br>FBgn0040230 dbo CG6224 RE13447<br>FBgn0001086 fzy CG4274 RE29723<br>FBgn0033749 achintya CG8819 RE51781<br>FBgn0032847 Taf18 CG10756 RE56212<br>FBgn0011638 La CG10922 RE69138<br>FBgn0015903 apt CG5393 LD45581<br>FBgn0011766 E2f CG6376 GH16721<br>FBgn0039211 CG13620 CG13620 SD03914<br>FBgn0015930 dpld CG1624 LD39167<br>FBgn0000611 exd CG8933 LD03509<br>FBgn0038192 CG9924 CG9924 GM07940<br>FBgn0029867 CG3847 CG3847 GM08802<br>FBgn0003346 Sd CG9999 LD16356<br>FBgn0038476 CG5175 CG5175 LD09231<br>FBgn0037066 CG7752 CG7752 LD15904<br>FBgn0010300 brat CG10719 LD16270<br>FBgn0052434 CG32434 CG32434 RE02556<br>FBgn0038118 NULL CG7855 RE03551<br>FBgn0046687 Tre1 CG3171 RE07751<br>FBgn0031878 CG9188 CG9188 RE16905<br>FBgn0030507 CG11164 CG11164 RE50056<br>FBgn0029512 Aos1 CG12276 LD33652
dorsal ectoderm anlage;	
	FBgn0015930 dpld CG1624 LD39167<br>FBgn0000611 exd CG8933 LD03509<br>FBgn0003346 Sd CG9999 LD16356
mesectoderm primordium;	
	FBgn0002932 neur CG11988 LD45505<br>FBgn0003346 Sd CG9999 LD16356
anlage in statu nascendi;	
	FBgn0036494 Toll-6 CG7250 LD08841<br>FBgn0040230 dbo CG6224 RE13447<br>FBgn0000611 exd CG8933 LD03509<br>FBgn0003346 Sd CG9999 LD16356<br>FBgn0045064 bwa CG13969 RE26924
posterior endoderm anlage;	
	FBgn0036518 RhoGAP71E CG32149 LD04071<br>FBgn0010226 GstS1 CG8938 LD21131<br>FBgn0037707 RnpS1 CG16788 LD23870<br>FBgn0029629 CG8636 CG8636 LD24347<br>FBgn0039537 CG5590 CG5590 GH01709<br>FBgn0002932 neur CG11988 LD45505<br>FBgn0039338 XNP CG4548 SD07188<br>FBgn0053097 CG33097 CG33097 GH12404<br>FBgn0031493 CG3605 CG3605 LD45152
	continued on next page...

Table 14 continued from previous page.	
Anatomical Structures	Genes
maternal;	FBgn0015903 apt CG5393 LD45581<br>FBgn0052956 CG32956 CG32956 RE06926<br>FBgn0032847 Taf18 CG10756 RE56212<br>FBgn0011638 La CG10922 RE69138<br>FBgn0011715 Snr1 CG1064 GH08712<br>FBgn0019637 Atu CG1433 LD30655<br>FBgn0032130 CG3838 CG3838 LD21447<br>FBgn0051365 CG31365 CG31365 LD28458<br>FBgn0015903 apt CG5393 LD45581<br>FBgn0000611 exd CG8933 LD03509<br>FBgn0003346 Sd CG9999 LD16356<br>FBgn0038222 CG3307 CG3307 LD12042<br>FBgn0037770 CG5358 CG5358 RE68504<br><br>FBgn0037878 CG6693 CG6693 LD05521<br>NULL NULL NULL LD14406<br>FBgn0004378 Klp61F CG9191 LD15641<br>FBgn0010342 Map60 CG1825 LD02709<br>FBgn0036518 RhoGAP71E CG32149 LD04071<br>FBgn0027783 SMC2 CG10212 LD05471<br>FBgn0024332 Mcm3 CG4206 LD07144<br>FBgn0036494 Toll-6 CG7250 LD08841<br>FBgn0003346 Sd CG9999 LD16356<br>FBgn0003520 stau CG5753 LD13210<br>FBgn0020255 ran CG1404 GM14354<br>FBgn0010226 GstS1 CG8938 LD21131<br>FBgn0037707 RnpS1 CG16788 LD23870<br>FBgn0011760 ctp CG6998 LD24056<br>FBgn0029629 CG8636 CG8636 LD24347<br>FBgn0039537 CG5590 CG5590 GH01709<br>FBgn0003862 trx CG8651 LD39445<br>FBgn0028554 xl6 CG10203 LD46359<br>FBgn0004868 Gdi CG4422 LD46767<br>FBgn0039250 CG11120 CG11120 LD43724<br>FBgn0002932 neur CG11988 LD45505<br>FBgn0039338 XNP CG4548 SD07188<br>FBgn0052066 CG32066 CG32066 GH09096<br>FBgn0037207 CG11100 CG11100 SD09884<br>FBgn0053097 CG33097 CG33097 GH12404<br>FBgn0031493 CG3605 CG3605 LD45152<br>FBgn0015903 apt CG5393 LD45581<br>FBgn0030468 CG1622 CG1622 GM12605<br>FBgn0040271 Sulf1 CG6725 SD04414<br>FBgn0035205 CG2469 CG2469 LD24034<br>FBgn0027948 msps CG5000 GH18634<br>FBgn0052956 CG32956 CG32956 RE06926
	continued on next page...

Table 14 continued from previous page.

Anatomical Structures	Genes
	FBgn0040230 dbo CG6224 RE13447
	FBgn0036973 CG5585 CG5585 RE17089
	FBgn0001086 fzy CG4274 RE29723
	FBgn0013347 TfIIA-S CG5163 RE44302
	FBgn0033749 achintya CG8819 RE51781
	FBgn0032847 Taf18 CG10756 RE56212
	FBgn0011638 La CG10922 RE69138
	FBgn0017550 Rga CG2161 GM14102
	FBgn0015396 jumu CG4029 LD24749
	FBgn0011715 Snr1 CG1064 GH08712
	FBgn0022720 zf30C CG3998 LD23102
	FBgn0019637 Atu CG1433 LD30655
	FBgn0039350 CG17383 CG17383 LD33329
	FBgn0032130 CG3838 CG3838 LD21447
	FBgn0051365 CG31365 CG31365 LD28458
	FBgn0015903 apt CG5393 LD45581
	FBgn0011766 E2f CG6376 GH16721
	FBgn0005642 wdn CG1454 LD40824
	FBgn0039211 CG13620 CG13620 SD03914
	FBgn0015930 dpld CG1624 LD39167
	FBgn0000611 exd CG8933 LD03509
	FBgn0029903 CG4532 CG4532 LD08951
	FBgn0038192 CG9924 CG9924 GM07940
	FBgn0029867 CG3847 CG3847 GM08802
	FBgn0003346 Sd CG9999 LD16356
	FBgn0027620 Acf1 CG1966 LD09043
	FBgn0038476 CG5175 CG5175 LD09231
	FBgn0037066 CG7752 CG7752 LD15904
	FBgn0010300 brat CG10719 LD16270
	FBgn0038222 CG3307 CG3307 LD12042
	FBgn0052434 CG32434 CG32434 RE02556
	FBgn0038118 NULL CG7855 RE03551
	FBgn0046687 Tre1 CG3171 RE07751
	FBgn0031878 CG9188 CG9188 RE16905
	FBgn0045064 bwa CG13969 RE26924
	FBgn0032919 CG9253 CG9253 RE27528
	FBgn0029798 CG4078 CG4078 RE31401
	FBgn0030507 CG11164 CG11164 RE50056
	FBgn0037770 CG5358 CG5358 RE68504
	FBgn0029512 Aos1 CG12276 LD33652
mesectoderm anlage;	
	FBgn0037707 RnpS1 CG16788 LD23870
	FBgn0002932 neur CG11988 LD45505
	FBgn0037207 CG11100 CG11100 SD09884
	FBgn0000611 exd CG8933 LD03509
	FBgn0003346 Sd CG9999 LD16356

continued on next page...

Table 14 continued from previous page.	
Anatomical Structures	Genes
mesectoderm anlage in statu nascendi;	
	FBgn0000611 exd CG8933 LD03509<br>FBgn0003346 Sd CG9999 LD16356
amnioserosa;	
	FBgn0046687 Tre1 CG3171 RE07751<br>FBgn0045064 bwa CG13969 RE26924
trunk mesoderm primordium;	
	NULL NULL NULL LD14406<br>FBgn0037707 RnpS1 CG16788 LD23870<br>FBgn0039537 CG5590 CG5590 GH01709<br>FBgn0003862 trx CG8651 LD39445<br>FBgn0052066 CG32066 CG32066 GH09096<br>FBgn0037207 CG11100 CG11100 SD09884<br>FBgn0031493 CG3605 CG3605 LD45152<br>FBgn0035205 CG2469 CG2469 LD24034<br>FBgn0052956 CG32956 CG32956 RE06926<br>FBgn0032847 Taf18 CG10756 RE56212<br>FBgn0011638 La CG10922 RE69138<br>FBgn0011715 Snr1 CG1064 GH08712<br>FBgn0051365 CG31365 CG31365 LD28458<br>FBgn0000611 exd CG8933 LD03509<br>FBgn0003346 Sd CG9999 LD16356<br>FBgn0038476 CG5175 CG5175 LD09231<br>FBgn0038222 CG3307 CG3307 LD12042<br>FBgn0032919 CG9253 CG9253 RE27528
pole plasm;	
	FBgn0046687 Tre1 CG3171 RE07751
ventral ectoderm primordium;	
	FBgn0036494 Toll-6 CG7250 LD08841<br>FBgn0003520 stau CG5753 LD13210<br>FBgn0031493 CG3605 CG3605 LD45152<br>FBgn0015903 apt CG5393 LD45581<br>FBgn0040230 dbo CG6224 RE13447<br>FBgn0011638 La CG10922 RE69138<br>FBgn0015396 jumu CG4029 LD24749<br>FBgn0032130 CG3838 CG3838 LD21447<br>FBgn0015903 apt CG5393 LD45581<br>FBgn0000611 exd CG8933 LD03509<br>FBgn0003346 Sd CG9999 LD16356<br>FBgn0027620 Acf1 CG1966 LD09043<br>FBgn0045064 bwa CG13969 RE26924<br>FBgn0030507 CG11164 CG11164 RE50056
visual anlage;	
	FBgn0015903 apt CG5393 LD45581<br>FBgn0000611 exd CG8933 LD03509<br>FBgn0003346 Sd CG9999 LD16356
	continued on next page...

Table 14 continued from previous page.	
Anatomical Structures	Genes
procephalic ectoderm anlage in statu nascendi;	
	FBgn0036494 Toll-6 CG7250 LD08841<br>FBgn0040230 dbo CG6224 RE13447<br>FBgn0046687 Tre1 CG3171 RE07751
ectoderm anlage in statu nascendi;	
	FBgn0036494 Toll-6 CG7250 LD08841<br>FBgn0040230 dbo CG6224 RE13447<br>FBgn0003346 Sd CG9999 LD16356<br>FBgn0045064 bwa CG13969 RE26924
inclusive hindgut primordium;	
	NULL NULL NULL LD14406<br>FBgn0037707 RnpS1 CG16788 LD23870<br>FBgn0011760 ctp CG6998 LD24056<br>FBgn0029629 CG8636 CG8636 LD24347<br>FBgn0004868 Gdi CG4422 LD46767<br>FBgn0053097 CG33097 CG33097 GH12404<br>FBgn0031493 CG3605 CG3605 LD45152<br>FBgn0015903 apt CG5393 LD45581<br>FBgn0040230 dbo CG6224 RE13447<br>FBgn0011638 La CG10922 RE69138<br>FBgn0022720 zf30C CG3998 LD23102<br>FBgn0015903 apt CG5393 LD45581<br>FBgn0000611 exd CG8933 LD03509<br>FBgn0038192 CG9924 CG9924 GM07940<br>FBgn0003346 Sd CG9999 LD16356
foregut anlage;	
	FBgn0037707 RnpS1 CG16788 LD23870<br>FBgn0002932 neur CG11988 LD45505<br>FBgn0015903 apt CG5393 LD45581<br>FBgn0000611 exd CG8933 LD03509<br>FBgn0003346 Sd CG9999 LD16356
dorsal ectoderm anlage in statu nascendi;	
	FBgn0000611 exd CG8933 LD03509<br>FBgn0003346 Sd CG9999 LD16356
cellular blastoderm;	
	FBgn0037878 CG6693 CG6693 LD05521<br>NULL NULL NULL LD14406<br>FBgn0004378 Klp61F CG9191 LD15641<br>FBgn0010342 Map60 CG1825 LD02709<br>FBgn0036518 RhoGAP71E CG32149 LD04071<br>FBgn0027783 SMC2 CG10212 LD05471<br>FBgn0024332 Mcm3 CG4206 LD07144<br>FBgn0036494 Toll-6 CG7250 LD08841<br>FBgn0003346 Sd CG9999 LD16356<br>FBgn0003520 stau CG5753 LD13210<br>FBgn0020255 ran CG1404 GM14354

continued on next page...

Table 14 continued from previous page.	
Anatomical Structures	Genes
	FBgn0010226 GstS1 CG8938 LD21131<br>FBgn0037707 RnpS1 CG16788 LD23870<br>FBgn0011760 ctp CG6998 LD24056<br>FBgn0029629 CG8636 CG8636 LD24347<br>FBgn0039537 CG5590 CG5590 GH01709<br>FBgn0003862 trx CG8651 LD39445<br>FBgn0028554 xl6 CG10203 LD46359<br>FBgn0004868 Gdi CG4422 LD46767<br>FBgn0039250 CG11120 CG11120 LD43724<br>FBgn0002932 neur CG11988 LD45505<br>FBgn0039338 XNP CG4548 SD07188<br>FBgn0052066 CG32066 CG32066 GH09096<br>FBgn0037207 CG11100 CG11100 SD09884<br>FBgn0053097 CG33097 CG33097 GH12404<br>FBgn0031493 CG3605 CG3605 LD45152<br>FBgn0015903 apt CG5393 LD45581<br>FBgn0030468 CG1622 CG1622 GM12605<br>FBgn0040271 Sulf1 CG6725 SD04414<br>FBgn0035205 CG2469 CG2469 LD24034<br>FBgn0027948 msps CG5000 GH18634<br>FBgn0052956 CG32956 CG32956 RE06926<br>FBgn0040230 dbo CG6224 RE13447<br>FBgn0036973 CG5585 CG5585 RE17089<br>FBgn0001086 fzy CG4274 RE29723<br>FBgn0013347 TfIIA-S CG5163 RE44302<br>FBgn0033749 achintya CG8819 RE51781<br>FBgn0032847 Taf18 CG10756 RE56212<br>FBgn0011638 La CG10922 RE69138<br>FBgn0017550 Rga CG2161 GM14102<br>FBgn0015396 jumu CG4029 LD24749<br>FBgn0011715 Snr1 CG1064 GH08712<br>FBgn0022720 zf30C CG3998 LD23102<br>FBgn0019637 Atu CG1433 LD30655<br>FBgn0039350 CG17383 CG17383 LD33329<br>FBgn0032130 CG3838 CG3838 LD21447<br>FBgn0051365 CG31365 CG31365 LD28458<br>FBgn0015903 apt CG5393 LD45581<br>FBgn0011766 E2f CG6376 GH16721<br>FBgn0005642 wdn CG1454 LD40824<br>FBgn0039211 CG13620 CG13620 SD03914<br>FBgn0015930 dpld CG1624 LD39167<br>FBgn0000611 exd CG8933 LD03509<br>FBgn0029903 CG4532 CG4532 LD08951<br>FBgn0038192 CG9924 CG9924 GM07940<br>FBgn0029867 CG3847 CG3847 GM08802<br>FBgn0003346 Sd CG9999 LD16356
	continued on next page...

Table 14 continued from previous page.

Anatomical Structures	Genes
hindgut anlage;	FBgn0027620 Acf1 CG1966 LD09043<br>FBgn0038476 CG5175 CG5175 LD09231<br>FBgn0037066 CG7752 CG7752 LD15904<br>FBgn0010300 brat CG10719 LD16270<br>FBgn0038222 CG3307 CG3307 LD12042<br>FBgn0052434 CG32434 CG32434 RE02556<br>FBgn0038118 NULL CG7855 RE03551<br>FBgn0046687 Tre1 CG3171 RE07751<br>FBgn0031878 CG9188 CG9188 RE16905<br>FBgn0045064 bwa CG13969 RE26924<br>FBgn0032919 CG9253 CG9253 RE27528<br>FBgn0029798 CG4078 CG4078 RE31401<br>FBgn0030507 CG11164 CG11164 RE50056<br>FBgn0037770 CG5358 CG5358 RE68504<br>FBgn0029512 Aos1 CG12276 LD33652
anterior endoderm primordium;	FBgn0037707 RnpS1 CG16788 LD23870<br>FBgn0011760 ctp CG6998 LD24056<br>FBgn0029629 CG8636 CG8636 LD24347<br>FBgn0053097 CG33097 CG33097 GH12404<br>FBgn0031493 CG3605 CG3605 LD45152<br>FBgn0015903 apt CG5393 LD45581<br>FBgn0040230 dbo CG6224 RE13447<br>FBgn0011638 La CG10922 RE69138<br>FBgn0015903 apt CG5393 LD45581<br>FBgn0000611 exd CG8933 LD03509<br>FBgn0038192 CG9924 CG9924 GM07940<br>FBgn0003346 Sd CG9999 LD16356
head mesoderm P2 primordium;	FBgn0037707 RnpS1 CG16788 LD23870<br>FBgn0029629 CG8636 CG8636 LD24347<br>FBgn0039537 CG5590 CG5590 GH01709<br>FBgn0031493 CG3605 CG3605 LD45152<br>FBgn0052956 CG32956 CG32956 RE06926<br>FBgn0036973 CG5585 CG5585 RE17089<br>FBgn0013347 TfIIA-S CG5163 RE44302<br>FBgn0032847 Taf18 CG10756 RE56212<br>FBgn0011638 La CG10922 RE69138<br>FBgn0011715 Snr1 CG1064 GH08712<br>FBgn0032130 CG3838 CG3838 LD21447<br>FBgn0051365 CG31365 CG31365 LD28458<br>FBgn0015903 apt CG5393 LD45581<br>FBgn0000611 exd CG8933 LD03509<br>FBgn0003346 Sd CG9999 LD16356<br>FBgn0032919 CG9253 CG9253 RE27528

continued on next page...

Table 14 continued from previous page.	
Anatomical Structures	Genes
posterior endoderm primordium;	FBgn0039537 CG5590 CG5590 GH01709<br>FBgn0037207 CG11100 CG11100 SD09884<br>FBgn0032847 Taf18 CG10756 RE56212<br>FBgn0011638 La CG10922 RE69138<br>FBgn0051365 CG31365 CG31365 LD28458<br>FBgn0000611 exd CG8933 LD03509<br>FBgn0003346 Sd CG9999 LD16356<br>FBgn0038222 CG3307 CG3307 LD12042<br>FBgn0032919 CG9253 CG9253 RE27528
ventral ectoderm anlage in statu nascendi;	FBgn0010226 GstS1 CG8938 LD21131<br>FBgn0037707 RnpS1 CG16788 LD23870<br>FBgn0029629 CG8636 CG8636 LD24347<br>FBgn0039537 CG5590 CG5590 GH01709<br>FBgn0039338 XNP CG4548 SD07188<br>FBgn0031493 CG3605 CG3605 LD45152<br>FBgn0052956 CG32956 CG32956 RE06926<br>FBgn0036973 CG5585 CG5585 RE17089<br>FBgn0013347 TfIIA-S CG5163 RE44302<br>FBgn0032847 Taf18 CG10756 RE56212<br>FBgn0011638 La CG10922 RE69138<br>FBgn0011715 Snr1 CG1064 GH08712<br>FBgn0032130 CG3838 CG3838 LD21447<br>FBgn0051365 CG31365 CG31365 LD28458<br>FBgn0015903 apt CG5393 LD45581<br>FBgn0000611 exd CG8933 LD03509<br>FBgn0029903 CG4532 CG4532 LD08951<br>FBgn0003346 Sd CG9999 LD16356<br>FBgn0032919 CG9253 CG9253 RE27528
	FBgn0040230 dbo CG6224 RE13447<br>FBgn0000611 exd CG8933 LD03509<br>FBgn0003346 Sd CG9999 LD16356<br>FBgn0045064 bwa CG13969 RE26924
