# Supplementary material for: Prediction of Gene Expression in Embryonic Structures of Drosophila melanogaster
Source: PLoS Comput Biol. 2007 Jul 20;3(7):e144. doi: 10.1371/journal.pcbi.0030144 (PMC1924873; doi:10.1371/journal.pcbi.0030144)
Supplement: Supplementary file 15 [file pcbi.0030144.st015.pdf]

Table 15: **Diversity of anatomical annotations of genes comprising functional unit tma2smusclep**: somatic muscle primordium && trunk mesoderm primordium && trunk mesoderm anlage

Anatomical Structures	Genes
ventral ectoderm anlage;	
	FBgn0031035 NULL NULL LP04958
	FBgn0039537 CG5590 CG5590 GH01709
	FBgn0031745 CG8965 CG8965 GH01721
	FBgn0033992 CG11798 CG11798 LD23883
	FBgn0000577 en CG9015 LD16125
	FBgn0000492 Dr CG1897 LD04235
	FBgn0031516 CG9663 CG9663 GH24286
	FBgn0000577 en CG9015 LD16125
embryonic optic lobe primordium;	
	FBgn0033992 CG11798 CG11798 LD23883
midline primordium;	
	FBgn0000577 en CG9015 LD16125
	FBgn0000577 en CG9015 LD16125
hindgut proper primordium;	
	FBgn0037707 RnpS1 CG16788 LD23870
	FBgn0031035 NULL NULL LP04958
	FBgn0031745 CG8965 CG8965 GH01721
	FBgn0038178 CG9322 CG9322 GH13441
	FBgn0038146 CG9799 CG9799 SD06427
	FBgn0000577 en CG9015 LD16125
	FBgn0052920 CG32920 CG32920 RE19605
	FBgn0010926 l(3)07882 CG5824 LD15947
	FBgn0000577 en CG9015 LD16125
fat body/gonad primordium;	
	FBgn0040297 NHE2 CG9256 RE21674
dorsal pharyngeal muscle primordium;	
	FBgn0010292 bys CG1430 LD14510
	FBgn0011656 Mef2 CG1429 LD08608
	FBgn0035600 CG4769 CG4769 GM14501
	FBgn0001961 Sop2 CG8978 SD06579
	FBgn0015513 mbc CG10379 SD06708
	FBgn0003900 twi CG2956 AT15089
	FBgn0033992 CG11798 CG11798 LD23883
	FBgn0039039 lmd CG4677 LD47926
	FBgn0011656 Mef2 CG1429 LD08608
	FBgn0032168 CG13126 CG13126 RE01590
	FBgn0052372 CG32372 CG32372 RE09158
	FBgn0052920 CG32920 CG32920 RE19605
	FBgn0033562 CG6751 CG6751 RE21021
	FBgn0040297 NHE2 CG9256 RE21674
	FBgn0031851 CG11188 CG11188 RE30678
	FBgn0031516 CG9663 CG9663 GH24286

continued on next page...

Table 15 continued from previous page.	
Anatomical Structures	Genes
proventriculus primordium;	
	FBgn0037707 RnpS1 CG16788 LD23870
	FBgn0015919 caup CG10605 LP03275
head mesoderm anlage;	
	FBgn0011656 Mef2 CG1429 LD08608
	FBgn0010926 l(3)07882 CG5824 LD15947
	FBgn0037707 RnpS1 CG16788 LD23870
	FBgn0031035 NULL NULL LP04958
	FBgn0013951 Mer CG14228 GH01330
	FBgn0039537 CG5590 CG5590 GH01709
	FBgn0015513 mbc CG10379 SD06708
	FBgn0003900 twi CG2956 AT15089
	FBgn0033992 CG11798 CG11798 LD23883
	FBgn0000472 dm CG10798 LD32539
	FBgn0038146 CG9799 CG9799 SD06427
	FBgn0052372 CG32372 CG32372 RE09158
	FBgn0052920 CG32920 CG32920 RE19605
	FBgn0040297 NHE2 CG9256 RE21674
	FBgn0031851 CG11188 CG11188 RE30678
	FBgn0010926 l(3)07882 CG5824 LD15947
	FBgn0027619 Adam CG12131 GH12681
	FBgn0031516 CG9663 CG9663 GH24286
procephalic ectoderm primordium;	
	FBgn0037707 RnpS1 CG16788 LD23870
	FBgn0039537 CG5590 CG5590 GH01709
	FBgn0033992 CG11798 CG11798 LD23883
	FBgn0000577 en CG9015 LD16125
	FBgn0040297 NHE2 CG9256 RE21674
	FBgn0000577 en CG9015 LD16125
tracheal primordium;	
	FBgn0031745 CG8965 CG8965 GH01721
	FBgn0000472 dm CG10798 LD32539
	FBgn0039039 lmd CG4677 LD47926
dorsal ectoderm primordium;	
	FBgn0038178 CG9322 CG9322 GH13441
	FBgn0033992 CG11798 CG11798 LD23883
	FBgn0039039 lmd CG4677 LD47926
external foregut primordium;	
	FBgn0031745 CG8965 CG8965 GH01721
	FBgn0000577 en CG9015 LD16125
	FBgn0000492 Dr CG1897 LD04235
	FBgn0033562 CG6751 CG6751 RE21021
	FBgn0000577 en CG9015 LD16125
longitudinal visceral mesoderm primordium;	
	FBgn0036298 CG10627 CG10627 LD02044
	FBgn0011656 Mef2 CG1429 LD08608
	continued on next page...

Table 15 continued from previous page.

Anatomical Structures	Genes
	FBgn0015513 mbc CG10379 SD06708<br>FBgn0003900 twi CG2956 AT15089<br>FBgn0033992 CG11798 CG11798 LD23883<br>FBgn0038146 CG9799 CG9799 SD06427<br>FBgn0011656 Mef2 CG1429 LD08608<br>FBgn0033562 CG6751 CG6751 RE21021<br>FBgn0036298 CG10627 CG10627 LD02044<br>FBgn0031516 CG9663 CG9663 GH24286
procephalic neuroblasts;	FBgn0033992 CG11798 CG11798 LD23883
dorsal epidermis primordium;	FBgn0038178 CG9322 CG9322 GH13441<br>FBgn0033992 CG11798 CG11798 LD23883<br>FBgn0039039 lmd CG4677 LD47926<br>FBgn0000577 en CG9015 LD16125<br>FBgn0000577 en CG9015 LD16125
Malpighian tubule primordium;	FBgn0035600 CG4769 CG4769 GM14501<br>FBgn0027619 Adam CG12131 GH12681
garland cell primordium;	FBgn0040297 NHE2 CG9256 RE21674
salivary gland body primordium;	FBgn0000472 dm CG10798 LD32539<br>FBgn0038146 CG9799 CG9799 SD06427<br>FBgn0000492 Dr CG1897 LD04235<br>FBgn0033562 CG6751 CG6751 RE21021<br>FBgn0031851 CG11188 CG11188 RE30678<br>FBgn0036298 CG10627 CG10627 LD02044<br>FBgn0027619 Adam CG12131 GH12681
ventral neuroderm anlage;	FBgn0037707 RnpS1 CG16788 LD23870<br>FBgn0031745 CG8965 CG8965 GH01721<br>FBgn0033992 CG11798 CG11798 LD23883<br>FBgn0000577 en CG9015 LD16125<br>FBgn0000492 Dr CG1897 LD04235<br>FBgn0000577 en CG9015 LD16125
trunk mesoderm anlage;	FBgn0010292 bys CG1430 LD14510<br>FBgn0036298 CG10627 CG10627 LD02044<br>FBgn0011656 Mef2 CG1429 LD08608<br>FBgn0010926 l(3)07882 CG5824 LD15947<br>FBgn0035600 CG4769 CG4769 GM14501<br>FBgn0037707 RnpS1 CG16788 LD23870<br>FBgn0031035 NULL NULL LP04958<br>FBgn0029715 CG11444 CG11444 GM14292<br>FBgn0038585 CG7993 CG7993 GH01229

continued on next page...

Table 15 continued from previous page.

Anatomical Structures	Genes
	FBgn0013951 Mer CG14228 GH01330<br>FBgn0039537 CG5590 CG5590 GH01709<br>FBgn0031745 CG8965 CG8965 GH01721<br>FBgn0001961 Sop2 CG8978 SD06579<br>FBgn0038178 CG9322 CG9322 GH13441<br>FBgn0015513 mbc CG10379 SD06708<br>FBgn0003900 twi CG2956 AT15089<br>FBgn0015919 caup CG10605 LP03275<br>FBgn0033992 CG11798 CG11798 LD23883<br>FBgn0000472 dm CG10798 LD32539<br>FBgn0039039 lmd CG4677 LD47926<br>FBgn0038146 CG9799 CG9799 SD06427<br>FBgn0000577 en CG9015 LD16125<br>FBgn0000492 Dr CG1897 LD04235<br>FBgn0011656 Mef2 CG1429 LD08608<br>FBgn0032168 CG13126 CG13126 RE01590<br>FBgn0052372 CG32372 CG32372 RE09158<br>FBgn0052920 CG32920 CG32920 RE19605<br>FBgn0033562 CG6751 CG6751 RE21021<br>FBgn0040297 NHE2 CG9256 RE21674<br>FBgn0031851 CG11188 CG11188 RE30678<br>FBgn0036298 CG10627 CG10627 LD02044<br>FBgn0010926 l(3)07882 CG5824 LD15947<br>FBgn0027619 Adam CG12131 GH12681<br>FBgn0031516 CG9663 CG9663 GH24286<br>FBgn0000577 en CG9015 LD16125
anterior endoderm anlage;	
	FBgn0010926 l(3)07882 CG5824 LD15947<br>FBgn0037707 RnpS1 CG16788 LD23870<br>FBgn0031035 NULL NULL LP04958<br>FBgn0029715 CG11444 CG11444 GM14292<br>FBgn0038585 CG7993 CG7993 GH01229<br>FBgn0039537 CG5590 CG5590 GH01709<br>FBgn0015513 mbc CG10379 SD06708<br>FBgn0003900 twi CG2956 AT15089<br>FBgn0000472 dm CG10798 LD32539<br>FBgn0038146 CG9799 CG9799 SD06427<br>FBgn0052372 CG32372 CG32372 RE09158<br>FBgn0052920 CG32920 CG32920 RE19605<br>FBgn0031851 CG11188 CG11188 RE30678<br>FBgn0010926 l(3)07882 CG5824 LD15947<br>FBgn0027619 Adam CG12131 GH12681
need new term(larval eye primordium);	
	FBgn0033992 CG11798 CG11798 LD23883
ventral nerve cord primordium;	
	FBgn0037707 RnpS1 CG16788 LD23870

continued on next page...

Table 15 continued from previous page.

Anatomical Structures	Genes
	FBgn0039537 CG5590 CG5590 GH01709<br>FBgn0031745 CG8965 CG8965 GH01721<br>FBgn0033992 CG11798 CG11798 LD23883<br>FBgn0000577 en CG9015 LD16125<br>FBgn0000492 Dr CG1897 LD04235<br>FBgn0040297 NHE2 CG9256 RE21674<br>FBgn0000577 en CG9015 LD16125
visual primordium;	FBgn0033992 CG11798 CG11798 LD23883
procephalic ectoderm anlage;	FBgn0036298 CG10627 CG10627 LD02044<br>FBgn0037707 RnpS1 CG16788 LD23870<br>FBgn0031035 NULL NULL LP04958<br>FBgn0033992 CG11798 CG11798 LD23883<br>FBgn0038146 CG9799 CG9799 SD06427<br>FBgn0000577 en CG9015 LD16125<br>FBgn0036298 CG10627 CG10627 LD02044<br>FBgn0000577 en CG9015 LD16125
dorsal ectoderm anlage;	FBgn0033992 CG11798 CG11798 LD23883<br>FBgn0000577 en CG9015 LD16125<br>FBgn0000577 en CG9015 LD16125
mesectoderm primordium;	FBgn0031745 CG8965 CG8965 GH01721<br>FBgn0033992 CG11798 CG11798 LD23883<br>FBgn0000577 en CG9015 LD16125<br>FBgn0000577 en CG9015 LD16125
foregut primordium;	FBgn0031745 CG8965 CG8965 GH01721<br>FBgn0033562 CG6751 CG6751 RE21021
posterior endoderm anlage;	FBgn0010926 l(3)07882 CG5824 LD15947<br>FBgn0037707 RnpS1 CG16788 LD23870<br>FBgn0031035 NULL NULL LP04958<br>FBgn0029715 CG11444 CG11444 GM14292<br>FBgn0038585 CG7993 CG7993 GH01229<br>FBgn0039537 CG5590 CG5590 GH01709<br>FBgn0015513 mbc CG10379 SD06708<br>FBgn0000472 dm CG10798 LD32539<br>FBgn0038146 CG9799 CG9799 SD06427<br>FBgn0052920 CG32920 CG32920 RE19605<br>FBgn0031851 CG11188 CG11188 RE30678<br>FBgn0010926 l(3)07882 CG5824 LD15947<br>FBgn0027619 Adam CG12131 GH12681
anal pad specific anlage;	FBgn0035600 CG4769 CG4769 GM14501

continued on next page...

Anatomical Structures	Genes
Table 15 continued from previous page.	
	FBgn0010926 l(3)07882 CG5824 LD15947
mesectoderm anlage;	
	FBgn0037707 RnpS1 CG16788 LD23870
	FBgn0031745 CG8965 CG8965 GH01721
	FBgn0033992 CG11798 CG11798 LD23883
	FBgn0000577 en CG9015 LD16125
	FBgn0000577 en CG9015 LD16125
visceral muscle primordium;	
	FBgn0011656 Mef2 CG1429 LD08608
	FBgn0010926 l(3)07882 CG5824 LD15947
	FBgn0037707 RnpS1 CG16788 LD23870
	FBgn0031745 CG8965 CG8965 GH01721
	FBgn0038178 CG9322 CG9322 GH13441
	FBgn0033992 CG11798 CG11798 LD23883
	FBgn0011656 Mef2 CG1429 LD08608
	FBgn0033562 CG6751 CG6751 RE21021
	FBgn0010926 l(3)07882 CG5824 LD15947
	FBgn0031516 CG9663 CG9663 GH24286
salivary gland body specific anlage;	
	FBgn0000472 dm CG10798 LD32539
trunk mesoderm primordium;	
	FBgn0010292 bys CG1430 LD14510
	FBgn0036298 CG10627 CG10627 LD02044
	FBgn0011656 Mef2 CG1429 LD08608
	FBgn0010926 l(3)07882 CG5824 LD15947
	FBgn0035600 CG4769 CG4769 GM14501
	FBgn0037707 RnpS1 CG16788 LD23870
	FBgn0031035 NULL NULL LP04958
	FBgn0029715 CG11444 CG11444 GM14292
	FBgn0038585 CG7993 CG7993 GH01229
	FBgn0013951 Mer CG14228 GH01330
	FBgn0039537 CG5590 CG5590 GH01709
	FBgn0031745 CG8965 CG8965 GH01721
	FBgn0001961 Sop2 CG8978 SD06579
	FBgn0038178 CG9322 CG9322 GH13441
	FBgn0015513 mbc CG10379 SD06708
	FBgn0003900 twi CG2956 AT15089
	FBgn0015919 caup CG10605 LP03275
	FBgn0033992 CG11798 CG11798 LD23883
	FBgn0000472 dm CG10798 LD32539
	FBgn0039039 lmd CG4677 LD47926
	FBgn0038146 CG9799 CG9799 SD06427
	FBgn0000577 en CG9015 LD16125
	FBgn0000492 Dr CG1897 LD04235
	FBgn0011656 Mef2 CG1429 LD08608
	FBgn0032168 CG13126 CG13126 RE01590
	continued on next page...

Table 15 continued from previous page.

Anatomical Structures	Genes
	FBgn0052372 CG32372 CG32372 RE09158<br>FBgn0052920 CG32920 CG32920 RE19605<br>FBgn0033562 CG6751 CG6751 RE21021<br>FBgn0040297 NHE2 CG9256 RE21674<br>FBgn0031851 CG11188 CG11188 RE30678<br>FBgn0036298 CG10627 CG10627 LD02044<br>FBgn0010926 l(3)07882 CG5824 LD15947<br>FBgn0027619 Adam CG12131 GH12681<br>FBgn0031516 CG9663 CG9663 GH24286<br>FBgn0000577 en CG9015 LD16125
protocerebrum primordium;	FBgn0037707 RnpS1 CG16788 LD23870<br>FBgn0033992 CG11798 CG11798 LD23883<br>FBgn0000577 en CG9015 LD16125<br>FBgn0040297 NHE2 CG9256 RE21674<br>FBgn0000577 en CG9015 LD16125
ventral epidermis primordium;	FBgn0038178 CG9322 CG9322 GH13441<br>FBgn0033992 CG11798 CG11798 LD23883<br>FBgn0000577 en CG9015 LD16125<br>FBgn0000492 Dr CG1897 LD04235<br>FBgn0000577 en CG9015 LD16125
ventral ectoderm primordium;	FBgn0038178 CG9322 CG9322 GH13441<br>FBgn0033992 CG11798 CG11798 LD23883<br>FBgn0039039 lmd CG4677 LD47926<br>FBgn0000577 en CG9015 LD16125<br>FBgn0000492 Dr CG1897 LD04235<br>FBgn0000577 en CG9015 LD16125
visual anlage;	FBgn0033992 CG11798 CG11798 LD23883
neuroblasts of ventral nervous system;	FBgn0033992 CG11798 CG11798 LD23883<br>FBgn0040297 NHE2 CG9256 RE21674
inclusive hindgut primordium;	FBgn0036298 CG10627 CG10627 LD02044<br>FBgn0035600 CG4769 CG4769 GM14501<br>FBgn0037707 RnpS1 CG16788 LD23870<br>FBgn0031035 NULL NULL LP04958<br>FBgn0038585 CG7993 CG7993 GH01229<br>FBgn0031745 CG8965 CG8965 GH01721<br>FBgn0038178 CG9322 CG9322 GH13441<br>FBgn0038146 CG9799 CG9799 SD06427<br>FBgn0000577 en CG9015 LD16125<br>FBgn0052920 CG32920 CG32920 RE19605<br>FBgn0033562 CG6751 CG6751 RE21021

continued on next page...

Table 15 continued from previous page.	
Anatomical Structures	Genes
	FBgn0036298 CG10627 CG10627 LD02044
	FBgn0027619 Adam CG12131 GH12681
	FBgn0000577 en CG9015 LD16125
somatic muscle primordium;	
	FBgn0010292 bys CG1430 LD14510
	FBgn0036298 CG10627 CG10627 LD02044
	FBgn0011656 Mef2 CG1429 LD08608
	FBgn0010926 l(3)07882 CG5824 LD15947
	FBgn0035600 CG4769 CG4769 GM14501
	FBgn0037707 RnpS1 CG16788 LD23870
	FBgn0031035 NULL NULL LP04958
	FBgn0029715 CG11444 CG11444 GM14292
	FBgn0038585 CG7993 CG7993 GH01229
	FBgn0013951 Mer CG14228 GH01330
	FBgn0039537 CG5590 CG5590 GH01709
	FBgn0031745 CG8965 CG8965 GH01721
	FBgn0001961 Sop2 CG8978 SD06579
	FBgn0038178 CG9322 CG9322 GH13441
	FBgn0015513 mbc CG10379 SD06708
	FBgn0003900 twi CG2956 AT15089
	FBgn0015919 caup CG10605 LP03275
	FBgn0033992 CG11798 CG11798 LD23883
	FBgn0000472 dm CG10798 LD32539
	FBgn0039039 lmd CG4677 LD47926
	FBgn0038146 CG9799 CG9799 SD06427
	FBgn0000577 en CG9015 LD16125
	FBgn0000492 Dr CG1897 LD04235
	FBgn0011656 Mef2 CG1429 LD08608
	FBgn0032168 CG13126 CG13126 RE01590
	FBgn0052372 CG32372 CG32372 RE09158
	FBgn0052920 CG32920 CG32920 RE19605
	FBgn0033562 CG6751 CG6751 RE21021
	FBgn0040297 NHE2 CG9256 RE21674
	FBgn0031851 CG11188 CG11188 RE30678
	FBgn0036298 CG10627 CG10627 LD02044
	FBgn0010926 l(3)07882 CG5824 LD15947
	FBgn0027619 Adam CG12131 GH12681
	FBgn0031516 CG9663 CG9663 GH24286
	FBgn0000577 en CG9015 LD16125
foregut anlage;	
	FBgn0037707 RnpS1 CG16788 LD23870
	FBgn0033562 CG6751 CG6751 RE21021
gnathal primordium;	
	FBgn0000577 en CG9015 LD16125
	FBgn0000492 Dr CG1897 LD04235
	FBgn0000577 en CG9015 LD16125
	continued on next page...

Table 15 continued from previous page.	
Anatomical Structures	Genes
hindgut anlage;	
	FBgn0036298 CG10627 CG10627 LD02044
	FBgn0035600 CG4769 CG4769 GM14501
	FBgn0037707 RnpS1 CG16788 LD23870
	FBgn0031035 NULL NULL LP04958
	FBgn0038585 CG7993 CG7993 GH01229
	FBgn0031745 CG8965 CG8965 GH01721
	FBgn0038178 CG9322 CG9322 GH13441
	FBgn0038146 CG9799 CG9799 SD06427
	FBgn0000577 en CG9015 LD16125
	FBgn0033562 CG6751 CG6751 RE21021
	FBgn0036298 CG10627 CG10627 LD02044
	FBgn0000577 en CG9015 LD16125
stomatogastric nervous system primordium;	
	FBgn0031745 CG8965 CG8965 GH01721
	FBgn0000492 Dr CG1897 LD04235
clypeo-labral primordium;	
	FBgn0000577 en CG9015 LD16125
	FBgn0000577 en CG9015 LD16125
anterior midgut primordium;	
	FBgn0010292 bys CG1430 LD14510
	FBgn0010926 l(3)07882 CG5824 LD15947
	FBgn0037707 RnpS1 CG16788 LD23870
	FBgn0029715 CG11444 CG11444 GM14292
	FBgn0038585 CG7993 CG7993 GH01229
	FBgn0039537 CG5590 CG5590 GH01709
	FBgn0015513 mbc CG10379 SD06708
	FBgn0000472 dm CG10798 LD32539
	FBgn0038146 CG9799 CG9799 SD06427
	FBgn0032168 CG13126 CG13126 RE01590
	FBgn0052920 CG32920 CG32920 RE19605
	FBgn0033562 CG6751 CG6751 RE21021
	FBgn0040297 NHE2 CG9256 RE21674
	FBgn0031851 CG11188 CG11188 RE30678
	FBgn0036298 CG10627 CG10627 LD02044
	FBgn0010926 l(3)07882 CG5824 LD15947
	FBgn0027619 Adam CG12131 GH12681
head mesoderm P2 primordium;	
	FBgn0010292 bys CG1430 LD14510
	FBgn0036298 CG10627 CG10627 LD02044
	FBgn0010926 l(3)07882 CG5824 LD15947
	FBgn0035600 CG4769 CG4769 GM14501
	FBgn0013951 Mer CG14228 GH01330
	FBgn0039537 CG5590 CG5590 GH01709
	FBgn0001961 Sop2 CG8978 SD06579
	FBgn0038178 CG9322 CG9322 GH13441
	continued on next page...

Table 15 continued from previous page.

Anatomical Structures	Genes
	FBgn0015513 mbc CG10379 SD06708<br>FBgn0003900 twi CG2956 AT15089<br>FBgn0033992 CG11798 CG11798 LD23883<br>FBgn0000472 dm CG10798 LD32539<br>FBgn0039039 lmd CG4677 LD47926<br>FBgn0052372 CG32372 CG32372 RE09158<br>FBgn0052920 CG32920 CG32920 RE19605<br>FBgn0033562 CG6751 CG6751 RE21021<br>FBgn0040297 NHE2 CG9256 RE21674<br>FBgn0031851 CG11188 CG11188 RE30678<br>FBgn0036298 CG10627 CG10627 LD02044<br>FBgn0010926 l(3)07882 CG5824 LD15947<br>FBgn0027619 Adam CG12131 GH12681<br>FBgn0031516 CG9663 CG9663 GH24286
anterior endoderm primordium;	
	FBgn0010292 bys CG1430 LD14510<br>FBgn0010926 l(3)07882 CG5824 LD15947<br>FBgn0037707 RnpS1 CG16788 LD23870<br>FBgn0031035 NULL NULL LP04958<br>FBgn0029715 CG11444 CG11444 GM14292<br>FBgn0038585 CG7993 CG7993 GH01229<br>FBgn0039537 CG5590 CG5590 GH01709<br>FBgn0015513 mbc CG10379 SD06708<br>FBgn0000472 dm CG10798 LD32539<br>FBgn0038146 CG9799 CG9799 SD06427<br>FBgn0032168 CG13126 CG13126 RE01590<br>FBgn0052920 CG32920 CG32920 RE19605<br>FBgn0033562 CG6751 CG6751 RE21021<br>FBgn0040297 NHE2 CG9256 RE21674<br>FBgn0031851 CG11188 CG11188 RE30678<br>FBgn0036298 CG10627 CG10627 LD02044<br>FBgn0010926 l(3)07882 CG5824 LD15947<br>FBgn0027619 Adam CG12131 GH12681
cardiac mesoderm primordium;	
	FBgn0033992 CG11798 CG11798 LD23883<br>FBgn0040297 NHE2 CG9256 RE21674
posterior midgut primordium;	
	FBgn0010292 bys CG1430 LD14510<br>FBgn0010926 l(3)07882 CG5824 LD15947<br>FBgn0037707 RnpS1 CG16788 LD23870<br>FBgn0029715 CG11444 CG11444 GM14292<br>FBgn0038585 CG7993 CG7993 GH01229<br>FBgn0039537 CG5590 CG5590 GH01709<br>FBgn0015513 mbc CG10379 SD06708<br>FBgn0000472 dm CG10798 LD32539<br>FBgn0038146 CG9799 CG9799 SD06427

continued on next page...

Table 15 continued from previous page.	
Anatomical Structures	Genes
	FBgn0032168 CG13126 CG13126 RE01590<br>FBgn0052920 CG32920 CG32920 RE19605<br>FBgn0033562 CG6751 CG6751 RE21021<br>FBgn0040297 NHE2 CG9256 RE21674<br>FBgn0031851 CG11188 CG11188 RE30678<br>FBgn0036298 CG10627 CG10627 LD02044<br>FBgn0010926 l(3)07882 CG5824 LD15947<br>FBgn0027619 Adam CG12131 GH12681
embryonic ganglion mother cell;	FBgn0040297 NHE2 CG9256 RE21674
lamellocyte specific anlage;	FBgn0033562 CG6751 CG6751 RE21021<br>FBgn0040297 NHE2 CG9256 RE21674
posterior endoderm primordium;	FBgn0010292 bys CG1430 LD14510<br>FBgn0010926 l(3)07882 CG5824 LD15947<br>FBgn0037707 RnpS1 CG16788 LD23870<br>FBgn0031035 NULL NULL LP04958<br>FBgn0029715 CG11444 CG11444 GM14292<br>FBgn0038585 CG7993 CG7993 GH01229<br>FBgn0039537 CG5590 CG5590 GH01709<br>FBgn0038178 CG9322 CG9322 GH13441<br>FBgn0015513 mbc CG10379 SD06708<br>FBgn0000472 dm CG10798 LD32539<br>FBgn0032168 CG13126 CG13126 RE01590<br>FBgn0052920 CG32920 CG32920 RE19605<br>FBgn0033562 CG6751 CG6751 RE21021<br>FBgn0040297 NHE2 CG9256 RE21674<br>FBgn0031851 CG11188 CG11188 RE30678<br>FBgn0036298 CG10627 CG10627 LD02044<br>FBgn0010926 l(3)07882 CG5824 LD15947<br>FBgn0027619 Adam CG12131 GH12681
fat body specific anlage;	FBgn0027619 Adam CG12131 GH12681
