# Supplementary material for: Prediction of Gene Expression in Embryonic Structures of Drosophila melanogaster
Source: PLoS Comput Biol. 2007 Jul 20;3(7):e144. doi: 10.1371/journal.pcbi.0030144 (PMC1924873; doi:10.1371/journal.pcbi.0030144)
Supplement: Supplementary file 16 [file pcbi.0030144.st016.pdf]

Table 16: **Diversity of anatomical annotations of genes comprising functional unit vna2lcord**: ventral neuroderm anlage && ventral nerve cord primordium && lateral cord

Anatomical Structures	Genes
embryonic optic lobe primordium;	
	FBgn0002932 neur CG11988 LD45505
	FBgn0033992 CG11798 CG11798 LD23883
	FBgn0010453 Wnt4 CG4698 RE26454
embryonic esophagus;	
	FBgn0031745 CG8965 CG8965 GH01721
	FBgn0010453 Wnt4 CG4698 RE26454
dorsal pharyngeal muscle primordium;	
	FBgn0033992 CG11798 CG11798 LD23883
embryonic epipharynx;	
	FBgn0031745 CG8965 CG8965 GH01721
	FBgn0015903 apt CG5393 LD45581
	FBgn0000577 en CG9015 LD16125
	FBgn0010453 Wnt4 CG4698 RE26454
	FBgn0000577 en CG9015 LD16125
ventral midline;	
	FBgn0031745 CG8965 CG8965 GH01721
	FBgn0040271 Sulf1 CG6725 SD04414
	FBgn0000577 en CG9015 LD16125
	FBgn0000411 D CG5893 AT02631
	FBgn0015773 NetA CG18657 RE11206
	FBgn0010105 comm CG17943 RH64772
	FBgn0000577 en CG9015 LD16125
gonad;	
	FBgn0032130 CG3838 CG3838 LD21447
	FBgn0038434 CG14897 CG14897 RE60459
	FBgn0029512 Aos1 CG12276 LD33652
	FBgn0017453 Zn72D CG5215 GH12756
head mesoderm anlage;	
	FBgn0037707 RnpS1 CG16788 LD23870
	FBgn0002932 neur CG11988 LD45505
	FBgn0032130 CG3838 CG3838 LD21447
	FBgn0033992 CG11798 CG11798 LD23883
	FBgn0000611 exd CG8933 LD03509
	FBgn0003346 Sd CG9999 LD16356
	FBgn0015773 NetA CG18657 RE11206
	FBgn0052982 CG32982 CG32982 RE11317
embryonic midgut;	
	FBgn0037707 RnpS1 CG16788 LD23870
	FBgn0000071 Ama CG2198 LD39923
	FBgn0040271 Sulf1 CG6725 SD04414
	FBgn0032130 CG3838 CG3838 LD21447
	FBgn0000611 exd CG8933 LD03509

continued on next page...

Table 16 continued from previous page.	
Anatomical Structures	Genes
	FBgn0038118 NULL CG7855 RE03551
	FBgn0010453 Wnt4 CG4698 RE26454
embryonic proventriculus;	
	FBgn0032130 CG3838 CG3838 LD21447
	FBgn0015773 NetA CG18657 RE11206
	FBgn0010453 Wnt4 CG4698 RE26454
procephalic ectoderm primordium;	
	FBgn0036494 Toll-6 CG7250 LD08841
	FBgn0033802 CG17724 CG17724 LD12453
	FBgn0037707 RnpS1 CG16788 LD23870
	FBgn0000071 Ama CG2198 LD39923
	FBgn0002932 neur CG11988 LD45505
	FBgn0040271 Sulf1 CG6725 SD04414
	FBgn0040230 dbo CG6224 RE13447
	FBgn0002561 l(1)sc CG3839 RE59335
	FBgn0003300 run CG1849 GH02614
	FBgn0032130 CG3838 CG3838 LD21447
	FBgn0015903 apt CG5393 LD45581
	FBgn0033992 CG11798 CG11798 LD23883
	FBgn0000611 exd CG8933 LD03509
	FBgn0000577 en CG9015 LD16125
	FBgn0003346 Sd CG9999 LD16356
	FBgn0038118 NULL CG7855 RE03551
	FBgn0015773 NetA CG18657 RE11206
	FBgn0015271 Orc5 CG7833 RE16687
	FBgn0031878 CG9188 CG9188 RE16905
	FBgn0010453 Wnt4 CG4698 RE26454
	FBgn0030507 CG11164 CG11164 RE50056
	FBgn0012066 DNApol-delta CG5949 RE60511
	FBgn0032906 CG9273 CG9273 RH07841
	FBgn0010105 comm CG17943 RH64772
	FBgn0029512 Aos1 CG12276 LD33652
	FBgn0017453 Zn72D CG5215 GH12756
	FBgn0015270 Orc2 CG3041 GH13824
	FBgn0000577 en CG9015 LD16125
embryonic/larval circulatory system;	
	FBgn0015903 apt CG5393 LD45581
dorsal ectoderm primordium;	
	FBgn0036494 Toll-6 CG7250 LD08841
	FBgn0000071 Ama CG2198 LD39923
	FBgn0040271 Sulf1 CG6725 SD04414
	FBgn0010109 dpn CG8704 RE34993
	FBgn0003300 run CG1849 GH02614
	FBgn0015903 apt CG5393 LD45581
	FBgn0033992 CG11798 CG11798 LD23883
	FBgn0000611 exd CG8933 LD03509
	continued on next page...

Table 16 continued from previous page.	
Anatomical Structures	Genes
	FBgn0003346 Sd CG9999 LD16356<br>FBgn0010105 comm CG17943 RH64772
dorsal epidermis primordium;	FBgn0036494 Toll-6 CG7250 LD08841<br>FBgn0040271 Sulf1 CG6725 SD04414<br>FBgn0033992 CG11798 CG11798 LD23883<br>FBgn0000611 exd CG8933 LD03509<br>FBgn0000577 en CG9015 LD16125<br>FBgn0010453 Wnt4 CG4698 RE26454<br>FBgn0010105 comm CG17943 RH64772<br>FBgn0000577 en CG9015 LD16125
embryonic stomatogastric nervous system;	FBgn0031745 CG8965 CG8965 GH01721<br>FBgn0002561 l(1)sc CG3839 RE59335<br>FBgn0000492 Dr CG1897 LD04235
ventral neuroderm anlage;	FBgn0036494 Toll-6 CG7250 LD08841<br>FBgn0033802 CG17724 CG17724 LD12453<br>FBgn0037707 RnpS1 CG16788 LD23870<br>FBgn0031745 CG8965 CG8965 GH01721<br>FBgn0000071 Ama CG2198 LD39923<br>FBgn0002932 neur CG11988 LD45505<br>FBgn0040271 Sulf1 CG6725 SD04414<br>FBgn0040230 dbo CG6224 RE13447<br>FBgn0002561 l(1)sc CG3839 RE59335<br>FBgn0010109 dpn CG8704 RE34993<br>FBgn0003300 run CG1849 GH02614<br>FBgn0032130 CG3838 CG3838 LD21447<br>FBgn0015903 apt CG5393 LD45581<br>FBgn0033992 CG11798 CG11798 LD23883<br>FBgn0000611 exd CG8933 LD03509<br>FBgn0000577 en CG9015 LD16125<br>FBgn0003346 Sd CG9999 LD16356<br>FBgn0000492 Dr CG1897 LD04235<br>FBgn0000411 D CG5893 AT02631<br>FBgn0038118 NULL CG7855 RE03551<br>FBgn0015773 NetA CG18657 RE11206<br>FBgn0052982 CG32982 CG32982 RE11317<br>FBgn0015271 Orc5 CG7833 RE16687<br>FBgn0031878 CG9188 CG9188 RE16905<br>FBgn0010453 Wnt4 CG4698 RE26454<br>FBgn0030507 CG11164 CG11164 RE50056<br>FBgn0038434 CG14897 CG14897 RE60459<br>FBgn0012066 DNApol-delta CG5949 RE60511<br>FBgn0032906 CG9273 CG9273 RH07841<br>FBgn0010105 comm CG17943 RH64772
	continued on next page...

Table 16 continued from previous page.	
Anatomical Structures	Genes
	FBgn0029512 Aos1 CG12276 LD33652<br>FBgn0017453 Zn72D CG5215 GH12756<br>FBgn0015270 Orc2 CG3041 GH13824<br>FBgn0000577 en CG9015 LD16125
embryonic/larval somatic muscle;	FBgn0037707 RnpS1 CG16788 LD23870<br>FBgn0031745 CG8965 CG8965 GH01721<br>FBgn0040271 Sulf1 CG6725 SD04414<br>FBgn0000611 exd CG8933 LD03509
need new term(larval eye primordium);	FBgn0033992 CG11798 CG11798 LD23883
embryonic inner optic lobe;	FBgn0010453 Wnt4 CG4698 RE26454
ventral sensory complex specific anlage;	FBgn0002932 neur CG11988 LD45505<br>FBgn0002561 l(1)sc CG3839 RE59335
procephalic ectoderm anlage;	FBgn0036494 Toll-6 CG7250 LD08841<br>FBgn0033802 CG17724 CG17724 LD12453<br>FBgn0037707 RnpS1 CG16788 LD23870<br>FBgn0000071 Ama CG2198 LD39923<br>FBgn0002932 neur CG11988 LD45505<br>FBgn0040230 dbo CG6224 RE13447<br>FBgn0002561 l(1)sc CG3839 RE59335<br>FBgn0003300 run CG1849 GH02614<br>FBgn0015903 apt CG5393 LD45581<br>FBgn0033992 CG11798 CG11798 LD23883<br>FBgn0000611 exd CG8933 LD03509<br>FBgn0000577 en CG9015 LD16125<br>FBgn0003346 Sd CG9999 LD16356<br>FBgn0038118 NULL CG7855 RE03551<br>FBgn0031878 CG9188 CG9188 RE16905<br>FBgn0010453 Wnt4 CG4698 RE26454<br>FBgn0030507 CG11164 CG11164 RE50056<br>FBgn0010105 comm CG17943 RH64772<br>FBgn0029512 Aos1 CG12276 LD33652<br>FBgn0015270 Orc2 CG3041 GH13824<br>FBgn0000577 en CG9015 LD16125
ring gland;	FBgn0015903 apt CG5393 LD45581
dorsal ectoderm anlage;	FBgn0000071 Ama CG2198 LD39923<br>FBgn0010109 dpn CG8704 RE34993<br>FBgn0003300 run CG1849 GH02614<br>FBgn0033992 CG11798 CG11798 LD23883<br>FBgn0000611 exd CG8933 LD03509
	continued on next page...

Table 16 continued from previous page.

Anatomical Structures	Genes
	FBgn0000577 en CG9015 LD16125<br>FBgn0003346 Sd CG9999 LD16356<br>FBgn0000577 en CG9015 LD16125
mesectoderm primordium;	FBgn0031745 CG8965 CG8965 GH01721<br>FBgn0002932 neur CG11988 LD45505<br>FBgn0033992 CG11798 CG11798 LD23883<br>FBgn0000577 en CG9015 LD16125<br>FBgn0003346 Sd CG9999 LD16356<br>FBgn0015773 NetA CG18657 RE11206<br>FBgn0010105 comm CG17943 RH64772<br>FBgn0000577 en CG9015 LD16125
embryonic gastric caecum;	FBgn0010453 Wnt4 CG4698 RE26454
embryonic hypopharynx;	FBgn0031745 CG8965 CG8965 GH01721<br>FBgn0015903 apt CG5393 LD45581
embryonic esophageal ganglion;	FBgn0031745 CG8965 CG8965 GH01721
embryonic/larval dorsal vessel;	FBgn0015903 apt CG5393 LD45581
embryonic central brain pars intercerebralis;	FBgn0040271 Sulf1 CG6725 SD04414<br>FBgn0038118 NULL CG7855 RE03551
embryonic central brain;	FBgn0036494 Toll-6 CG7250 LD08841<br>FBgn0002932 neur CG11988 LD45505<br>FBgn0040271 Sulf1 CG6725 SD04414<br>FBgn0040230 dbo CG6224 RE13447<br>FBgn0002561 l(1)sc CG3839 RE59335<br>FBgn0003300 run CG1849 GH02614<br>FBgn0015903 apt CG5393 LD45581<br>FBgn0033992 CG11798 CG11798 LD23883<br>FBgn0000611 exd CG8933 LD03509<br>FBgn0000577 en CG9015 LD16125<br>FBgn0003346 Sd CG9999 LD16356<br>FBgn0038118 NULL CG7855 RE03551<br>FBgn0015773 NetA CG18657 RE11206<br>FBgn0010453 Wnt4 CG4698 RE26454<br>FBgn0029512 Aos1 CG12276 LD33652<br>FBgn0000577 en CG9015 LD16125
embryonic central brain neuron;	FBgn0002932 neur CG11988 LD45505<br>FBgn0040230 dbo CG6224 RE13447<br>FBgn0033992 CG11798 CG11798 LD23883<br>FBgn0000611 exd CG8933 LD03509

continued on next page...

Table 16 continued from previous page.	
Anatomical Structures	Genes
	FBgn0000577 en CG9015 LD16125<br>FBgn0000411 D CG5893 AT02631<br>FBgn0038118 NULL CG7855 RE03551<br>FBgn0015773 NetA CG18657 RE11206<br>FBgn0010453 Wnt4 CG4698 RE26454<br>FBgn0038434 CG14897 CG14897 RE60459<br>FBgn0029512 Aos1 CG12276 LD33652<br>FBgn0017453 Zn72D CG5215 GH12756<br>FBgn0015270 Orc2 CG3041 GH13824<br>FBgn0000577 en CG9015 LD16125
ventral ectoderm primordium;	FBgn0036494 Toll-6 CG7250 LD08841<br>FBgn0033802 CG17724 CG17724 LD12453<br>FBgn0000071 Ama CG2198 LD39923<br>FBgn0040230 dbo CG6224 RE13447<br>FBgn0010109 dpn CG8704 RE34993<br>FBgn0003300 run CG1849 GH02614<br>FBgn0032130 CG3838 CG3838 LD21447<br>FBgn0015903 apt CG5393 LD45581<br>FBgn0033992 CG11798 CG11798 LD23883<br>FBgn0000611 exd CG8933 LD03509<br>FBgn0000577 en CG9015 LD16125<br>FBgn0003346 Sd CG9999 LD16356<br>FBgn0000492 Dr CG1897 LD04235<br>FBgn0052982 CG32982 CG32982 RE11317<br>FBgn0010453 Wnt4 CG4698 RE26454<br>FBgn0030507 CG11164 CG11164 RE50056<br>FBgn0012066 DNApol-delta CG5949 RE60511<br>FBgn0000577 en CG9015 LD16125
visual anlage;	FBgn0015903 apt CG5393 LD45581<br>FBgn0033992 CG11798 CG11798 LD23883<br>FBgn0000611 exd CG8933 LD03509<br>FBgn0003346 Sd CG9999 LD16356<br>FBgn0010105 comm CG17943 RH64772<br>FBgn0015270 Orc2 CG3041 GH13824
pericardial cell specific anlage;	FBgn0015903 apt CG5393 LD45581
somatic muscle primordium;	FBgn0037707 RnpS1 CG16788 LD23870<br>FBgn0031745 CG8965 CG8965 GH01721<br>FBgn0040271 Sulf1 CG6725 SD04414<br>FBgn0002561 l(1)sc CG3839 RE59335<br>FBgn0033992 CG11798 CG11798 LD23883<br>FBgn0000577 en CG9015 LD16125<br>FBgn0000492 Dr CG1897 LD04235
	continued on next page...

Table 16 continued from previous page.

Anatomical Structures	Genes
	FBgn0000577 en CG9015 LD16125
foregut anlage;	FBgn0037707 RnpS1 CG16788 LD23870<br>FBgn0002932 neur CG11988 LD45505<br>FBgn0015903 apt CG5393 LD45581<br>FBgn0000611 exd CG8933 LD03509<br>FBgn0003346 Sd CG9999 LD16356<br>FBgn0010453 Wnt4 CG4698 RE26454
inclusive hindgut primordium;	FBgn0037707 RnpS1 CG16788 LD23870<br>FBgn0031745 CG8965 CG8965 GH01721<br>FBgn0040230 dbo CG6224 RE13447<br>FBgn0010109 dpn CG8704 RE34993<br>FBgn0003300 run CG1849 GH02614<br>FBgn0015903 apt CG5393 LD45581<br>FBgn0000611 exd CG8933 LD03509<br>FBgn0000577 en CG9015 LD16125<br>FBgn0003346 Sd CG9999 LD16356<br>FBgn0000411 D CG5893 AT02631<br>FBgn0000577 en CG9015 LD16125
circular visceral muscle fibers;	FBgn0015773 NetA CG18657 RE11206
need new term(sensory system head);	FBgn0010109 dpn CG8704 RE34993<br>FBgn0033992 CG11798 CG11798 LD23883<br>FBgn0010105 comm CG17943 RH64772<br>FBgn0017453 Zn72D CG5215 GH12756
lateral cord surface glia;	FBgn0002932 neur CG11988 LD45505<br>FBgn0038118 NULL CG7855 RE03551<br>FBgn0015270 Orc2 CG3041 GH13824
stomatogastric nervous system primordium;	FBgn0031745 CG8965 CG8965 GH01721<br>FBgn0002561 l(1)sc CG3839 RE59335<br>FBgn0000492 Dr CG1897 LD04235
embryonic/larval muscle system;	FBgn0037707 RnpS1 CG16788 LD23870<br>FBgn0000611 exd CG8933 LD03509<br>FBgn0000492 Dr CG1897 LD04235<br>FBgn0015773 NetA CG18657 RE11206
anterior endoderm primordium;	FBgn0037707 RnpS1 CG16788 LD23870<br>FBgn0032130 CG3838 CG3838 LD21447<br>FBgn0015903 apt CG5393 LD45581<br>FBgn0000611 exd CG8933 LD03509<br>FBgn0003346 Sd CG9999 LD16356

continued on next page...

Table 16 continued from previous page.	
Anatomical Structures	Genes
embryonic rectum;	
	FBgn0000492 Dr CG1897 LD04235<br>FBgn0010453 Wnt4 CG4698 RE26454
posterior midgut primordium;	
	FBgn0037707 RnpS1 CG16788 LD23870<br>FBgn0000071 Ama CG2198 LD39923<br>FBgn0002561 l(1)sc CG3839 RE59335<br>FBgn0017453 Zn72D CG5215 GH12756
ventral sensory complex primordium;	
	FBgn0002932 neur CG11988 LD45505<br>FBgn0040271 Sulf1 CG6725 SD04414<br>FBgn0003300 run CG1849 GH02614<br>FBgn0033992 CG11798 CG11798 LD23883
sensory nervous system specific anlage;	
	FBgn0000071 Ama CG2198 LD39923<br>FBgn0002932 neur CG11988 LD45505<br>FBgn0002561 l(1)sc CG3839 RE59335<br>FBgn0010109 dpn CG8704 RE34993<br>FBgn0017453 Zn72D CG5215 GH12756
posterior endoderm primordium;	
	FBgn0037707 RnpS1 CG16788 LD23870<br>FBgn0002561 l(1)sc CG3839 RE59335<br>FBgn0032130 CG3838 CG3838 LD21447<br>FBgn0015903 apt CG5393 LD45581<br>FBgn0000611 exd CG8933 LD03509<br>FBgn0003346 Sd CG9999 LD16356
ventral ectoderm anlage;	
	FBgn0036494 Toll-6 CG7250 LD08841<br>FBgn0031745 CG8965 CG8965 GH01721<br>FBgn0000071 Ama CG2198 LD39923<br>FBgn0002932 neur CG11988 LD45505<br>FBgn0040230 dbo CG6224 RE13447<br>FBgn0002561 l(1)sc CG3839 RE59335<br>FBgn0010109 dpn CG8704 RE34993<br>FBgn0003300 run CG1849 GH02614<br>FBgn0032130 CG3838 CG3838 LD21447<br>FBgn0015903 apt CG5393 LD45581<br>FBgn0033992 CG11798 CG11798 LD23883<br>FBgn0000611 exd CG8933 LD03509<br>FBgn0000577 en CG9015 LD16125<br>FBgn0003346 Sd CG9999 LD16356<br>FBgn0000492 Dr CG1897 LD04235<br>FBgn0000411 D CG5893 AT02631<br>FBgn0038118 NULL CG7855 RE03551<br>FBgn0052982 CG32982 CG32982 RE11317<br>FBgn0010453 Wnt4 CG4698 RE26454

continued on next page...

Table 16 continued from previous page.	
Anatomical Structures	Genes
	FBgn0010105 comm CG17943 RH64772
	FBgn0015270 Orc2 CG3041 GH13824
	FBgn0000577 en CG9015 LD16125
embryonic central brain glia;	
	FBgn0002932 neur CG11988 LD45505
	FBgn0033992 CG11798 CG11798 LD23883
	FBgn0000611 exd CG8933 LD03509
	FBgn0000411 D CG5893 AT02631
	FBgn0029512 Aos1 CG12276 LD33652
	FBgn0017453 Zn72D CG5215 GH12756
	FBgn0015270 Orc2 CG3041 GH13824
embryonic frontal ganglion;	
	FBgn0031745 CG8965 CG8965 GH01721
midline primordium;	
	FBgn0040271 Sulf1 CG6725 SD04414
	FBgn0002561 l(1)sc CG3839 RE59335
	FBgn0000577 en CG9015 LD16125
	FBgn0015773 NetA CG18657 RE11206
	FBgn0010105 comm CG17943 RH64772
	FBgn0000577 en CG9015 LD16125
hindgut proper primordium;	
	FBgn0037707 RnpS1 CG16788 LD23870
	FBgn0031745 CG8965 CG8965 GH01721
	FBgn0040230 dbo CG6224 RE13447
	FBgn0000577 en CG9015 LD16125
	FBgn0000411 D CG5893 AT02631
	FBgn0010453 Wnt4 CG4698 RE26454
	FBgn0017453 Zn72D CG5215 GH12756
	FBgn0000577 en CG9015 LD16125
embryonic/larval visceral muscle;	
	FBgn0037707 RnpS1 CG16788 LD23870
	FBgn0031745 CG8965 CG8965 GH01721
	FBgn0000611 exd CG8933 LD03509
	FBgn0015773 NetA CG18657 RE11206
embryonic dorsal pouch;	
	FBgn0040230 dbo CG6224 RE13447
	FBgn0015903 apt CG5393 LD45581
pars intercerebralis primordium;	
	FBgn0040271 Sulf1 CG6725 SD04414
	FBgn0038118 NULL CG7855 RE03551
ventral midline glia;	
	FBgn0031745 CG8965 CG8965 GH01721
	FBgn0000411 D CG5893 AT02631
embryonic head epidermis;	
	FBgn0033802 CG17724 CG17724 LD12453
	FBgn0040230 dbo CG6224 RE13447
	continued on next page...

Table 16 continued from previous page.	
Anatomical Structures	Genes
	FBgn0033992 CG11798 CG11798 LD23883
	FBgn0000577 en CG9015 LD16125
	FBgn0000492 Dr CG1897 LD04235
	FBgn0015773 NetA CG18657 RE11206
	FBgn0017453 Zn72D CG5215 GH12756
	FBgn0000577 en CG9015 LD16125
lymph gland;	
	FBgn0038118 NULL CG7855 RE03551
	FBgn0032906 CG9273 CG9273 RH07841
proventriculus primordium;	
	FBgn0037707 RnpS1 CG16788 LD23870
	FBgn0003346 Sd CG9999 LD16356
embryonic central brain surface glia;	
	FBgn0002932 neur CG11988 LD45505
	FBgn0015271 Orc5 CG7833 RE16687
	FBgn0030507 CG11164 CG11164 RE50056
	FBgn0012066 DNApol-delta CG5949 RE60511
	FBgn0015270 Orc2 CG3041 GH13824
embryonic main segment of Malpighian tubule;	
	FBgn0012066 DNApol-delta CG5949 RE60511
antennal primordium2;	
	FBgn0003346 Sd CG9999 LD16356
embryonic central nervous system;	
	FBgn0036494 Toll-6 CG7250 LD08841
	FBgn0033802 CG17724 CG17724 LD12453
	FBgn0037707 RnpS1 CG16788 LD23870
	FBgn0002932 neur CG11988 LD45505
	FBgn0040271 Sulf1 CG6725 SD04414
	FBgn0040230 dbo CG6224 RE13447
	FBgn0002561 l(1)sc CG3839 RE59335
	FBgn0010109 dpn CG8704 RE34993
	FBgn0003300 run CG1849 GH02614
	FBgn0015903 apt CG5393 LD45581
	FBgn0033992 CG11798 CG11798 LD23883
	FBgn0000611 exd CG8933 LD03509
	FBgn0000577 en CG9015 LD16125
	FBgn0003346 Sd CG9999 LD16356
	FBgn0038118 NULL CG7855 RE03551
	FBgn0015271 Orc5 CG7833 RE16687
	FBgn0031878 CG9188 CG9188 RE16905
	FBgn0010453 Wnt4 CG4698 RE26454
	FBgn0038434 CG14897 CG14897 RE60459
	FBgn0032906 CG9273 CG9273 RH07841
	FBgn0010105 comm CG17943 RH64772
	FBgn0029512 Aos1 CG12276 LD33652
	FBgn0017453 Zn72D CG5215 GH12756
	continued on next page...

Table 16 continued from previous page.	
Anatomical Structures	Genes
	FBgn0015270 Orc2 CG3041 GH13824
	FBgn0000577 en CG9015 LD16125
embryonic foregut;	
	FBgn0031745 CG8965 CG8965 GH01721
	FBgn0015903 apt CG5393 LD45581
	FBgn0010453 Wnt4 CG4698 RE26454
tracheal primordium;	
	FBgn0031745 CG8965 CG8965 GH01721
	FBgn0015903 apt CG5393 LD45581
large intestine specific anlage;	
	FBgn0040230 dbo CG6224 RE13447
longitudinal visceral mesoderm primordium;	
	FBgn0033992 CG11798 CG11798 LD23883
	FBgn0010453 Wnt4 CG4698 RE26454
external foregut primordium;	
	FBgn0031745 CG8965 CG8965 GH01721
	FBgn0015903 apt CG5393 LD45581
	FBgn0000577 en CG9015 LD16125
	FBgn0003346 Sd CG9999 LD16356
	FBgn0000492 Dr CG1897 LD04235
	FBgn0010453 Wnt4 CG4698 RE26454
	FBgn0000577 en CG9015 LD16125
procephalic neuroblasts;	
	FBgn0040271 Sulf1 CG6725 SD04414
	FBgn0040230 dbo CG6224 RE13447
	FBgn0002561 l(1)sc CG3839 RE59335
	FBgn0033992 CG11798 CG11798 LD23883
	FBgn0010453 Wnt4 CG4698 RE26454
lateral cord glia;	
	FBgn0002932 neur CG11988 LD45505
	FBgn0033992 CG11798 CG11798 LD23883
	FBgn0000611 exd CG8933 LD03509
	FBgn0000411 D CG5893 AT02631
	FBgn0038118 NULL CG7855 RE03551
	FBgn0052982 CG32982 CG32982 RE11317
	FBgn0029512 Aos1 CG12276 LD33652
	FBgn0017453 Zn72D CG5215 GH12756
	FBgn0015270 Orc2 CG3041 GH13824
embryonic small intestine;	
	FBgn0010453 Wnt4 CG4698 RE26454
head epidermis primordium;	
	FBgn0015903 apt CG5393 LD45581
	FBgn0003346 Sd CG9999 LD16356
embryonic inner optic lobe primordium;	
	FBgn0010453 Wnt4 CG4698 RE26454
salivary gland body primordium;	
	continued on next page...

Table 16 continued from previous page.

Anatomical Structures	Genes
	FBgn0000492 Dr CG1897 LD04235
embryonic brain;	
	FBgn0036494 Toll-6 CG7250 LD08841
	FBgn0037707 RnpS1 CG16788 LD23870
	FBgn0040271 Sulf1 CG6725 SD04414
	FBgn0040230 dbo CG6224 RE13447
	FBgn0003300 run CG1849 GH02614
	FBgn0015903 apt CG5393 LD45581
	FBgn0033992 CG11798 CG11798 LD23883
	FBgn0000611 exd CG8933 LD03509
	FBgn0000577 en CG9015 LD16125
	FBgn0038118 NULL CG7855 RE03551
	FBgn0010453 Wnt4 CG4698 RE26454
	FBgn0029512 Aos1 CG12276 LD33652
	FBgn0017453 Zn72D CG5215 GH12756
	FBgn0000577 en CG9015 LD16125
trunk mesoderm anlage;	
	FBgn0037707 RnpS1 CG16788 LD23870
	FBgn0031745 CG8965 CG8965 GH01721
	FBgn0000071 Ama CG2198 LD39923
	FBgn0002932 neur CG11988 LD45505
	FBgn0032130 CG3838 CG3838 LD21447
	FBgn0033992 CG11798 CG11798 LD23883
	FBgn0000611 exd CG8933 LD03509
	FBgn0000577 en CG9015 LD16125
	FBgn0003346 Sd CG9999 LD16356
	FBgn0000492 Dr CG1897 LD04235
	FBgn0015773 NetA CG18657 RE11206
	FBgn0052982 CG32982 CG32982 RE11317
	FBgn0000577 en CG9015 LD16125
sensory nervous system primordium;	
	FBgn0002932 neur CG11988 LD45505
	FBgn0010109 dpn CG8704 RE34993
	FBgn0003300 run CG1849 GH02614
	FBgn0000411 D CG5893 AT02631
	FBgn0017453 Zn72D CG5215 GH12756
anterior endoderm anlage;	
	FBgn0037707 RnpS1 CG16788 LD23870
	FBgn0002932 neur CG11988 LD45505
	FBgn0032130 CG3838 CG3838 LD21447
	FBgn0015903 apt CG5393 LD45581
	FBgn0000611 exd CG8933 LD03509
	FBgn0003346 Sd CG9999 LD16356
	FBgn0038434 CG14897 CG14897 RE60459
embryonic hindgut;	
	FBgn0031745 CG8965 CG8965 GH01721

continued on next page...

Table 16 continued from previous page.	
Anatomical Structures	Genes
	FBgn0040230 dbo CG6224 RE13447<br>FBgn0000411 D CG5893 AT02631<br>FBgn0010453 Wnt4 CG4698 RE26454<br>FBgn0017453 Zn72D CG5215 GH12756
embryonic Malpighian tubule;	
	FBgn0038118 NULL CG7855 RE03551<br>FBgn0012066 DNApol-delta CG5949 RE60511
ventral nerve cord;	
	FBgn0033802 CG17724 CG17724 LD12453<br>FBgn0037707 RnpS1 CG16788 LD23870<br>FBgn0002932 neur CG11988 LD45505<br>FBgn0040271 Sulf1 CG6725 SD04414<br>FBgn0040230 dbo CG6224 RE13447<br>FBgn0010109 dpn CG8704 RE34993<br>FBgn0003300 run CG1849 GH02614<br>FBgn0015903 apt CG5393 LD45581<br>FBgn0033992 CG11798 CG11798 LD23883<br>FBgn0000611 exd CG8933 LD03509<br>FBgn0000577 en CG9015 LD16125<br>FBgn0003346 Sd CG9999 LD16356<br>FBgn0000492 Dr CG1897 LD04235<br>FBgn0000411 D CG5893 AT02631<br>FBgn0052982 CG32982 CG32982 RE11317<br>FBgn0031878 CG9188 CG9188 RE16905<br>FBgn0010453 Wnt4 CG4698 RE26454<br>FBgn0030507 CG11164 CG11164 RE50056<br>FBgn0038434 CG14897 CG14897 RE60459<br>FBgn0012066 DNApol-delta CG5949 RE60511<br>FBgn0032906 CG9273 CG9273 RH07841<br>FBgn0029512 Aos1 CG12276 LD33652<br>FBgn0017453 Zn72D CG5215 GH12756<br>FBgn0015270 Orc2 CG3041 GH13824<br>FBgn0000577 en CG9015 LD16125
atrium;	
	FBgn0015903 apt CG5393 LD45581<br>FBgn0000577 en CG9015 LD16125<br>FBgn0000492 Dr CG1897 LD04235<br>FBgn0000577 en CG9015 LD16125
ventral nerve cord primordium;	
	FBgn0036494 Toll-6 CG7250 LD08841<br>FBgn0033802 CG17724 CG17724 LD12453<br>FBgn0037707 RnpS1 CG16788 LD23870<br>FBgn0031745 CG8965 CG8965 GH01721<br>FBgn0000071 Ama CG2198 LD39923<br>FBgn0002932 neur CG11988 LD45505<br>FBgn0040271 Sulf1 CG6725 SD04414

continued on next page...

Table 16 continued from previous page.

Anatomical Structures	Genes
	FBgn0040230 dbo CG6224 RE13447<br>FBgn0002561 l(1)sc CG3839 RE59335<br>FBgn0010109 dpn CG8704 RE34993<br>FBgn0003300 run CG1849 GH02614<br>FBgn0032130 CG3838 CG3838 LD21447<br>FBgn0015903 apt CG5393 LD45581<br>FBgn0033992 CG11798 CG11798 LD23883<br>FBgn0000611 exd CG8933 LD03509<br>FBgn0000577 en CG9015 LD16125<br>FBgn0003346 Sd CG9999 LD16356<br>FBgn0000492 Dr CG1897 LD04235<br>FBgn0000411 D CG5893 AT02631<br>FBgn0038118 NULL CG7855 RE03551<br>FBgn0015773 NetA CG18657 RE11206<br>FBgn0052982 CG32982 CG32982 RE11317<br>FBgn0015271 Orc5 CG7833 RE16687<br>FBgn0031878 CG9188 CG9188 RE16905<br>FBgn0010453 Wnt4 CG4698 RE26454<br>FBgn0030507 CG11164 CG11164 RE50056<br>FBgn0038434 CG14897 CG14897 RE60459<br>FBgn0012066 DNApol-delta CG5949 RE60511<br>FBgn0032906 CG9273 CG9273 RH07841<br>FBgn0010105 comm CG17943 RH64772<br>FBgn0029512 Aos1 CG12276 LD33652<br>FBgn0017453 Zn72D CG5215 GH12756<br>FBgn0015270 Orc2 CG3041 GH13824<br>FBgn0000577 en CG9015 LD16125
visual primordium;	FBgn0002932 neur CG11988 LD45505<br>FBgn0002561 l(1)sc CG3839 RE59335<br>FBgn0015903 apt CG5393 LD45581<br>FBgn0033992 CG11798 CG11798 LD23883<br>FBgn0000611 exd CG8933 LD03509<br>FBgn0003346 Sd CG9999 LD16356<br>FBgn0015271 Orc5 CG7833 RE16687<br>FBgn0010453 Wnt4 CG4698 RE26454<br>FBgn0012066 DNApol-delta CG5949 RE60511
lateral cord;	FBgn0036494 Toll-6 CG7250 LD08841<br>FBgn0033802 CG17724 CG17724 LD12453<br>FBgn0037707 RnpS1 CG16788 LD23870<br>FBgn0031745 CG8965 CG8965 GH01721<br>FBgn0000071 Ama CG2198 LD39923<br>FBgn0002932 neur CG11988 LD45505<br>FBgn0040271 Sulf1 CG6725 SD04414<br>FBgn0040230 dbo CG6224 RE13447

continued on next page...

Table 16 continued from previous page.	
Anatomical Structures	Genes
	FBgn0002561 l(1)sc CG3839 RE59335
	FBgn0010109 dpn CG8704 RE34993
	FBgn0003300 run CG1849 GH02614
	FBgn0032130 CG3838 CG3838 LD21447
	FBgn0015903 apt CG5393 LD45581
	FBgn0033992 CG11798 CG11798 LD23883
	FBgn0000611 exd CG8933 LD03509
	FBgn0000577 en CG9015 LD16125
	FBgn0003346 Sd CG9999 LD16356
	FBgn0000492 Dr CG1897 LD04235
	FBgn0000411 D CG5893 AT02631
	FBgn0038118 NULL CG7855 RE03551
	FBgn0015773 NetA CG18657 RE11206
	FBgn0052982 CG32982 CG32982 RE11317
	FBgn0015271 Orc5 CG7833 RE16687
	FBgn0031878 CG9188 CG9188 RE16905
	FBgn0010453 Wnt4 CG4698 RE26454
	FBgn0030507 CG11164 CG11164 RE50056
	FBgn0038434 CG14897 CG14897 RE60459
	FBgn0012066 DNApol-delta CG5949 RE60511
	FBgn0032906 CG9273 CG9273 RH07841
	FBgn0010105 comm CG17943 RH64772
	FBgn0029512 Aos1 CG12276 LD33652
	FBgn0017453 Zn72D CG5215 GH12756
	FBgn0015270 Orc2 CG3041 GH13824
	FBgn0000577 en CG9015 LD16125
foregut primordium;	
	FBgn0031745 CG8965 CG8965 GH01721
	FBgn0010453 Wnt4 CG4698 RE26454
embryonic leading edge cell specific anlage;	
	FBgn0010453 Wnt4 CG4698 RE26454
posterior endoderm anlage;	
	FBgn0037707 RnpS1 CG16788 LD23870
	FBgn0002932 neur CG11988 LD45505
	FBgn0002561 l(1)sc CG3839 RE59335
	FBgn0032130 CG3838 CG3838 LD21447
	FBgn0015903 apt CG5393 LD45581
	FBgn0000611 exd CG8933 LD03509
	FBgn0003346 Sd CG9999 LD16356
	FBgn0052982 CG32982 CG32982 RE11317
	FBgn0038434 CG14897 CG14897 RE60459
embryonic ventral epidermis;	
	FBgn0031745 CG8965 CG8965 GH01721
	FBgn0040230 dbo CG6224 RE13447
	FBgn0000411 D CG5893 AT02631
	FBgn0015773 NetA CG18657 RE11206

continued on next page...

Table 16 continued from previous page.

Anatomical Structures	Genes
	FBgn0052982 CG32982 CG32982 RE11317<br>FBgn0010105 comm CG17943 RH64772
lateral cord neuron;	FBgn0036494 Toll-6 CG7250 LD08841<br>FBgn0002932 neur CG11988 LD45505<br>FBgn0040271 Sulf1 CG6725 SD04414<br>FBgn0040230 dbo CG6224 RE13447<br>FBgn0033992 CG11798 CG11798 LD23883<br>FBgn0000611 exd CG8933 LD03509<br>FBgn0000577 en CG9015 LD16125<br>FBgn0000492 Dr CG1897 LD04235<br>FBgn0000411 D CG5893 AT02631<br>FBgn0038118 NULL CG7855 RE03551<br>FBgn0015773 NetA CG18657 RE11206<br>FBgn0052982 CG32982 CG32982 RE11317<br>FBgn0010453 Wnt4 CG4698 RE26454<br>FBgn0038434 CG14897 CG14897 RE60459<br>FBgn0032906 CG9273 CG9273 RH07841<br>FBgn0010105 comm CG17943 RH64772<br>FBgn0029512 Aos1 CG12276 LD33652<br>FBgn0017453 Zn72D CG5215 GH12756<br>FBgn0015270 Orc2 CG3041 GH13824<br>FBgn0000577 en CG9015 LD16125
mesectoderm anlage;	FBgn0037707 RnpS1 CG16788 LD23870<br>FBgn0031745 CG8965 CG8965 GH01721<br>FBgn0002932 neur CG11988 LD45505<br>FBgn0033992 CG11798 CG11798 LD23883<br>FBgn0000611 exd CG8933 LD03509<br>FBgn0000577 en CG9015 LD16125<br>FBgn0003346 Sd CG9999 LD16356<br>FBgn0000577 en CG9015 LD16125
visceral muscle primordium;	FBgn0037707 RnpS1 CG16788 LD23870<br>FBgn0031745 CG8965 CG8965 GH01721<br>FBgn0033992 CG11798 CG11798 LD23883<br>FBgn0015773 NetA CG18657 RE11206
amnioserosa;	FBgn0000071 Ama CG2198 LD39923
embryonic optic lobe;	FBgn0033992 CG11798 CG11798 LD23883<br>FBgn0010453 Wnt4 CG4698 RE26454
trunk mesoderm primordium;	FBgn0037707 RnpS1 CG16788 LD23870<br>FBgn0031745 CG8965 CG8965 GH01721<br>FBgn0000071 Ama CG2198 LD39923

continued on next page...

Table 16 continued from previous page.	
Anatomical Structures	Genes
	FBgn0002561 l(1)sc CG3839 RE59335<br>FBgn0033992 CG11798 CG11798 LD23883<br>FBgn0000611 exd CG8933 LD03509<br>FBgn0000577 en CG9015 LD16125<br>FBgn0003346 Sd CG9999 LD16356<br>FBgn0000492 Dr CG1897 LD04235<br>FBgn0015773 NetA CG18657 RE11206<br>FBgn0000577 en CG9015 LD16125
protocerebrum primordium;	FBgn0036494 Toll-6 CG7250 LD08841<br>FBgn0033802 CG17724 CG17724 LD12453<br>FBgn0037707 RnpS1 CG16788 LD23870<br>FBgn0002932 neur CG11988 LD45505<br>FBgn0040271 Sulf1 CG6725 SD04414<br>FBgn0040230 dbo CG6224 RE13447<br>FBgn0002561 l(1)sc CG3839 RE59335<br>FBgn0003300 run CG1849 GH02614<br>FBgn0032130 CG3838 CG3838 LD21447<br>FBgn0015903 apt CG5393 LD45581<br>FBgn0033992 CG11798 CG11798 LD23883<br>FBgn0000611 exd CG8933 LD03509<br>FBgn0000577 en CG9015 LD16125<br>FBgn0003346 Sd CG9999 LD16356<br>FBgn0038118 NULL CG7855 RE03551<br>FBgn0015773 NetA CG18657 RE11206<br>FBgn0015271 Orc5 CG7833 RE16687<br>FBgn0031878 CG9188 CG9188 RE16905<br>FBgn0010453 Wnt4 CG4698 RE26454<br>FBgn0030507 CG11164 CG11164 RE50056<br>FBgn0038434 CG14897 CG14897 RE60459<br>FBgn0012066 DNApol-delta CG5949 RE60511<br>FBgn0032906 CG9273 CG9273 RH07841<br>FBgn0029512 Aos1 CG12276 LD33652<br>FBgn0017453 Zn72D CG5215 GH12756<br>FBgn0015270 Orc2 CG3041 GH13824<br>FBgn0000577 en CG9015 LD16125
embryonic/larval pericardial cell;	FBgn0015903 apt CG5393 LD45581
embryonic dorsal pouch primordium;	FBgn0015903 apt CG5393 LD45581
embryonic/larval tracheal system;	FBgn0031745 CG8965 CG8965 GH01721<br>FBgn0000071 Ama CG2198 LD39923
ventral epidermis primordium;	FBgn0036494 Toll-6 CG7250 LD08841<br>FBgn0000071 Ama CG2198 LD39923
	continued on next page...

Table 16 continued from previous page.

Anatomical Structures	Genes
	FBgn0040230 dbo CG6224 RE13447
	FBgn0003300 run CG1849 GH02614
	FBgn0015903 apt CG5393 LD45581
	FBgn0033992 CG11798 CG11798 LD23883
	FBgn0000611 exd CG8933 LD03509
	FBgn0000577 en CG9015 LD16125
	FBgn0000492 Dr CG1897 LD04235
	FBgn0052982 CG32982 CG32982 RE11317
	FBgn0010453 Wnt4 CG4698 RE26454
	FBgn0000577 en CG9015 LD16125
neuroblasts of ventral nervous system;	
	FBgn0036494 Toll-6 CG7250 LD08841
	FBgn0002932 neur CG11988 LD45505
	FBgn0040271 Sulf1 CG6725 SD04414
	FBgn0040230 dbo CG6224 RE13447
	FBgn0002561 l(1)sc CG3839 RE59335
	FBgn0010109 dpn CG8704 RE34993
	FBgn0003300 run CG1849 GH02614
	FBgn0032130 CG3838 CG3838 LD21447
	FBgn0033992 CG11798 CG11798 LD23883
	FBgn0003346 Sd CG9999 LD16356
	FBgn0000411 D CG5893 AT02631
	FBgn0038118 NULL CG7855 RE03551
	FBgn0030507 CG11164 CG11164 RE50056
embryonic dorsal epidermis;	
	FBgn0033802 CG17724 CG17724 LD12453
	FBgn0000577 en CG9015 LD16125
	FBgn0000411 D CG5893 AT02631
	FBgn0015773 NetA CG18657 RE11206
	FBgn0052982 CG32982 CG32982 RE11317
	FBgn0000577 en CG9015 LD16125
embryonic hypocerebral ganglion;	
	FBgn0031745 CG8965 CG8965 GH01721
gnathal primordium;	
	FBgn0000071 Ama CG2198 LD39923
	FBgn0015903 apt CG5393 LD45581
	FBgn0000577 en CG9015 LD16125
	FBgn0000492 Dr CG1897 LD04235
	FBgn0010453 Wnt4 CG4698 RE26454
	FBgn0000577 en CG9015 LD16125
hindgut anlage;	
	FBgn0037707 RnpS1 CG16788 LD23870
	FBgn0031745 CG8965 CG8965 GH01721
	FBgn0040230 dbo CG6224 RE13447
	FBgn0010109 dpn CG8704 RE34993
	FBgn0003300 run CG1849 GH02614

continued on next page...

Table 16 continued from previous page.	
Anatomical Structures	Genes
	FBgn0015903 apt CG5393 LD45581
	FBgn0000611 exd CG8933 LD03509
	FBgn0000577 en CG9015 LD16125
	FBgn0003346 Sd CG9999 LD16356
	FBgn0000411 D CG5893 AT02631
	FBgn0000577 en CG9015 LD16125
clypeo-labral primordium;	
	FBgn0040230 dbo CG6224 RE13447
	FBgn0015903 apt CG5393 LD45581
	FBgn0000577 en CG9015 LD16125
	FBgn0010453 Wnt4 CG4698 RE26454
	FBgn0000577 en CG9015 LD16125
anterior midgut primordium;	
	FBgn0037707 RnpS1 CG16788 LD23870
	FBgn0000071 Ama CG2198 LD39923
	FBgn0017453 Zn72D CG5215 GH12756
head mesoderm P2 primordium;	
	FBgn0000071 Ama CG2198 LD39923
	FBgn0033992 CG11798 CG11798 LD23883
	FBgn0000611 exd CG8933 LD03509
	FBgn0003346 Sd CG9999 LD16356
	FBgn0015773 NetA CG18657 RE11206
	FBgn0052982 CG32982 CG32982 RE11317
cardiac mesoderm primordium;	
	FBgn0033992 CG11798 CG11798 LD23883
embryonic ganglion mother cell;	
	FBgn0002932 neur CG11988 LD45505
	FBgn0010453 Wnt4 CG4698 RE26454
	FBgn0030507 CG11164 CG11164 RE50056
lamellocyte specific anlage;	
	FBgn0052982 CG32982 CG32982 RE11317
embryonic large intestine;	
	FBgn0040230 dbo CG6224 RE13447
	FBgn0000577 en CG9015 LD16125
	FBgn0010453 Wnt4 CG4698 RE26454
	FBgn0000577 en CG9015 LD16125
