# Supplementary material for: Prediction of Gene Expression in Embryonic Structures of Drosophila melanogaster
Source: PLoS Comput Biol. 2007 Jul 20;3(7):e144. doi: 10.1371/journal.pcbi.0030144 (PMC1924873; doi:10.1371/journal.pcbi.0030144)
Supplement: Supplementary file 17 [file pcbi.0030144.st017.pdf]

Table 17: **Diversity of anatomical annotations of genes comprising functional unit aep2egut**: embryonic midgut && anterior midgut primordium && anterior endoderm primordium

Anatomical Structures	Genes
embryonic/larval oenocyte;	FBgn0040297 NHE2 CG9256 RE21674
germ cell;	FBgn0037811 CG12592 CG12592 AT09724
embryonic esophagus;	FBgn0039537 CG5590 CG5590 GH01709<br>FBgn0000659 fkh CG10002 RE03865
fat body/gonad primordium;	FBgn0037810 CG12819 CG12819 GM08848<br>FBgn0037607 CG8036 CG8036 HL01241<br>FBgn0014868 Ost48 CG9022 LD21941<br>FBgn0000257 car CG12230 LD22396<br>FBgn0011638 La CG10922 RE69138<br>FBgn0037811 CG12592 CG12592 AT09724<br>FBgn0034356 CG10924 CG10924 RE12569<br>FBgn0040297 NHE2 CG9256 RE21674
dorsal prothoracic pharyngeal muscle;	FBgn0038585 CG7993 CG7993 GH01229<br>FBgn0025140 pit CG6375 LD46167<br>FBgn0033562 CG6751 CG6751 RE59607<br>FBgn0032168 CG13126 CG13126 RE01590<br>FBgn0040260 Ugt36Bc CG17932 RE03785<br>FBgn0052920 CG32920 CG32920 RE19605<br>FBgn0033562 CG6751 CG6751 RE21021<br>FBgn0040297 NHE2 CG9256 RE21674<br>FBgn0032919 CG9253 CG9253 RE27528<br>FBgn0036774 mRpS26 CG7354 RE31184
embryonic epipharynx;	FBgn0039537 CG5590 CG5590 GH01709<br>FBgn0020647 KrT95D CG5405 AT03104
dorsal pharyngeal muscle primordium;	FBgn0010292 bys CG1430 LD14510<br>FBgn0025140 pit CG6375 LD46167<br>FBgn0033562 CG6751 CG6751 RE59607<br>FBgn0011638 La CG10922 RE69138<br>FBgn0032168 CG13126 CG13126 RE01590<br>FBgn0052920 CG32920 CG32920 RE19605<br>FBgn0033562 CG6751 CG6751 RE21021<br>FBgn0040297 NHE2 CG9256 RE21674<br>FBgn0040080 raps CG5692 RE22964<br>FBgn0032919 CG9253 CG9253 RE27528
ventral midline;	FBgn0020647 KrT95D CG5405 AT03104

continued on next page...

Table 17 continued from previous page.	
Anatomical Structures	Genes
	FBgn0000659 fkh CG10002 RE03865
gonad;	
	FBgn0033562 CG6751 CG6751 RE59607<br>FBgn0011638 La CG10922 RE69138<br>FBgn0037811 CG12592 CG12592 AT09724<br>FBgn0033562 CG6751 CG6751 RE21021<br>FBgn0040080 raps CG5692 RE22964<br>FBgn0032919 CG9253 CG9253 RE27528<br>FBgn0038636 CG7698 CG7698 RE31408
embryonic maxillary sensory complex;	
	FBgn0020647 KrT95D CG5405 AT03104
small intestine specific anlage;	
	FBgn0030406 CG1463 CG1463 GM05421<br>FBgn0000659 fkh CG10002 RE03865
embryonic midgut;	
	FBgn0053099 CG33099 CG33099 LD12611<br>FBgn0037810 CG12819 CG12819 GM08848<br>FBgn0010292 bys CG1430 LD14510<br>FBgn0031845 CG9232 CG9232 HL01018<br>FBgn0037607 CG8036 CG8036 HL01241<br>FBgn0012037 Ance CG8827 LD11258<br>FBgn0030406 CG1463 CG1463 GM05421<br>FBgn0010926 l(3)07882 CG5824 LD15947<br>FBgn0038391 GATAe CG10278 LD08432<br>FBgn0025366 Ip259 CG5277 GM13959<br>FBgn0033637 CG9006 CG9006 GM14222<br>FBgn0037707 RnpS1 CG16788 LD23870<br>FBgn0029629 CG8636 CG8636 LD24347<br>FBgn0038585 CG7993 CG7993 GH01229<br>FBgn0039537 CG5590 CG5590 GH01709<br>FBgn0025140 pit CG6375 LD46167<br>FBgn0027621 Pfrx CG3400 GH17337<br>FBgn0014868 Ost48 CG9022 LD21941<br>FBgn0000257 car CG12230 LD22396<br>FBgn0024364 EG:8D8.4 CG11417 LD23562<br>FBgn0037926 CG10535 CG10535 GM13228<br>FBgn0033562 CG6751 CG6751 RE59607<br>FBgn0011638 La CG10922 RE69138<br>FBgn0031229 CG3436 CG3436 GM13767<br>FBgn0032512 CG9305 CG9305 GH09630<br>FBgn0000054 Adf1 CG15845 LD09689<br>FBgn0038391 GATAe CG10278 LD08432<br>FBgn0020647 KrT95D CG5405 AT03104<br>FBgn0037811 CG12592 CG12592 AT09724<br>FBgn0032168 CG13126 CG13126 RE01590<br>FBgn0040260 Ugt36Bc CG17932 RE03785
	continued on next page...

Table 17 continued from previous page.

Anatomical Structures	Genes
procephalic ectoderm primordium;	FBgn0000659 fkh CG10002 RE03865<br>FBgn0051810 CG31810 CG31810 RE06583<br>FBgn0025631 EG:22E5.11 CG4322 RE06985<br>FBgn0035654 CG13290 CG13290 RE08107<br>FBgn0034356 CG10924 CG10924 RE12569<br>FBgn0037816 CG6345 CG6345 RE15838<br>FBgn0052920 CG32920 CG32920 RE19605<br>FBgn0033562 CG6751 CG6751 RE21021<br>FBgn0040297 NHE2 CG9256 RE21674<br>FBgn0040080 raps CG5692 RE22964<br>FBgn0032919 CG9253 CG9253 RE27528<br>FBgn0036774 mRpS26 CG7354 RE31184<br>FBgn0038636 CG7698 CG7698 RE31408<br>FBgn0041156 exex CG8254 RE39081<br>FBgn0051431 CG31431 CG31431 RE47547<br>FBgn0032194 CG4901 CG4901 RE48269<br>FBgn0032444 CG5525 CG5525 RE61939<br>FBgn0032195 CG4908 CG4908 RH68195<br>FBgn0036298 CG10627 CG10627 LD02044<br>FBgn0010926 l(3)07882 CG5824 LD15947<br>FBgn0027619 Adam CG12131 GH12681<br>FBgn0000054 Adf1 CG15845 LD09689<br>FBgn0012037 Ance CG8827 LD11258
embryonic proventriculus;	FBgn0037707 RnpS1 CG16788 LD23870<br>FBgn0039537 CG5590 CG5590 GH01709<br>FBgn0011638 La CG10922 RE69138<br>FBgn0037811 CG12592 CG12592 AT09724<br>FBgn0000659 fkh CG10002 RE03865<br>FBgn0040297 NHE2 CG9256 RE21674<br>FBgn0032919 CG9253 CG9253 RE27528<br>FBgn0038636 CG7698 CG7698 RE31408
dorsal ectoderm primordium;	FBgn0012037 Ance CG8827 LD11258<br>FBgn0033637 CG9006 CG9006 GM14222<br>FBgn0039537 CG5590 CG5590 GH01709<br>FBgn0025140 pit CG6375 LD46167<br>FBgn0014868 Ost48 CG9022 LD21941<br>FBgn0000659 fkh CG10002 RE03865<br>FBgn0036298 CG10627 CG10627 LD02044<br>FBgn0027619 Adam CG12131 GH12681<br>FBgn0012037 Ance CG8827 LD11258
dorsal epidermis primordium;	FBgn0027621 Pfrx CG3400 GH17337<br>FBgn0037811 CG12592 CG12592 AT09724

continued on next page...

Table 17 continued from previous page.

Anatomical Structures	Genes
	FBgn0037811 CG12592 CG12592 AT09724
Malpighian tubule primordium;	FBgn0030406 CG1463 CG1463 GM05421<br>FBgn0038391 GATAe CG10278 LD08432<br>FBgn0000659 fkh CG10002 RE03865<br>FBgn0037816 CG6345 CG6345 RE15838<br>FBgn0027619 Adam CG12131 GH12681
embryonic stomatogastric nervous system;	FBgn0000659 fkh CG10002 RE03865
embryonic/larval somatic muscle;	FBgn0025366 Ip259 CG5277 GM13959<br>FBgn0037707 RnpS1 CG16788 LD23870<br>FBgn0029629 CG8636 CG8636 LD24347<br>FBgn0038585 CG7993 CG7993 GH01229<br>FBgn0039537 CG5590 CG5590 GH01709<br>FBgn0025140 pit CG6375 LD46167<br>FBgn0024364 EG:8D8.4 CG11417 LD23562<br>FBgn0032168 CG13126 CG13126 RE01590<br>FBgn0033562 CG6751 CG6751 RE21021<br>FBgn0040297 NHE2 CG9256 RE21674<br>FBgn0032919 CG9253 CG9253 RE27528<br>FBgn0032444 CG5525 CG5525 RE61939
macrophage;	FBgn0040297 NHE2 CG9256 RE21674<br>FBgn0051431 CG31431 CG31431 RE47547
labral sensory complex primordium;	FBgn0035654 CG13290 CG13290 RE08107
embryonic central brain mushroom body;	FBgn0011638 La CG10922 RE69138<br>FBgn0037811 CG12592 CG12592 AT09724
embryonic salivary gland;	FBgn0014868 Ost48 CG9022 LD21941<br>FBgn0000659 fkh CG10002 RE03865<br>FBgn0051810 CG31810 CG31810 RE06583
ring gland;	FBgn0011638 La CG10922 RE69138<br>FBgn0033562 CG6751 CG6751 RE21021
mesectoderm primordium;	FBgn0027621 Pfrx CG3400 GH17337
embryonic gastric caecum;	FBgn0011638 La CG10922 RE69138<br>FBgn0032168 CG13126 CG13126 RE01590<br>FBgn0033562 CG6751 CG6751 RE21021
embryonic hypopharynx;	FBgn0039537 CG5590 CG5590 GH01709<br>FBgn0033562 CG6751 CG6751 RE21021

continued on next page...

Table 17 continued from previous page.

Anatomical Structures	Genes
anal pad specific anlage;	FBgn0010926 l(3)07882 CG5824 LD15947
embryonic/larval dorsal vessel;	FBgn0040297 NHE2 CG9256 RE21674
embryonic central brain;	FBgn0011638 La CG10922 RE69138<br>FBgn0031229 CG3436 CG3436 GM13767<br>FBgn0000659 fkh CG10002 RE03865
salivary gland body specific anlage;	FBgn0000659 fkh CG10002 RE03865
embryonic central brain neuron;	FBgn0037811 CG12592 CG12592 AT09724
ventral ectoderm primordium;	FBgn0027621 Pfrx CG3400 GH17337<br>FBgn0011638 La CG10922 RE69138<br>FBgn0037811 CG12592 CG12592 AT09724<br>FBgn0038636 CG7698 CG7698 RE31408
adult hindgut precursor;	FBgn0036298 CG10627 CG10627 LD02044
gonadal sheath;	FBgn0037810 CG12819 CG12819 GM08848
Malpighian tubule main body primordium;	FBgn0030406 CG1463 CG1463 GM05421<br>FBgn0000659 fkh CG10002 RE03865<br>FBgn0035654 CG13290 CG13290 RE08107
somatic muscle primordium;	FBgn0010292 bys CG1430 LD14510<br>FBgn0010926 l(3)07882 CG5824 LD15947<br>FBgn0037707 RnpS1 CG16788 LD23870<br>FBgn0038585 CG7993 CG7993 GH01229<br>FBgn0039537 CG5590 CG5590 GH01709<br>FBgn0032168 CG13126 CG13126 RE01590<br>FBgn0052920 CG32920 CG32920 RE19605<br>FBgn0033562 CG6751 CG6751 RE21021<br>FBgn0040297 NHE2 CG9256 RE21674<br>FBgn0040080 raps CG5692 RE22964<br>FBgn0032919 CG9253 CG9253 RE27528<br>FBgn0036298 CG10627 CG10627 LD02044<br>FBgn0010926 l(3)07882 CG5824 LD15947<br>FBgn0027619 Adam CG12131 GH12681
inclusive hindgut primordium;	FBgn0030406 CG1463 CG1463 GM05421<br>FBgn0025366 Ip259 CG5277 GM13959<br>FBgn0037707 RnpS1 CG16788 LD23870<br>FBgn0029629 CG8636 CG8636 LD24347<br>FBgn0038585 CG7993 CG7993 GH01229

continued on next page...

Table 17 continued from previous page.	
Anatomical Structures	Genes
	FBgn0014868 Ost48 CG9022 LD21941<br>FBgn0011638 La CG10922 RE69138<br>FBgn0038391 GATAe CG10278 LD08432<br>FBgn0037811 CG12592 CG12592 AT09724<br>FBgn0000659 fkh CG10002 RE03865<br>FBgn0035654 CG13290 CG13290 RE08107<br>FBgn0037816 CG6345 CG6345 RE15838<br>FBgn0052920 CG32920 CG32920 RE19605<br>FBgn0033562 CG6751 CG6751 RE21021<br>FBgn0038636 CG7698 CG7698 RE31408<br>FBgn0032444 CG5525 CG5525 RE61939<br>FBgn0036298 CG10627 CG10627 LD02044<br>FBgn0027619 Adam CG12131 GH12681
circular visceral muscle fibers;	
	FBgn0020647 KrT95D CG5405 AT03104
lateral cord surface glia;	
	FBgn0025631 EG:22E5.11 CG4322 RE06985
stomatogastric nervous system primordium;	
	FBgn0000659 fkh CG10002 RE03865
embryonic/larval muscle system;	
	FBgn0010926 l(3)07882 CG5824 LD15947<br>FBgn0025366 Ip259 CG5277 GM13959<br>FBgn0037707 RnpS1 CG16788 LD23870<br>FBgn0029629 CG8636 CG8636 LD24347<br>FBgn0038585 CG7993 CG7993 GH01229<br>FBgn0039537 CG5590 CG5590 GH01709<br>FBgn0025140 pit CG6375 LD46167<br>FBgn0024364 EG:8D8.4 CG11417 LD23562<br>FBgn0037926 CG10535 CG10535 GM13228<br>FBgn0011638 La CG10922 RE69138<br>FBgn0032168 CG13126 CG13126 RE01590<br>FBgn0052920 CG32920 CG32920 RE19605<br>FBgn0033562 CG6751 CG6751 RE21021<br>FBgn0040297 NHE2 CG9256 RE21674<br>FBgn0040080 raps CG5692 RE22964<br>FBgn0032919 CG9253 CG9253 RE27528<br>FBgn0036774 mRpS26 CG7354 RE31184<br>FBgn0032444 CG5525 CG5525 RE61939<br>FBgn0036298 CG10627 CG10627 LD02044<br>FBgn0010926 l(3)07882 CG5824 LD15947<br>FBgn0027619 Adam CG12131 GH12681
anterior endoderm primordium;	
	FBgn0053099 CG33099 CG33099 LD12611<br>FBgn0037810 CG12819 CG12819 GM08848<br>FBgn0010292 bys CG1430 LD14510<br>FBgn0031845 CG9232 CG9232 HL01018
	continued on next page...

Table 17 continued from previous page.	
Anatomical Structures	Genes
	FBgn0037607 CG8036 CG8036 HL01241<br>FBgn0012037 Ance CG8827 LD11258<br>FBgn0030406 CG1463 CG1463 GM05421<br>FBgn0010926 l(3)07882 CG5824 LD15947<br>FBgn0038391 GATAe CG10278 LD08432<br>FBgn0025366 Ip259 CG5277 GM13959<br>FBgn0033637 CG9006 CG9006 GM14222<br>FBgn0037707 RnpS1 CG16788 LD23870<br>FBgn0029629 CG8636 CG8636 LD24347<br>FBgn0038585 CG7993 CG7993 GH01229<br>FBgn0039537 CG5590 CG5590 GH01709<br>FBgn0025140 pit CG6375 LD46167<br>FBgn0027621 Pfrx CG3400 GH17337<br>FBgn0014868 Ost48 CG9022 LD21941<br>FBgn0000257 car CG12230 LD22396<br>FBgn0024364 EG:8D8.4 CG11417 LD23562<br>FBgn0037926 CG10535 CG10535 GM13228<br>FBgn0033562 CG6751 CG6751 RE59607<br>FBgn0011638 La CG10922 RE69138<br>FBgn0031229 CG3436 CG3436 GM13767<br>FBgn0032512 CG9305 CG9305 GH09630<br>FBgn0000054 Adf1 CG15845 LD09689<br>FBgn0038391 GATAe CG10278 LD08432<br>FBgn0020647 KrT95D CG5405 AT03104<br>FBgn0037811 CG12592 CG12592 AT09724<br>FBgn0032168 CG13126 CG13126 RE01590<br>FBgn0040260 Ugt36Bc CG17932 RE03785<br>FBgn0000659 fkh CG10002 RE03865<br>FBgn0051810 CG31810 CG31810 RE06583<br>FBgn0025631 EG:22E5.11 CG4322 RE06985<br>FBgn0035654 CG13290 CG13290 RE08107<br>FBgn0034356 CG10924 CG10924 RE12569<br>FBgn0037816 CG6345 CG6345 RE15838<br>FBgn0052920 CG32920 CG32920 RE19605<br>FBgn0033562 CG6751 CG6751 RE21021<br>FBgn0040297 NHE2 CG9256 RE21674<br>FBgn0040080 raps CG5692 RE22964<br>FBgn0032919 CG9253 CG9253 RE27528<br>FBgn0036774 mRpS26 CG7354 RE31184<br>FBgn0038636 CG7698 CG7698 RE31408<br>FBgn0041156 exex CG8254 RE39081<br>FBgn0051431 CG31431 CG31431 RE47547<br>FBgn0032194 CG4901 CG4901 RE48269<br>FBgn0032444 CG5525 CG5525 RE61939<br>FBgn0032195 CG4908 CG4908 RH68195<br>FBgn0036298 CG10627 CG10627 LD02044
	continued on next page...

Table 17 continued from previous page.	
Anatomical Structures	Genes
	FBgn0010926 l(3)07882 CG5824 LD15947
	FBgn0027619 Adam CG12131 GH12681
	FBgn0000054 Adf1 CG15845 LD09689
	FBgn0012037 Ance CG8827 LD11258
embryonic rectum;	
	FBgn0000659 fkh CG10002 RE03865
posterior midgut primordium;	
	FBgn0037810 CG12819 CG12819 GM08848
	FBgn0010292 bys CG1430 LD14510
	FBgn0031845 CG9232 CG9232 HL01018
	FBgn0037607 CG8036 CG8036 HL01241
	FBgn0012037 Ance CG8827 LD11258
	FBgn0030406 CG1463 CG1463 GM05421
	FBgn0010926 l(3)07882 CG5824 LD15947
	FBgn0038391 GATAe CG10278 LD08432
	FBgn0025366 Ip259 CG5277 GM13959
	FBgn0033637 CG9006 CG9006 GM14222
	FBgn0037707 RnpS1 CG16788 LD23870
	FBgn0029629 CG8636 CG8636 LD24347
	FBgn0038585 CG7993 CG7993 GH01229
	FBgn0039537 CG5590 CG5590 GH01709
	FBgn0025140 pit CG6375 LD46167
	FBgn0027621 Pfrx CG3400 GH17337
	FBgn0014868 Ost48 CG9022 LD21941
	FBgn0000257 car CG12230 LD22396
	FBgn0024364 EG:8D8.4 CG11417 LD23562
	FBgn0037926 CG10535 CG10535 GM13228
	FBgn0033562 CG6751 CG6751 RE59607
	FBgn0011638 La CG10922 RE69138
	FBgn0031229 CG3436 CG3436 GM13767
	FBgn0032512 CG9305 CG9305 GH09630
	FBgn0000054 Adf1 CG15845 LD09689
	FBgn0038391 GATAe CG10278 LD08432
	FBgn0020647 KrT95D CG5405 AT03104
	FBgn0037811 CG12592 CG12592 AT09724
	FBgn0032168 CG13126 CG13126 RE01590
	FBgn0040260 Ugt36Bc CG17932 RE03785
	FBgn0000659 fkh CG10002 RE03865
	FBgn0051810 CG31810 CG31810 RE06583
	FBgn0025631 EG:22E5.11 CG4322 RE06985
	FBgn0035654 CG13290 CG13290 RE08107
	FBgn0034356 CG10924 CG10924 RE12569
	FBgn0037816 CG6345 CG6345 RE15838
	FBgn0052920 CG32920 CG32920 RE19605
	FBgn0033562 CG6751 CG6751 RE21021
	FBgn0040297 NHE2 CG9256 RE21674
	continued on next page...

Table 17 continued from previous page.	
Anatomical Structures	Genes
posterior endoderm primordium;	FBgn0040080 raps CG5692 RE22964<br>FBgn0032919 CG9253 CG9253 RE27528<br>FBgn0036774 mRpS26 CG7354 RE31184<br>FBgn0038636 CG7698 CG7698 RE31408<br>FBgn0041156 exex CG8254 RE39081<br>FBgn0032194 CG4901 CG4901 RE48269<br>FBgn0032444 CG5525 CG5525 RE61939<br>FBgn0032195 CG4908 CG4908 RH68195<br>FBgn0036298 CG10627 CG10627 LD02044<br>FBgn0010926 l(3)07882 CG5824 LD15947<br>FBgn0027619 Adam CG12131 GH12681<br>FBgn0000054 Adf1 CG15845 LD09689<br>FBgn0012037 Ance CG8827 LD11258<br><br>FBgn0053099 CG33099 CG33099 LD12611<br>FBgn0037810 CG12819 CG12819 GM08848<br>FBgn0010292 bys CG1430 LD14510<br>FBgn0031845 CG9232 CG9232 HL01018<br>FBgn0037607 CG8036 CG8036 HL01241<br>FBgn0012037 Ance CG8827 LD11258<br>FBgn0030406 CG1463 CG1463 GM05421<br>FBgn0010926 l(3)07882 CG5824 LD15947<br>FBgn0038391 GATAe CG10278 LD08432<br>FBgn0025366 Ip259 CG5277 GM13959<br>FBgn0033637 CG9006 CG9006 GM14222<br>FBgn0037707 RnpS1 CG16788 LD23870<br>FBgn0029629 CG8636 CG8636 LD24347<br>FBgn0038585 CG7993 CG7993 GH01229<br>FBgn0039537 CG5590 CG5590 GH01709<br>FBgn0025140 pit CG6375 LD46167<br>FBgn0027621 Pfrx CG3400 GH17337<br>FBgn0014868 Ost48 CG9022 LD21941<br>FBgn0000257 car CG12230 LD22396<br>FBgn0024364 EG:8D8.4 CG11417 LD23562<br>FBgn0037926 CG10535 CG10535 GM13228<br>FBgn0033562 CG6751 CG6751 RE59607<br>FBgn0011638 La CG10922 RE69138<br>FBgn0031229 CG3436 CG3436 GM13767<br>FBgn0032512 CG9305 CG9305 GH09630<br>FBgn0000054 Adf1 CG15845 LD09689<br>FBgn0038391 GATAe CG10278 LD08432<br>FBgn0020647 KrT95D CG5405 AT03104<br>FBgn0037811 CG12592 CG12592 AT09724<br>FBgn0032168 CG13126 CG13126 RE01590<br>FBgn0040260 Ugt36Bc CG17932 RE03785<br>FBgn0000659 fkh CG10002 RE03865
	continued on next page...

Table 17 continued from previous page.	
Anatomical Structures	Genes
	FBgn0051810 CG31810 CG31810 RE06583<br>FBgn0025631 EG:22E5.11 CG4322 RE06985<br>FBgn0035654 CG13290 CG13290 RE08107<br>FBgn0034356 CG10924 CG10924 RE12569<br>FBgn0037816 CG6345 CG6345 RE15838<br>FBgn0052920 CG32920 CG32920 RE19605<br>FBgn0033562 CG6751 CG6751 RE21021<br>FBgn0040297 NHE2 CG9256 RE21674<br>FBgn0040080 raps CG5692 RE22964<br>FBgn0032919 CG9253 CG9253 RE27528<br>FBgn0036774 mRpS26 CG7354 RE31184<br>FBgn0038636 CG7698 CG7698 RE31408<br>FBgn0041156 exex CG8254 RE39081<br>FBgn0051431 CG31431 CG31431 RE47547<br>FBgn0032194 CG4901 CG4901 RE48269<br>FBgn0032444 CG5525 CG5525 RE61939<br>FBgn0032195 CG4908 CG4908 RH68195<br>FBgn0036298 CG10627 CG10627 LD02044<br>FBgn0010926 l(3)07882 CG5824 LD15947<br>FBgn0027619 Adam CG12131 GH12681<br>FBgn0000054 Adf1 CG15845 LD09689<br>FBgn0012037 Ance CG8827 LD11258
fat body specific anlage;	FBgn0000257 car CG12230 LD22396<br>FBgn0024364 EG:8D8.4 CG11417 LD23562<br>FBgn0033562 CG6751 CG6751 RE59607<br>FBgn0027619 Adam CG12131 GH12681
embryonic central brain glia;	FBgn0037811 CG12592 CG12592 AT09724
prothoracic gland;	FBgn0012037 Ance CG8827 LD11258<br>FBgn0012037 Ance CG8827 LD11258
midline primordium;	FBgn0000659 fkh CG10002 RE03865
embryonic/larval fat body;	FBgn0037607 CG8036 CG8036 HL01241<br>FBgn0033637 CG9006 CG9006 GM14222<br>FBgn0014868 Ost48 CG9022 LD21941<br>FBgn0000257 car CG12230 LD22396<br>FBgn0034356 CG10924 CG10924 RE12569<br>FBgn0040297 NHE2 CG9256 RE21674
hindgut proper primordium;	FBgn0025366 Ip259 CG5277 GM13959<br>FBgn0033637 CG9006 CG9006 GM14222<br>FBgn0037707 RnpS1 CG16788 LD23870<br>FBgn0014868 Ost48 CG9022 LD21941
	continued on next page...

Table 17 continued from previous page.

Anatomical Structures	Genes
	FBgn0024364 EG:8D8.4 CG11417 LD23562
	FBgn0011638 La CG10922 RE69138
	FBgn0037811 CG12592 CG12592 AT09724
	FBgn0000659 fkh CG10002 RE03865
	FBgn0052920 CG32920 CG32920 RE19605
	FBgn0038636 CG7698 CG7698 RE31408
	FBgn0032444 CG5525 CG5525 RE61939
	FBgn0010926 l(3)07882 CG5824 LD15947
embryonic/larval visceral muscle;	
	FBgn0025366 Ip259 CG5277 GM13959
	FBgn0037707 RnpS1 CG16788 LD23870
	FBgn0029629 CG8636 CG8636 LD24347
	FBgn0011638 La CG10922 RE69138
	FBgn0020647 KrT95D CG5405 AT03104
	FBgn0032168 CG13126 CG13126 RE01590
	FBgn0032919 CG9253 CG9253 RE27528
	FBgn0036774 mRpS26 CG7354 RE31184
embryonic dorsal pouch;	
	FBgn0012037 Ance CG8827 LD11258
	FBgn0012037 Ance CG8827 LD11258
labral sensory complex;	
	FBgn0035654 CG13290 CG13290 RE08107
embryonic head epidermis;	
	FBgn0012037 Ance CG8827 LD11258
	FBgn0000659 fkh CG10002 RE03865
	FBgn0012037 Ance CG8827 LD11258
lymph gland;	
	FBgn0012037 Ance CG8827 LD11258
	FBgn0037811 CG12592 CG12592 AT09724
	FBgn0040080 raps CG5692 RE22964
	FBgn0032919 CG9253 CG9253 RE27528
	FBgn0012037 Ance CG8827 LD11258
proventriculus primordium;	
	FBgn0012037 Ance CG8827 LD11258
	FBgn0030406 CG1463 CG1463 GM05421
	FBgn0037707 RnpS1 CG16788 LD23870
	FBgn0025140 pit CG6375 LD46167
	FBgn0024364 EG:8D8.4 CG11417 LD23562
	FBgn0038391 GATAe CG10278 LD08432
	FBgn0000659 fkh CG10002 RE03865
	FBgn0012037 Ance CG8827 LD11258
embryonic central brain surface glia;	
	FBgn0025631 EG:22E5.11 CG4322 RE06985
embryonic midgut chamber;	
	FBgn0038391 GATAe CG10278 LD08432
	FBgn0032512 CG9305 CG9305 GH09630

continued on next page...

Table 17 continued from previous page.	
Anatomical Structures	Genes
	FBgn0033562 CG6751 CG6751 RE21021<br>FBgn0041156 exex CG8254 RE39081<br>FBgn0051431 CG31431 CG31431 RE47547<br>FBgn0032195 CG4908 CG4908 RH68195
embryonic main segment of Malpighian tubule;	FBgn0000659 fkh CG10002 RE03865
embryonic central nervous system;	FBgn0037607 CG8036 CG8036 HL01241<br>FBgn0037707 RnpS1 CG16788 LD23870<br>FBgn0011638 La CG10922 RE69138<br>FBgn0000054 Adf1 CG15845 LD09689<br>FBgn0037811 CG12592 CG12592 AT09724<br>FBgn0000659 fkh CG10002 RE03865<br>FBgn0025631 EG:22E5.11 CG4322 RE06985<br>FBgn0041156 exex CG8254 RE39081<br>FBgn0000054 Adf1 CG15845 LD09689
embryonic foregut;	FBgn0039537 CG5590 CG5590 GH01709<br>FBgn0000659 fkh CG10002 RE03865<br>FBgn0040297 NHE2 CG9256 RE21674
tracheal primordium;	FBgn0037811 CG12592 CG12592 AT09724
large intestine specific anlage;	FBgn0000659 fkh CG10002 RE03865
longitudinal visceral mesoderm primordium;	FBgn0033562 CG6751 CG6751 RE21021<br>FBgn0032919 CG9253 CG9253 RE27528<br>FBgn0036298 CG10627 CG10627 LD02044
external foregut primordium;	FBgn0029629 CG8636 CG8636 LD24347<br>FBgn0014868 Ost48 CG9022 LD21941<br>FBgn0037811 CG12592 CG12592 AT09724<br>FBgn0000659 fkh CG10002 RE03865<br>FBgn0033562 CG6751 CG6751 RE21021
embryonic proventriculus outer layer;	FBgn0014868 Ost48 CG9022 LD21941
lateral cord glia;	FBgn0020647 KrT95D CG5405 AT03104<br>FBgn0037811 CG12592 CG12592 AT09724
procephalic neuroblasts;	FBgn0037607 CG8036 CG8036 HL01241<br>FBgn0031229 CG3436 CG3436 GM13767
rectum specific anlage;	FBgn0000659 fkh CG10002 RE03865
embryonic small intestine;	FBgn0011638 La CG10922 RE69138
	continued on next page...

Table 17 continued from previous page.

Anatomical Structures	Genes
	FBgn0000659 fkh CG10002 RE03865
procrystal cell;	
	FBgn0027621 Pfrx CG3400 GH17337<br>FBgn0040260 Ugt36Bc CG17932 RE03785<br>FBgn0051431 CG31431 CG31431 RE47547
garland cell primordium;	
	FBgn0000257 car CG12230 LD22396<br>FBgn0040297 NHE2 CG9256 RE21674
embryonic brain;	
	FBgn0037607 CG8036 CG8036 HL01241<br>FBgn0030406 CG1463 CG1463 GM05421<br>FBgn0037707 RnpS1 CG16788 LD23870<br>FBgn0014868 Ost48 CG9022 LD21941<br>FBgn0011638 La CG10922 RE69138<br>FBgn0000659 fkh CG10002 RE03865<br>FBgn0025631 EG:22E5.11 CG4322 RE06985
salivary gland body primordium;	
	FBgn0030406 CG1463 CG1463 GM05421<br>FBgn0014868 Ost48 CG9022 LD21941<br>FBgn0024364 EG:8D8.4 CG11417 LD23562<br>FBgn0037926 CG10535 CG10535 GM13228<br>FBgn0000659 fkh CG10002 RE03865<br>FBgn0051810 CG31810 CG31810 RE06583<br>FBgn0033562 CG6751 CG6751 RE21021<br>FBgn0040080 raps CG5692 RE22964<br>FBgn0032919 CG9253 CG9253 RE27528<br>FBgn0036298 CG10627 CG10627 LD02044<br>FBgn0027619 Adam CG12131 GH12681
embryonic hindgut;	
	FBgn0031845 CG9232 CG9232 HL01018<br>FBgn0033637 CG9006 CG9006 GM14222<br>FBgn0029629 CG8636 CG8636 LD24347<br>FBgn0039537 CG5590 CG5590 GH01709<br>FBgn0014868 Ost48 CG9022 LD21941<br>FBgn0011638 La CG10922 RE69138<br>FBgn0037811 CG12592 CG12592 AT09724<br>FBgn0000659 fkh CG10002 RE03865<br>FBgn0052920 CG32920 CG32920 RE19605<br>FBgn0032444 CG5525 CG5525 RE61939<br>FBgn0036298 CG10627 CG10627 LD02044<br>FBgn0010926 l(3)07882 CG5824 LD15947<br>FBgn0027619 Adam CG12131 GH12681
embryonic anal pad;	
	FBgn0000659 fkh CG10002 RE03865<br>FBgn0010926 l(3)07882 CG5824 LD15947
embryonic Malpighian tubule;	

continued on next page...

Table 17 continued from previous page.

Anatomical Structures	Genes
	FBgn0038391 GATAe CG10278 LD08432<br>FBgn0033637 CG9006 CG9006 GM14222<br>FBgn0011638 La CG10922 RE69138<br>FBgn0038391 GATAe CG10278 LD08432<br>FBgn0000659 fkh CG10002 RE03865<br>FBgn0035654 CG13290 CG13290 RE08107<br>FBgn0037816 CG6345 CG6345 RE15838<br>FBgn0036298 CG10627 CG10627 LD02044<br>FBgn0027619 Adam CG12131 GH12681
ventral nerve cord;	
	FBgn0037707 RnpS1 CG16788 LD23870<br>FBgn0014868 Ost48 CG9022 LD21941<br>FBgn0031229 CG3436 CG3436 GM13767<br>FBgn0000054 Adf1 CG15845 LD09689<br>FBgn0037811 CG12592 CG12592 AT09724<br>FBgn0025631 EG:22E5.11 CG4322 RE06985<br>FBgn0041156 exex CG8254 RE39081<br>FBgn0000054 Adf1 CG15845 LD09689
ventral nerve cord primordium;	
	FBgn0037607 CG8036 CG8036 HL01241<br>FBgn0037707 RnpS1 CG16788 LD23870<br>FBgn0039537 CG5590 CG5590 GH01709<br>FBgn0027621 Pfrx CG3400 GH17337<br>FBgn0037811 CG12592 CG12592 AT09724<br>FBgn0040297 NHE2 CG9256 RE21674
visual primordium;	
	FBgn0038636 CG7698 CG7698 RE31408
foregut primordium;	
	FBgn0000659 fkh CG10002 RE03865<br>FBgn0033562 CG6751 CG6751 RE21021
lateral cord;	
	FBgn0037607 CG8036 CG8036 HL01241<br>FBgn0037707 RnpS1 CG16788 LD23870<br>FBgn0014868 Ost48 CG9022 LD21941<br>FBgn0040297 NHE2 CG9256 RE21674<br>FBgn0041156 exex CG8254 RE39081
crystal cell;	
	FBgn0027621 Pfrx CG3400 GH17337<br>FBgn0040260 Ugt36Bc CG17932 RE03785<br>FBgn0051431 CG31431 CG31431 RE47547
embryonic ventral epidermis;	
	FBgn0035654 CG13290 CG13290 RE08107
lateral cord neuron;	
	FBgn0037811 CG12592 CG12592 AT09724<br>FBgn0041156 exex CG8254 RE39081
embryonic midgut interstitial cell;	

continued on next page...

Table 17 continued from previous page.

Anatomical Structures	Genes
	FBgn0032195 CG4908 CG4908 RH68195
visceral muscle primordium;	FBgn0010926 l(3)07882 CG5824 LD15947
	FBgn0037707 RnpS1 CG16788 LD23870
	FBgn0011638 La CG10922 RE69138
	FBgn0020647 KrT95D CG5405 AT03104
	FBgn0033562 CG6751 CG6751 RE21021
	FBgn0032919 CG9253 CG9253 RE27528
	FBgn0010926 l(3)07882 CG5824 LD15947
amnioserosa;	FBgn0053099 CG33099 CG33099 LD12611
	FBgn0031845 CG9232 CG9232 HL01018
	FBgn0012037 Ance CG8827 LD11258
	FBgn0000659 fkh CG10002 RE03865
	FBgn0034356 CG10924 CG10924 RE12569
	FBgn0051431 CG31431 CG31431 RE47547
	FBgn0012037 Ance CG8827 LD11258
embryonic salivary gland duct;	FBgn0039537 CG5590 CG5590 GH01709
trunk mesoderm primordium;	FBgn0053099 CG33099 CG33099 LD12611
	FBgn0037810 CG12819 CG12819 GM08848
	FBgn0010292 bys CG1430 LD14510
	FBgn0030406 CG1463 CG1463 GM05421
	FBgn0010926 l(3)07882 CG5824 LD15947
	FBgn0025366 Ip259 CG5277 GM13959
	FBgn0033637 CG9006 CG9006 GM14222
	FBgn0037707 RnpS1 CG16788 LD23870
	FBgn0038585 CG7993 CG7993 GH01229
	FBgn0039537 CG5590 CG5590 GH01709
	FBgn0025140 pit CG6375 LD46167
	FBgn0027621 Pfrx CG3400 GH17337
	FBgn0014868 Ost48 CG9022 LD21941
	FBgn0024364 EG:8D8.4 CG11417 LD23562
	FBgn0037926 CG10535 CG10535 GM13228
	FBgn0033562 CG6751 CG6751 RE59607
	FBgn0011638 La CG10922 RE69138
	FBgn0000054 Adf1 CG15845 LD09689
	FBgn0032168 CG13126 CG13126 RE01590
	FBgn0052920 CG32920 CG32920 RE19605
	FBgn0033562 CG6751 CG6751 RE21021
	FBgn0040297 NHE2 CG9256 RE21674
	FBgn0040080 raps CG5692 RE22964
	FBgn0032919 CG9253 CG9253 RE27528
	FBgn0038636 CG7698 CG7698 RE31408
	FBgn0032444 CG5525 CG5525 RE61939

continued on next page...

Table 17 continued from previous page.	
Anatomical Structures	Genes
	FBgn0036298 CG10627 CG10627 LD02044<br>FBgn0010926 l(3)07882 CG5824 LD15947<br>FBgn0027619 Adam CG12131 GH12681<br>FBgn0000054 Adf1 CG15845 LD09689
protocerebrum primordium;	
	FBgn0037707 RnpS1 CG16788 LD23870<br>FBgn0037811 CG12592 CG12592 AT09724<br>FBgn0000659 fkh CG10002 RE03865<br>FBgn0040297 NHE2 CG9256 RE21674<br>FBgn0032919 CG9253 CG9253 RE27528<br>FBgn0038636 CG7698 CG7698 RE31408
embryonic dorsal pouch primordium;	
	FBgn0012037 Ance CG8827 LD11258<br>FBgn0012037 Ance CG8827 LD11258
embryonic/larval garland cell;	
	FBgn0000257 car CG12230 LD22396<br>FBgn0040297 NHE2 CG9256 RE21674
ventral epidermis primordium;	
	FBgn0037811 CG12592 CG12592 AT09724
neuroblasts of ventral nervous system;	
	FBgn0037607 CG8036 CG8036 HL01241<br>FBgn0031229 CG3436 CG3436 GM13767<br>FBgn0040297 NHE2 CG9256 RE21674
gnathal primordium;	
	FBgn0035654 CG13290 CG13290 RE08107
embryonic/larval hemocyte;	
	FBgn0040297 NHE2 CG9256 RE21674<br>FBgn0051431 CG31431 CG31431 RE47547
anterior midgut primordium;	
	FBgn0053099 CG33099 CG33099 LD12611<br>FBgn0037810 CG12819 CG12819 GM08848<br>FBgn0010292 bys CG1430 LD14510<br>FBgn0031845 CG9232 CG9232 HL01018<br>FBgn0037607 CG8036 CG8036 HL01241<br>FBgn0012037 Ance CG8827 LD11258<br>FBgn0030406 CG1463 CG1463 GM05421<br>FBgn0010926 l(3)07882 CG5824 LD15947<br>FBgn0038391 GATAe CG10278 LD08432<br>FBgn0025366 Ip259 CG5277 GM13959<br>FBgn0033637 CG9006 CG9006 GM14222<br>FBgn0037707 RnpS1 CG16788 LD23870<br>FBgn0029629 CG8636 CG8636 LD24347<br>FBgn0038585 CG7993 CG7993 GH01229<br>FBgn0039537 CG5590 CG5590 GH01709<br>FBgn0025140 pit CG6375 LD46167<br>FBgn0027621 Pfrx CG3400 GH17337
	continued on next page...

Table 17 continued from previous page.	
Anatomical Structures	Genes
	FBgn0014868 Ost48 CG9022 LD21941
	FBgn0000257 car CG12230 LD22396
	FBgn0024364 EG:8D8.4 CG11417 LD23562
	FBgn0037926 CG10535 CG10535 GM13228
	FBgn0033562 CG6751 CG6751 RE59607
	FBgn0011638 La CG10922 RE69138
	FBgn0031229 CG3436 CG3436 GM13767
	FBgn0032512 CG9305 CG9305 GH09630
	FBgn0000054 Adf1 CG15845 LD09689
	FBgn0038391 GATAe CG10278 LD08432
	FBgn0020647 KrT95D CG5405 AT03104
	FBgn0037811 CG12592 CG12592 AT09724
	FBgn0032168 CG13126 CG13126 RE01590
	FBgn0040260 Ugt36Bc CG17932 RE03785
	FBgn0000659 fkh CG10002 RE03865
	FBgn0051810 CG31810 CG31810 RE06583
	FBgn0025631 EG:22E5.11 CG4322 RE06985
	FBgn0035654 CG13290 CG13290 RE08107
	FBgn0034356 CG10924 CG10924 RE12569
	FBgn0037816 CG6345 CG6345 RE15838
	FBgn0052920 CG32920 CG32920 RE19605
	FBgn0033562 CG6751 CG6751 RE21021
	FBgn0040297 NHE2 CG9256 RE21674
	FBgn0040080 raps CG5692 RE22964
	FBgn0032919 CG9253 CG9253 RE27528
	FBgn0036774 mRpS26 CG7354 RE31184
	FBgn0038636 CG7698 CG7698 RE31408
	FBgn0041156 exex CG8254 RE39081
	FBgn0051431 CG31431 CG31431 RE47547
	FBgn0032194 CG4901 CG4901 RE48269
	FBgn0032444 CG5525 CG5525 RE61939
	FBgn0032195 CG4908 CG4908 RH68195
	FBgn0036298 CG10627 CG10627 LD02044
	FBgn0010926 l(3)07882 CG5824 LD15947
	FBgn0027619 Adam CG12131 GH12681
	FBgn0000054 Adf1 CG15845 LD09689
	FBgn0012037 Ance CG8827 LD11258
head mesoderm P2 primordium;	
	FBgn0010292 bys CG1430 LD14510
	FBgn0010926 l(3)07882 CG5824 LD15947
	FBgn0025366 Ip259 CG5277 GM13959
	FBgn0033637 CG9006 CG9006 GM14222
	FBgn0039537 CG5590 CG5590 GH01709
	FBgn0025140 pit CG6375 LD46167
	FBgn0027621 Pfrx CG3400 GH17337
	FBgn0014868 Ost48 CG9022 LD21941
	continued on next page...

Table 17 continued from previous page.	
Anatomical Structures	Genes
	FBgn0000257 car CG12230 LD22396
	FBgn0024364 EG:8D8.4 CG11417 LD23562
	FBgn0033562 CG6751 CG6751 RE59607
	FBgn0011638 La CG10922 RE69138
	FBgn0000054 Adf1 CG15845 LD09689
	FBgn0040260 Ugt36Bc CG17932 RE03785
	FBgn0025631 EG:22E5.11 CG4322 RE06985
	FBgn0052920 CG32920 CG32920 RE19605
	FBgn0033562 CG6751 CG6751 RE21021
	FBgn0040297 NHE2 CG9256 RE21674
	FBgn0040080 raps CG5692 RE22964
	FBgn0032919 CG9253 CG9253 RE27528
	FBgn0051431 CG31431 CG31431 RE47547
	FBgn0032444 CG5525 CG5525 RE61939
	FBgn0036298 CG10627 CG10627 LD02044
	FBgn0010926 l(3)07882 CG5824 LD15947
	FBgn0027619 Adam CG12131 GH12681
	FBgn0000054 Adf1 CG15845 LD09689
cardiac mesoderm primordium;	
	FBgn0040297 NHE2 CG9256 RE21674
embryonic ganglion mother cell;	
	FBgn0040297 NHE2 CG9256 RE21674
embryonic salivary gland body;	
	FBgn0014868 Ost48 CG9022 LD21941
	FBgn0000054 Adf1 CG15845 LD09689
	FBgn0000659 fkh CG10002 RE03865
	FBgn0051810 CG31810 CG31810 RE06583
	FBgn0000054 Adf1 CG15845 LD09689
lamellocyte specific anlage;	
	FBgn0000257 car CG12230 LD22396
	FBgn0000054 Adf1 CG15845 LD09689
	FBgn0025631 EG:22E5.11 CG4322 RE06985
	FBgn0033562 CG6751 CG6751 RE21021
	FBgn0040297 NHE2 CG9256 RE21674
	FBgn0051431 CG31431 CG31431 RE47547
	FBgn0000054 Adf1 CG15845 LD09689
crystal cell specific anlage;	
	FBgn0027621 Pfrx CG3400 GH17337
	FBgn0040260 Ugt36Bc CG17932 RE03785
	FBgn0025631 EG:22E5.11 CG4322 RE06985
	FBgn0051431 CG31431 CG31431 RE47547
embryonic large intestine;	
	FBgn0011638 La CG10922 RE69138
	FBgn0000659 fkh CG10002 RE03865
