# Supplementary material for: Prediction of Gene Expression in Embryonic Structures of Drosophila melanogaster
Source: PLoS Comput Biol. 2007 Jul 20;3(7):e144. doi: 10.1371/journal.pcbi.0030144 (PMC1924873; doi:10.1371/journal.pcbi.0030144)
Supplement: Supplementary file 18 [file pcbi.0030144.st018.pdf]

Table 18: **Diversity of anatomical annotations of genes comprising functional unit pep2ebrain**: embryonic central brain && protocerebrum primordium && procephalic ectoderm primordium

Anatomical Structures	Genes
labial sensory complex primordium;	
	FBgn0004595 pros CG17228 GH11848
embryonic optic lobe primordium;	
	FBgn0002932 neur CG11988 LD45505
	FBgn0033992 CG11798 CG11798 LD23883
	FBgn0036786 skl CG13701 RE14076
	FBgn0035186 CG13912 CG13912 RE20371
	FBgn0010453 Wnt4 CG4698 RE26454
fat body/gonad primordium;	
	FBgn0037811 CG12592 CG12592 AT09724
dorsal prothoracic pharyngeal muscle;	
	FBgn0031971 CG7224 CG7224 GH18422
dorsal pharyngeal muscle primordium;	
	FBgn0033992 CG11798 CG11798 LD23883
ventral midline;	
	FBgn0004868 Gdi CG4422 LD46767
	FBgn0035542 CG11347 CG11347 GH28550
	FBgn0040271 Sulf1 CG6725 SD04414
	FBgn0004584 Rrp1 CG3178 LP05366
	FBgn0000577 en CG9015 LD16125
	FBgn0000659 fkh CG10002 RE03865
	FBgn0015773 NetA CG18657 RE11206
	FBgn0000577 en CG9015 LD16125
small intestine specific anlage;	
	FBgn0000659 fkh CG10002 RE03865
embryonic midgut;	
	FBgn0040271 Sulf1 CG6725 LD03060
	FBgn0004868 Gdi CG4422 LD46767
	FBgn0040271 Sulf1 CG6725 SD04414
	FBgn0037811 CG12592 CG12592 AT09724
	FBgn0000611 exd CG8933 LD03509
	FBgn0038118 NULL CG7855 RE03551
	FBgn0000659 fkh CG10002 RE03865
	FBgn0010453 Wnt4 CG4698 RE26454
procephalic ectoderm primordium;	
	FBgn0040271 Sulf1 CG6725 LD03060
	FBgn0024332 Mcm3 CG4206 LD07144
	FBgn0013469 NULL CG12296 RE58581
	FBgn0036494 Toll-6 CG7250 LD08841
	FBgn0004868 Gdi CG4422 LD46767
	FBgn0002932 neur CG11988 LD45505
	FBgn0035542 CG11347 CG11347 GH28550
	FBgn0040271 Sulf1 CG6725 SD04414

continued on next page...

Table 18 continued from previous page.	
Anatomical Structures	Genes
	FBgn0004595 pros CG17228 GH11848<br>FBgn0040230 dbo CG6224 RE13447<br>FBgn0002561 l(1)sc CG3839 RE59335<br>FBgn0037947 CG6930 CG6930 RE72719<br>FBgn0037811 CG12592 CG12592 AT09724<br>FBgn0005677 dac CG4952 RE64054<br>FBgn0003300 run CG1849 GH02614<br>FBgn0051670 CG31670 CG31670 GH14092<br>FBgn0004584 Rrp1 CG3178 LP05366<br>FBgn0015903 apt CG5393 LD45581<br>FBgn0033992 CG11798 CG11798 LD23883<br>FBgn0039211 CG13620 CG13620 SD03914<br>FBgn0000611 exd CG8933 LD03509<br>FBgn0030507 CG11164 CG11164 RE50056<br>FBgn0034878 CG3941 CG3941 LD15650<br>FBgn0000577 en CG9015 LD16125<br>FBgn0003346 Sd CG9999 LD16356<br>FBgn0038118 NULL CG7855 RE03551<br>FBgn0000659 fkh CG10002 RE03865<br>FBgn0017453 Zn72D CG5215 GH12756<br>FBgn0015773 NetA CG18657 RE11206<br>FBgn0004567 slp2 CG2939 RE11345<br>FBgn0036786 skl CG13701 RE14076<br>FBgn0038150 yellow-e3 CG17045 RE14803<br>FBgn0035186 CG13912 CG13912 RE20371<br>FBgn0010453 Wnt4 CG4698 RE26454<br>FBgn0013725 phyl CG10108 RH04401<br>FBgn0029512 Aos1 CG12276 LD33652<br>FBgn0031971 CG7224 CG7224 GH18422<br>FBgn0051670 CG31670 CG31670 GH14092<br>FBgn0034878 CG3941 CG3941 LD15650<br>FBgn0000577 en CG9015 LD16125
embryonic/larval circulatory system;	FBgn0015903 apt CG5393 LD45581
Malpighian tubule primordium;	FBgn0000659 fkh CG10002 RE03865
ventral neuroderm anlage;	FBgn0036494 Toll-6 CG7250 LD08841<br>FBgn0002932 neur CG11988 LD45505<br>FBgn0040271 Sulf1 CG6725 SD04414<br>FBgn0040230 dbo CG6224 RE13447<br>FBgn0002561 l(1)sc CG3839 RE59335<br>FBgn0003300 run CG1849 GH02614<br>FBgn0015903 apt CG5393 LD45581<br>FBgn0033992 CG11798 CG11798 LD23883<br>FBgn0000611 exd CG8933 LD03509

continued on next page...

Table 18 continued from previous page.

Anatomical Structures	Genes
	FBgn0030507 CG11164 CG11164 RE50056
	FBgn0000577 en CG9015 LD16125
	FBgn0003346 Sd CG9999 LD16356
	FBgn0038118 NULL CG7855 RE03551
	FBgn0017453 Zn72D CG5215 GH12756
	FBgn0015773 NetA CG18657 RE11206
	FBgn0010453 Wnt4 CG4698 RE26454
	FBgn0029512 Aos1 CG12276 LD33652
	FBgn0000577 en CG9015 LD16125
need new term(larval eye primordium);	
	FBgn0004595 pros CG17228 GH11848
	FBgn0033992 CG11798 CG11798 LD23883
labral sensory complex primordium;	
	FBgn0004595 pros CG17228 GH11848
embryonic inner optic lobe;	
	FBgn0036786 skl CG13701 RE14076
	FBgn0010453 Wnt4 CG4698 RE26454
procephalic ectoderm anlage;	
	FBgn0036494 Toll-6 CG7250 LD08841
	FBgn0002932 neur CG11988 LD45505
	FBgn0035542 CG11347 CG11347 GH28550
	FBgn0004595 pros CG17228 GH11848
	FBgn0040230 dbo CG6224 RE13447
	FBgn0002561 l(1)sc CG3839 RE59335
	FBgn0003300 run CG1849 GH02614
	FBgn0051670 CG31670 CG31670 GH14092
	FBgn0015903 apt CG5393 LD45581
	FBgn0033992 CG11798 CG11798 LD23883
	FBgn0039211 CG13620 CG13620 SD03914
	FBgn0000611 exd CG8933 LD03509
	FBgn0030507 CG11164 CG11164 RE50056
	FBgn0000577 en CG9015 LD16125
	FBgn0003346 Sd CG9999 LD16356
	FBgn0038118 NULL CG7855 RE03551
	FBgn0038150 yellow-e3 CG17045 RE14803
	FBgn0035186 CG13912 CG13912 RE20371
	FBgn0010453 Wnt4 CG4698 RE26454
	FBgn0029512 Aos1 CG12276 LD33652
	FBgn0051670 CG31670 CG31670 GH14092
	FBgn0000577 en CG9015 LD16125
ring gland;	
	FBgn0004584 Rrp1 CG3178 LP05366
	FBgn0015903 apt CG5393 LD45581
	FBgn0034878 CG3941 CG3941 LD15650
	FBgn0031971 CG7224 CG7224 GH18422
dorsal ectoderm anlage;	

continued on next page...

Table 18 continued from previous page.	
Anatomical Structures	Genes
	FBgn0035542 CG11347 CG11347 GH28550<br>FBgn0003300 run CG1849 GH02614<br>FBgn0033992 CG11798 CG11798 LD23883<br>FBgn0000611 exd CG8933 LD03509<br>FBgn0000577 en CG9015 LD16125<br>FBgn0003346 Sd CG9999 LD16356<br>FBgn0038150 yellow-e3 CG17045 RE14803<br>FBgn0000577 en CG9015 LD16125
mesectoderm primordium;	FBgn0002932 neur CG11988 LD45505<br>FBgn0033992 CG11798 CG11798 LD23883<br>FBgn0000577 en CG9015 LD16125<br>FBgn0003346 Sd CG9999 LD16356<br>FBgn0015773 NetA CG18657 RE11206<br>FBgn0004567 slp2 CG2939 RE11345<br>FBgn0000577 en CG9015 LD16125
embryonic gastric caecum;	FBgn0010453 Wnt4 CG4698 RE26454
pericardial cell specific anlage;	FBgn0015903 apt CG5393 LD45581
circular visceral muscle fibers;	FBgn0015773 NetA CG18657 RE11206
embryonic/larval muscle system;	FBgn0000611 exd CG8933 LD03509<br>FBgn0015773 NetA CG18657 RE11206
anterior endoderm primordium;	FBgn0037811 CG12592 CG12592 AT09724<br>FBgn0015903 apt CG5393 LD45581<br>FBgn0000611 exd CG8933 LD03509<br>FBgn0003346 Sd CG9999 LD16356<br>FBgn0000659 fkh CG10002 RE03865
embryonic rectum;	FBgn0000659 fkh CG10002 RE03865<br>FBgn0010453 Wnt4 CG4698 RE26454
posterior midgut primordium;	FBgn0002561 l(1)sc CG3839 RE59335<br>FBgn0037811 CG12592 CG12592 AT09724<br>FBgn0000659 fkh CG10002 RE03865<br>FBgn0017453 Zn72D CG5215 GH12756
ventral sensory complex primordium;	FBgn0004868 Gdi CG4422 LD46767<br>FBgn0002932 neur CG11988 LD45505<br>FBgn0040271 Sulf1 CG6725 SD04414<br>FBgn0004595 pros CG17228 GH11848<br>FBgn0037947 CG6930 CG6930 RE72719<br>FBgn0003300 run CG1849 GH02614
	continued on next page...

Table 18 continued from previous page.	
Anatomical Structures	Genes
	FBgn0033992 CG11798 CG11798 LD23883
posterior endoderm primordium;	
	FBgn0002561 l(1)sc CG3839 RE59335
	FBgn0037811 CG12592 CG12592 AT09724
	FBgn0015903 apt CG5393 LD45581
	FBgn0000611 exd CG8933 LD03509
	FBgn0003346 Sd CG9999 LD16356
	FBgn0000659 fkh CG10002 RE03865
embryonic corpus cardiacum;	
	FBgn0031971 CG7224 CG7224 GH18422
embryonic central brain glia;	
	FBgn0002932 neur CG11988 LD45505
	FBgn0037811 CG12592 CG12592 AT09724
	FBgn0004584 Rrp1 CG3178 LP05366
	FBgn0033992 CG11798 CG11798 LD23883
	FBgn0000611 exd CG8933 LD03509
	FBgn0017453 Zn72D CG5215 GH12756
	FBgn0029512 Aos1 CG12276 LD33652
ventral midline neuroblast;	
	FBgn0004595 pros CG17228 GH11848
midline primordium;	
	FBgn0040271 Sulf1 CG6725 SD04414
	FBgn0002561 l(1)sc CG3839 RE59335
	FBgn0000577 en CG9015 LD16125
	FBgn0000659 fkh CG10002 RE03865
	FBgn0015773 NetA CG18657 RE11206
	FBgn0000577 en CG9015 LD16125
hindgut proper primordium;	
	FBgn0004868 Gdi CG4422 LD46767
	FBgn0040230 dbo CG6224 RE13447
	FBgn0037811 CG12592 CG12592 AT09724
	FBgn0051670 CG31670 CG31670 GH14092
	FBgn0000577 en CG9015 LD16125
	FBgn0000659 fkh CG10002 RE03865
	FBgn0017453 Zn72D CG5215 GH12756
	FBgn0036786 skl CG13701 RE14076
	FBgn0038150 yellow-e3 CG17045 RE14803
	FBgn0010453 Wnt4 CG4698 RE26454
	FBgn0031971 CG7224 CG7224 GH18422
	FBgn0051670 CG31670 CG31670 GH14092
	FBgn0000577 en CG9015 LD16125
labral sensory complex;	
	FBgn0004868 Gdi CG4422 LD46767
	FBgn0004595 pros CG17228 GH11848
	FBgn0005677 dac CG4952 RE64054
proventriculus primordium;	
	continued on next page...

Table 18 continued from previous page.

Anatomical Structures	Genes
	FBgn0003346 Sd CG9999 LD16356<br>FBgn0000659 fkh CG10002 RE03865
pole cell;	FBgn0039211 CG13620 CG13620 SD03914
embryonic central brain surface glia;	FBgn0013469 NULL CG12296 RE58581<br>FBgn0002932 neur CG11988 LD45505<br>FBgn0004584 Rrp1 CG3178 LP05366<br>FBgn0030507 CG11164 CG11164 RE50056
antennal primordium2;	FBgn0003346 Sd CG9999 LD16356
embryonic Bolwig's organ;	FBgn0004868 Gdi CG4422 LD46767<br>FBgn0004595 pros CG17228 GH11848
large intestine specific anlage;	FBgn0040230 dbo CG6224 RE13447<br>FBgn0000659 fkh CG10002 RE03865
longitudinal visceral mesoderm primordium;	FBgn0033992 CG11798 CG11798 LD23883<br>FBgn0010453 Wnt4 CG4698 RE26454
external foregut primordium;	FBgn0037811 CG12592 CG12592 AT09724<br>FBgn0015903 apt CG5393 LD45581<br>FBgn0000577 en CG9015 LD16125<br>FBgn0003346 Sd CG9999 LD16356<br>FBgn0000659 fkh CG10002 RE03865<br>FBgn0036786 skl CG13701 RE14076<br>FBgn0038150 yellow-e3 CG17045 RE14803<br>FBgn0010453 Wnt4 CG4698 RE26454<br>FBgn0000577 en CG9015 LD16125
lateral cord glia;	FBgn0002932 neur CG11988 LD45505<br>FBgn0037811 CG12592 CG12592 AT09724<br>FBgn0004584 Rrp1 CG3178 LP05366<br>FBgn0033992 CG11798 CG11798 LD23883<br>FBgn0000611 exd CG8933 LD03509<br>FBgn0038118 NULL CG7855 RE03551<br>FBgn0017453 Zn72D CG5215 GH12756<br>FBgn0029512 Aos1 CG12276 LD33652
embryonic foregut sensory structure;	FBgn0004595 pros CG17228 GH11848
head epidermis primordium;	FBgn0015903 apt CG5393 LD45581<br>FBgn0003346 Sd CG9999 LD16356
embryonic inner optic lobe primordium;	FBgn0010453 Wnt4 CG4698 RE26454

continued on next page...

Table 18 continued from previous page.	
Anatomical Structures	Genes
salivary gland body primordium;	FBgn0000659 fkh CG10002 RE03865
sensory nervous system primordium;	FBgn0040271 Sulf1 CG6725 LD03060<br>FBgn0013469 NULL CG12296 RE58581<br>FBgn0004868 Gdi CG4422 LD46767<br>FBgn0002932 neur CG11988 LD45505<br>FBgn0004595 pros CG17228 GH11848<br>FBgn0037947 CG6930 CG6930 RE72719<br>FBgn0003300 run CG1849 GH02614<br>FBgn0017453 Zn72D CG5215 GH12756
embryonic hindgut;	FBgn0040271 Sulf1 CG6725 LD03060<br>FBgn0004868 Gdi CG4422 LD46767<br>FBgn0040230 dbo CG6224 RE13447<br>FBgn0037811 CG12592 CG12592 AT09724<br>FBgn0005677 dac CG4952 RE64054<br>FBgn0000659 fkh CG10002 RE03865<br>FBgn0017453 Zn72D CG5215 GH12756<br>FBgn0038150 yellow-e3 CG17045 RE14803<br>FBgn0010453 Wnt4 CG4698 RE26454
embryonic anal pad;	FBgn0000659 fkh CG10002 RE03865
ventral nerve cord;	FBgn0040271 Sulf1 CG6725 LD03060<br>FBgn0024332 Mcm3 CG4206 LD07144<br>FBgn0013469 NULL CG12296 RE58581<br>FBgn0004868 Gdi CG4422 LD46767<br>FBgn0002932 neur CG11988 LD45505<br>FBgn0035542 CG11347 CG11347 GH28550<br>FBgn0040271 Sulf1 CG6725 SD04414<br>FBgn0004595 pros CG17228 GH11848<br>FBgn0040230 dbo CG6224 RE13447<br>FBgn0037947 CG6930 CG6930 RE72719<br>FBgn0037811 CG12592 CG12592 AT09724<br>FBgn0005677 dac CG4952 RE64054<br>FBgn0003300 run CG1849 GH02614<br>FBgn0004584 Rrp1 CG3178 LP05366<br>FBgn0015903 apt CG5393 LD45581<br>FBgn0033992 CG11798 CG11798 LD23883<br>FBgn0000611 exd CG8933 LD03509<br>FBgn0030507 CG11164 CG11164 RE50056<br>FBgn0034878 CG3941 CG3941 LD15650<br>FBgn0000577 en CG9015 LD16125<br>FBgn0003346 Sd CG9999 LD16356<br>FBgn0017453 Zn72D CG5215 GH12756
	continued on next page...

Table 18 continued from previous page.

Anatomical Structures	Genes
	FBgn0004567 slp2 CG2939 RE11345<br>FBgn0036786 skl CG13701 RE14076<br>FBgn0035186 CG13912 CG13912 RE20371<br>FBgn0010453 Wnt4 CG4698 RE26454<br>FBgn0029512 Aos1 CG12276 LD33652<br>FBgn0034878 CG3941 CG3941 LD15650<br>FBgn0000577 en CG9015 LD16125
visual primordium;	FBgn0002932 neur CG11988 LD45505<br>FBgn0004595 pros CG17228 GH11848<br>FBgn0002561 l(1)sc CG3839 RE59335<br>FBgn0015903 apt CG5393 LD45581<br>FBgn0033992 CG11798 CG11798 LD23883<br>FBgn0000611 exd CG8933 LD03509<br>FBgn0003346 Sd CG9999 LD16356<br>FBgn0036786 skl CG13701 RE14076<br>FBgn0035186 CG13912 CG13912 RE20371<br>FBgn0010453 Wnt4 CG4698 RE26454<br>FBgn0013725 phyl CG10108 RH04401
foregut primordium;	FBgn0039211 CG13620 CG13620 SD03914<br>FBgn0000659 fkh CG10002 RE03865<br>FBgn0036786 skl CG13701 RE14076<br>FBgn0038150 yellow-e3 CG17045 RE14803<br>FBgn0010453 Wnt4 CG4698 RE26454
crystal cell;	FBgn0031971 CG7224 CG7224 GH18422
embryonic leading edge cell specific anlage;	FBgn0010453 Wnt4 CG4698 RE26454
embryonic ventral epidermis;	FBgn0004868 Gdi CG4422 LD46767<br>FBgn0040230 dbo CG6224 RE13447<br>FBgn0037947 CG6930 CG6930 RE72719<br>FBgn0015773 NetA CG18657 RE11206<br>FBgn0004567 slp2 CG2939 RE11345<br>FBgn0038150 yellow-e3 CG17045 RE14803<br>FBgn0035186 CG13912 CG13912 RE20371
lateral cord neuron;	FBgn0024332 Mcm3 CG4206 LD07144<br>FBgn0013469 NULL CG12296 RE58581<br>FBgn0036494 Toll-6 CG7250 LD08841<br>FBgn0004868 Gdi CG4422 LD46767<br>FBgn0002932 neur CG11988 LD45505<br>FBgn0035542 CG11347 CG11347 GH28550<br>FBgn0040271 Sulf1 CG6725 SD04414<br>FBgn0040230 dbo CG6224 RE13447

continued on next page...

Table 18 continued from previous page.	
Anatomical Structures	Genes
	FBgn0037947 CG6930 CG6930 RE72719<br>FBgn0037811 CG12592 CG12592 AT09724<br>FBgn0005677 dac CG4952 RE64054<br>FBgn0004584 Rrp1 CG3178 LP05366<br>FBgn0033992 CG11798 CG11798 LD23883<br>FBgn0000611 exd CG8933 LD03509<br>FBgn0034878 CG3941 CG3941 LD15650<br>FBgn0000577 en CG9015 LD16125<br>FBgn0038118 NULL CG7855 RE03551<br>FBgn0017453 Zn72D CG5215 GH12756<br>FBgn0015773 NetA CG18657 RE11206<br>FBgn0010453 Wnt4 CG4698 RE26454<br>FBgn0013725 phyl CG10108 RH04401<br>FBgn0029512 Aos1 CG12276 LD33652<br>FBgn0034878 CG3941 CG3941 LD15650<br>FBgn0000577 en CG9015 LD16125
mesectoderm anlage;	FBgn0002932 neur CG11988 LD45505<br>FBgn0033992 CG11798 CG11798 LD23883<br>FBgn0000611 exd CG8933 LD03509<br>FBgn0000577 en CG9015 LD16125<br>FBgn0003346 Sd CG9999 LD16356<br>FBgn0000577 en CG9015 LD16125
visceral muscle primordium;	FBgn0035542 CG11347 CG11347 GH28550<br>FBgn0033992 CG11798 CG11798 LD23883<br>FBgn0015773 NetA CG18657 RE11206<br>FBgn0031971 CG7224 CG7224 GH18422
amnioserosa;	FBgn0035542 CG11347 CG11347 GH28550<br>FBgn0000659 fkh CG10002 RE03865
embryonic optic lobe;	FBgn0033992 CG11798 CG11798 LD23883<br>FBgn0036786 skl CG13701 RE14076<br>FBgn0035186 CG13912 CG13912 RE20371<br>FBgn0010453 Wnt4 CG4698 RE26454
embryonic/larval pericardial cell;	FBgn0015903 apt CG5393 LD45581
embryonic dorsal epidermis;	FBgn0004868 Gdi CG4422 LD46767<br>FBgn0035542 CG11347 CG11347 GH28550<br>FBgn0037947 CG6930 CG6930 RE72719<br>FBgn0000577 en CG9015 LD16125<br>FBgn0015773 NetA CG18657 RE11206<br>FBgn0038150 yellow-e3 CG17045 RE14803<br>FBgn0035186 CG13912 CG13912 RE20371

continued on next page...

Table 18 continued from previous page.

Anatomical Structures	Genes
	FBgn0000577 en CG9015 LD16125
gnathal primordium;	FBgn0015903 apt CG5393 LD45581<br>FBgn0000577 en CG9015 LD16125<br>FBgn0036786 skl CG13701 RE14076<br>FBgn0010453 Wnt4 CG4698 RE26454<br>FBgn0000577 en CG9015 LD16125
posterior spiracle specific anlage;	FBgn0039211 CG13620 CG13620 SD03914
head mesoderm P2 primordium;	FBgn0033992 CG11798 CG11798 LD23883<br>FBgn0000611 exd CG8933 LD03509<br>FBgn0003346 Sd CG9999 LD16356<br>FBgn0015773 NetA CG18657 RE11206
anterior midgut primordium;	FBgn0037811 CG12592 CG12592 AT09724<br>FBgn0000659 fkh CG10002 RE03865<br>FBgn0017453 Zn72D CG5215 GH12756
clypeo-labral primordium;	FBgn0040230 dbo CG6224 RE13447<br>FBgn0005677 dac CG4952 RE64054<br>FBgn0015903 apt CG5393 LD45581<br>FBgn0000577 en CG9015 LD16125<br>FBgn0004567 slp2 CG2939 RE11345<br>FBgn0036786 skl CG13701 RE14076<br>FBgn0038150 yellow-e3 CG17045 RE14803<br>FBgn0010453 Wnt4 CG4698 RE26454<br>FBgn0000577 en CG9015 LD16125
cardiac mesoderm primordium;	FBgn0033992 CG11798 CG11798 LD23883
maxillary sensory complex primordium;	FBgn0004595 pros CG17228 GH11848
embryonic ganglion mother cell;	FBgn0024332 Mcm3 CG4206 LD07144<br>FBgn0013469 NULL CG12296 RE58581<br>FBgn0002932 neur CG11988 LD45505<br>FBgn0004595 pros CG17228 GH11848<br>FBgn0030507 CG11164 CG11164 RE50056<br>FBgn0034878 CG3941 CG3941 LD15650<br>FBgn0035186 CG13912 CG13912 RE20371<br>FBgn0010453 Wnt4 CG4698 RE26454<br>FBgn0034878 CG3941 CG3941 LD15650
embryonic salivary gland body;	FBgn0000659 fkh CG10002 RE03865
embryonic large intestine;	FBgn0040230 dbo CG6224 RE13447

continued on next page...

Table 18 continued from previous page.

Anatomical Structures	Genes
	FBgn0005677 dac CG4952 RE64054<br>FBgn0000577 en CG9015 LD16125<br>FBgn0000659 fkh CG10002 RE03865<br>FBgn0010453 Wnt4 CG4698 RE26454<br>FBgn0000577 en CG9015 LD16125
germ cell;	FBgn0037811 CG12592 CG12592 AT09724
embryonic esophagus;	FBgn0004868 Gdi CG4422 LD46767<br>FBgn0000659 fkh CG10002 RE03865<br>FBgn0038150 yellow-e3 CG17045 RE14803<br>FBgn0010453 Wnt4 CG4698 RE26454
embryonic epipharynx;	FBgn0004868 Gdi CG4422 LD46767<br>FBgn0015903 apt CG5393 LD45581<br>FBgn0039211 CG13620 CG13620 SD03914<br>FBgn0000577 en CG9015 LD16125<br>FBgn0004567 slp2 CG2939 RE11345<br>FBgn0038150 yellow-e3 CG17045 RE14803<br>FBgn0010453 Wnt4 CG4698 RE26454<br>FBgn0000577 en CG9015 LD16125
gonad;	FBgn0037811 CG12592 CG12592 AT09724<br>FBgn0004584 Rrp1 CG3178 LP05366<br>FBgn0034878 CG3941 CG3941 LD15650<br>FBgn0017453 Zn72D CG5215 GH12756<br>FBgn0029512 Aos1 CG12276 LD33652<br>FBgn0034878 CG3941 CG3941 LD15650
embryonic maxillary sensory complex;	FBgn0004868 Gdi CG4422 LD46767<br>FBgn0004595 pros CG17228 GH11848
head mesoderm anlage;	FBgn0024332 Mcm3 CG4206 LD07144<br>FBgn0002932 neur CG11988 LD45505<br>FBgn0033992 CG11798 CG11798 LD23883<br>FBgn0000611 exd CG8933 LD03509<br>FBgn0003346 Sd CG9999 LD16356<br>FBgn0015773 NetA CG18657 RE11206<br>FBgn0035186 CG13912 CG13912 RE20371
embryonic leading edge cell;	FBgn0004595 pros CG17228 GH11848
embryonic proventriculus;	FBgn0004868 Gdi CG4422 LD46767<br>FBgn0000659 fkh CG10002 RE03865<br>FBgn0015773 NetA CG18657 RE11206<br>FBgn0010453 Wnt4 CG4698 RE26454

continued on next page...

Table 18 continued from previous page.	
Anatomical Structures	Genes
dorsal ectoderm primordium;	FBgn0040271 Sulf1 CG6725 LD03060<br>FBgn0036494 Toll-6 CG7250 LD08841<br>FBgn0035542 CG11347 CG11347 GH28550<br>FBgn0040271 Sulf1 CG6725 SD04414<br>FBgn0037811 CG12592 CG12592 AT09724<br>FBgn0003300 run CG1849 GH02614<br>FBgn0015903 apt CG5393 LD45581<br>FBgn0033992 CG11798 CG11798 LD23883<br>FBgn0000611 exd CG8933 LD03509<br>FBgn0003346 Sd CG9999 LD16356<br>FBgn0038150 yellow-e3 CG17045 RE14803<br>FBgn0013725 phyl CG10108 RH04401
dorsal epidermis primordium;	FBgn0040271 Sulf1 CG6725 LD03060<br>FBgn0013469 NULL CG12296 RE58581<br>FBgn0036494 Toll-6 CG7250 LD08841<br>FBgn0035542 CG11347 CG11347 GH28550<br>FBgn0040271 Sulf1 CG6725 SD04414<br>FBgn0037947 CG6930 CG6930 RE72719<br>FBgn0037811 CG12592 CG12592 AT09724<br>FBgn0033992 CG11798 CG11798 LD23883<br>FBgn0000611 exd CG8933 LD03509<br>FBgn0000577 en CG9015 LD16125<br>FBgn0038150 yellow-e3 CG17045 RE14803<br>FBgn0010453 Wnt4 CG4698 RE26454<br>FBgn0000577 en CG9015 LD16125
embryonic stomatogastric nervous system;	FBgn0002561 l(1)sc CG3839 RE59335<br>FBgn0000659 fkh CG10002 RE03865<br>FBgn0013725 phyl CG10108 RH04401
embryonic/larval somatic muscle;	FBgn0040271 Sulf1 CG6725 SD04414<br>FBgn0000611 exd CG8933 LD03509
embryonic central brain mushroom body;	FBgn0037811 CG12592 CG12592 AT09724<br>FBgn0004584 Rrp1 CG3178 LP05366
embryonic salivary gland;	FBgn0000659 fkh CG10002 RE03865
ventral sensory complex specific anlage;	FBgn0002932 neur CG11988 LD45505<br>FBgn0004595 pros CG17228 GH11848<br>FBgn0002561 l(1)sc CG3839 RE59335
embryonic hypopharynx;	FBgn0004868 Gdi CG4422 LD46767<br>FBgn0015903 apt CG5393 LD45581
	continued on next page...

Table 18 continued from previous page.	
Anatomical Structures	Genes
	FBgn0039211 CG13620 CG13620 SD03914
embryonic/larval dorsal vessel;	FBgn0015903 apt CG5393 LD45581
embryonic central brain pars intercerebralis;	FBgn0040271 Sulf1 CG6725 LD03060<br>FBgn0040271 Sulf1 CG6725 SD04414<br>FBgn0038118 NULL CG7855 RE03551<br>FBgn0031971 CG7224 CG7224 GH18422
embryonic central brain;	FBgn0040271 Sulf1 CG6725 LD03060<br>FBgn0024332 Mcm3 CG4206 LD07144<br>FBgn0013469 NULL CG12296 RE58581<br>FBgn0036494 Toll-6 CG7250 LD08841<br>FBgn0004868 Gdi CG4422 LD46767<br>FBgn0002932 neur CG11988 LD45505<br>FBgn0035542 CG11347 CG11347 GH28550<br>FBgn0040271 Sulf1 CG6725 SD04414<br>FBgn0004595 pros CG17228 GH11848<br>FBgn0040230 dbo CG6224 RE13447<br>FBgn0002561 l(1)sc CG3839 RE59335<br>FBgn0037947 CG6930 CG6930 RE72719<br>FBgn0037811 CG12592 CG12592 AT09724<br>FBgn0005677 dac CG4952 RE64054<br>FBgn0003300 run CG1849 GH02614<br>FBgn0051670 CG31670 CG31670 GH14092<br>FBgn0004584 Rrp1 CG3178 LP05366<br>FBgn0015903 apt CG5393 LD45581<br>FBgn0033992 CG11798 CG11798 LD23883<br>FBgn0039211 CG13620 CG13620 SD03914<br>FBgn0000611 exd CG8933 LD03509<br>FBgn0030507 CG11164 CG11164 RE50056<br>FBgn0034878 CG3941 CG3941 LD15650<br>FBgn0000577 en CG9015 LD16125<br>FBgn0003346 Sd CG9999 LD16356<br>FBgn0038118 NULL CG7855 RE03551<br>FBgn0000659 fkh CG10002 RE03865<br>FBgn0017453 Zn72D CG5215 GH12756<br>FBgn0015773 NetA CG18657 RE11206<br>FBgn0004567 slp2 CG2939 RE11345<br>FBgn0036786 skl CG13701 RE14076<br>FBgn0038150 yellow-e3 CG17045 RE14803<br>FBgn0035186 CG13912 CG13912 RE20371<br>FBgn0010453 Wnt4 CG4698 RE26454<br>FBgn0013725 phyl CG10108 RH04401<br>FBgn0029512 Aos1 CG12276 LD33652<br>FBgn0031971 CG7224 CG7224 GH18422

continued on next page...

Table 18 continued from previous page.

Anatomical Structures	Genes
	FBgn0051670 CG31670 CG31670 GH14092<br>FBgn0034878 CG3941 CG3941 LD15650<br>FBgn0000577 en CG9015 LD16125
salivary gland body specific anlage;	FBgn0000659 fkh CG10002 RE03865
ventral ectoderm primordium;	FBgn0036494 Toll-6 CG7250 LD08841<br>FBgn0035542 CG11347 CG11347 GH28550<br>FBgn0040230 dbo CG6224 RE13447<br>FBgn0037947 CG6930 CG6930 RE72719<br>FBgn0037811 CG12592 CG12592 AT09724<br>FBgn0003300 run CG1849 GH02614<br>FBgn0015903 apt CG5393 LD45581<br>FBgn0033992 CG11798 CG11798 LD23883<br>FBgn0000611 exd CG8933 LD03509<br>FBgn0030507 CG11164 CG11164 RE50056<br>FBgn0000577 en CG9015 LD16125<br>FBgn0003346 Sd CG9999 LD16356<br>FBgn0004567 slp2 CG2939 RE11345<br>FBgn0035186 CG13912 CG13912 RE20371<br>FBgn0010453 Wnt4 CG4698 RE26454<br>FBgn0013725 phyl CG10108 RH04401<br>FBgn0000577 en CG9015 LD16125
embryonic central brain neuron;	FBgn0024332 Mcm3 CG4206 LD07144<br>FBgn0002932 neur CG11988 LD45505<br>FBgn0035542 CG11347 CG11347 GH28550<br>FBgn0040230 dbo CG6224 RE13447<br>FBgn0037947 CG6930 CG6930 RE72719<br>FBgn0037811 CG12592 CG12592 AT09724<br>FBgn0051670 CG31670 CG31670 GH14092<br>FBgn0004584 Rrp1 CG3178 LP05366<br>FBgn0033992 CG11798 CG11798 LD23883<br>FBgn0039211 CG13620 CG13620 SD03914<br>FBgn0000611 exd CG8933 LD03509<br>FBgn0000577 en CG9015 LD16125<br>FBgn0038118 NULL CG7855 RE03551<br>FBgn0017453 Zn72D CG5215 GH12756<br>FBgn0015773 NetA CG18657 RE11206<br>FBgn0036786 skl CG13701 RE14076<br>FBgn0038150 yellow-e3 CG17045 RE14803<br>FBgn0010453 Wnt4 CG4698 RE26454<br>FBgn0013725 phyl CG10108 RH04401<br>FBgn0029512 Aos1 CG12276 LD33652<br>FBgn0051670 CG31670 CG31670 GH14092<br>FBgn0000577 en CG9015 LD16125

continued on next page...

Table 18 continued from previous page.	
Anatomical Structures	Genes
embryonic labial sensory complex;	
	FBgn0004868 Gdi CG4422 LD46767
	FBgn0004595 pros CG17228 GH11848
visual anlage;	
	FBgn0015903 apt CG5393 LD45581
	FBgn0033992 CG11798 CG11798 LD23883
	FBgn0000611 exd CG8933 LD03509
	FBgn0003346 Sd CG9999 LD16356
	FBgn0035186 CG13912 CG13912 RE20371
Malpighian tubule main body primordium;	
	FBgn0000659 fkh CG10002 RE03865
inclusive hindgut primordium;	
	FBgn0004868 Gdi CG4422 LD46767
	FBgn0040230 dbo CG6224 RE13447
	FBgn0037811 CG12592 CG12592 AT09724
	FBgn0003300 run CG1849 GH02614
	FBgn0051670 CG31670 CG31670 GH14092
	FBgn0015903 apt CG5393 LD45581
	FBgn0000611 exd CG8933 LD03509
	FBgn0000577 en CG9015 LD16125
	FBgn0003346 Sd CG9999 LD16356
	FBgn0000659 fkh CG10002 RE03865
	FBgn0036786 skl CG13701 RE14076
	FBgn0031971 CG7224 CG7224 GH18422
	FBgn0051670 CG31670 CG31670 GH14092
	FBgn0000577 en CG9015 LD16125
foregut anlage;	
	FBgn0002932 neur CG11988 LD45505
	FBgn0015903 apt CG5393 LD45581
	FBgn0000611 exd CG8933 LD03509
	FBgn0003346 Sd CG9999 LD16356
	FBgn0000659 fkh CG10002 RE03865
	FBgn0035186 CG13912 CG13912 RE20371
	FBgn0010453 Wnt4 CG4698 RE26454
somatic muscle primordium;	
	FBgn0040271 Sulf1 CG6725 SD04414
	FBgn0002561 l(1)sc CG3839 RE59335
	FBgn0033992 CG11798 CG11798 LD23883
	FBgn0000577 en CG9015 LD16125
	FBgn0000577 en CG9015 LD16125
lateral cord surface glia;	
	FBgn0002932 neur CG11988 LD45505
	FBgn0004584 Rrp1 CG3178 LP05366
	FBgn0038118 NULL CG7855 RE03551
need new term(sensory system head);	
	FBgn0035542 CG11347 CG11347 GH28550
	continued on next page...

Table 18 continued from previous page.	
Anatomical Structures	Genes
	FBgn0004595 pros CG17228 GH11848
	FBgn0033992 CG11798 CG11798 LD23883
	FBgn0017453 Zn72D CG5215 GH12756
stomatogastric nervous system primordium;	
	FBgn0002561 l(1)sc CG3839 RE59335
	FBgn0000659 fkh CG10002 RE03865
	FBgn0013725 phyl CG10108 RH04401
embryonic/larval posterior spiracle;	
	FBgn0039211 CG13620 CG13620 SD03914
sensory nervous system specific anlage;	
	FBgn0040271 Sulf1 CG6725 LD03060
	FBgn0002932 neur CG11988 LD45505
	FBgn0004595 pros CG17228 GH11848
	FBgn0002561 l(1)sc CG3839 RE59335
	FBgn0017453 Zn72D CG5215 GH12756
	FBgn0035186 CG13912 CG13912 RE20371
	FBgn0013725 phyl CG10108 RH04401
ventral ectoderm anlage;	
	FBgn0036494 Toll-6 CG7250 LD08841
	FBgn0002932 neur CG11988 LD45505
	FBgn0004595 pros CG17228 GH11848
	FBgn0040230 dbo CG6224 RE13447
	FBgn0002561 l(1)sc CG3839 RE59335
	FBgn0037947 CG6930 CG6930 RE72719
	FBgn0037811 CG12592 CG12592 AT09724
	FBgn0003300 run CG1849 GH02614
	FBgn0015903 apt CG5393 LD45581
	FBgn0033992 CG11798 CG11798 LD23883
	FBgn0000611 exd CG8933 LD03509
	FBgn0000577 en CG9015 LD16125
	FBgn0003346 Sd CG9999 LD16356
	FBgn0038118 NULL CG7855 RE03551
	FBgn0035186 CG13912 CG13912 RE20371
	FBgn0010453 Wnt4 CG4698 RE26454
	FBgn0031971 CG7224 CG7224 GH18422
	FBgn0000577 en CG9015 LD16125
embryonic/larval visceral muscle;	
	FBgn0000611 exd CG8933 LD03509
	FBgn0015773 NetA CG18657 RE11206
	FBgn0004567 slp2 CG2939 RE11345
ventral midline glia;	
	FBgn0035542 CG11347 CG11347 GH28550
pars intercerebralis primordium;	
	FBgn0040271 Sulf1 CG6725 LD03060
	FBgn0040271 Sulf1 CG6725 SD04414
	FBgn0038118 NULL CG7855 RE03551
	continued on next page...

Table 18 continued from previous page.

Anatomical Structures	Genes
	FBgn0031971 CG7224 CG7224 GH18422
embryonic dorsal pouch;	
	FBgn0040230 dbo CG6224 RE13447
	FBgn0015903 apt CG5393 LD45581
	FBgn0039211 CG13620 CG13620 SD03914
embryonic head epidermis;	
	FBgn0004868 Gdi CG4422 LD46767
	FBgn0035542 CG11347 CG11347 GH28550
	FBgn0040230 dbo CG6224 RE13447
	FBgn0033992 CG11798 CG11798 LD23883
	FBgn0039211 CG13620 CG13620 SD03914
	FBgn0000577 en CG9015 LD16125
	FBgn0000659 fkh CG10002 RE03865
	FBgn0017453 Zn72D CG5215 GH12756
	FBgn0015773 NetA CG18657 RE11206
	FBgn0000577 en CG9015 LD16125
lymph gland;	
	FBgn0037811 CG12592 CG12592 AT09724
	FBgn0038118 NULL CG7855 RE03551
embryonic foregut;	
	FBgn0004868 Gdi CG4422 LD46767
	FBgn0015903 apt CG5393 LD45581
	FBgn0039211 CG13620 CG13620 SD03914
	FBgn0000659 fkh CG10002 RE03865
	FBgn0038150 yellow-e3 CG17045 RE14803
	FBgn0010453 Wnt4 CG4698 RE26454
embryonic central nervous system;	
	FBgn0040271 Sulf1 CG6725 LD03060
	FBgn0024332 Mcm3 CG4206 LD07144
	FBgn0036494 Toll-6 CG7250 LD08841
	FBgn0004868 Gdi CG4422 LD46767
	FBgn0002932 neur CG11988 LD45505
	FBgn0035542 CG11347 CG11347 GH28550
	FBgn0040271 Sulf1 CG6725 SD04414
	FBgn0004595 pros CG17228 GH11848
	FBgn0040230 dbo CG6224 RE13447
	FBgn0002561 l(1)sc CG3839 RE59335
	FBgn0037947 CG6930 CG6930 RE72719
	FBgn0037811 CG12592 CG12592 AT09724
	FBgn0005677 dac CG4952 RE64054
	FBgn0003300 run CG1849 GH02614
	FBgn0051670 CG31670 CG31670 GH14092
	FBgn0004584 Rrp1 CG3178 LP05366
	FBgn0015903 apt CG5393 LD45581
	FBgn0033992 CG11798 CG11798 LD23883
	FBgn0039211 CG13620 CG13620 SD03914

continued on next page...

Table 18 continued from previous page.

Anatomical Structures	Genes
	FBgn0000611 exd CG8933 LD03509
	FBgn0034878 CG3941 CG3941 LD15650
	FBgn0000577 en CG9015 LD16125
	FBgn0003346 Sd CG9999 LD16356
	FBgn0038118 NULL CG7855 RE03551
	FBgn0000659 fkh CG10002 RE03865
	FBgn0017453 Zn72D CG5215 GH12756
	FBgn0004567 slp2 CG2939 RE11345
	FBgn0036786 skl CG13701 RE14076
	FBgn0035186 CG13912 CG13912 RE20371
	FBgn0010453 Wnt4 CG4698 RE26454
	FBgn0029512 Aos1 CG12276 LD33652
	FBgn0031971 CG7224 CG7224 GH18422
	FBgn0051670 CG31670 CG31670 GH14092
	FBgn0034878 CG3941 CG3941 LD15650
	FBgn0000577 en CG9015 LD16125
embryonic main segment of Malpighian tubule;	FBgn0000659 fkh CG10002 RE03865
tracheal primordium;	FBgn0037811 CG12592 CG12592 AT09724
	FBgn0015903 apt CG5393 LD45581
	FBgn0039211 CG13620 CG13620 SD03914
procephalic neuroblasts;	FBgn0024332 Mcm3 CG4206 LD07144
	FBgn0013469 NULL CG12296 RE58581
	FBgn0040271 Sulf1 CG6725 SD04414
	FBgn0004595 pros CG17228 GH11848
	FBgn0040230 dbo CG6224 RE13447
	FBgn0002561 l(1)sc CG3839 RE59335
	FBgn0005677 dac CG4952 RE64054
	FBgn0033992 CG11798 CG11798 LD23883
	FBgn0034878 CG3941 CG3941 LD15650
	FBgn0035186 CG13912 CG13912 RE20371
	FBgn0010453 Wnt4 CG4698 RE26454
	FBgn0034878 CG3941 CG3941 LD15650
embryonic small intestine;	FBgn0040271 Sulf1 CG6725 LD03060
	FBgn0000659 fkh CG10002 RE03865
	FBgn0010453 Wnt4 CG4698 RE26454
rectum specific anlage;	FBgn0000659 fkh CG10002 RE03865
embryonic brain;	FBgn0024332 Mcm3 CG4206 LD07144
	FBgn0013469 NULL CG12296 RE58581
	FBgn0036494 Toll-6 CG7250 LD08841
	FBgn0004868 Gdi CG4422 LD46767

continued on next page...

Table 18 continued from previous page.	
Anatomical Structures	Genes
	FBgn0035542 CG11347 CG11347 GH28550<br>FBgn0040271 Sulf1 CG6725 SD04414<br>FBgn0040230 dbo CG6224 RE13447<br>FBgn0005677 dac CG4952 RE64054<br>FBgn0003300 run CG1849 GH02614<br>FBgn0015903 apt CG5393 LD45581<br>FBgn0033992 CG11798 CG11798 LD23883<br>FBgn0039211 CG13620 CG13620 SD03914<br>FBgn0000611 exd CG8933 LD03509<br>FBgn0034878 CG3941 CG3941 LD15650<br>FBgn0000577 en CG9015 LD16125<br>FBgn0038118 NULL CG7855 RE03551<br>FBgn0000659 fkh CG10002 RE03865<br>FBgn0017453 Zn72D CG5215 GH12756<br>FBgn0004567 slp2 CG2939 RE11345<br>FBgn0036786 skl CG13701 RE14076<br>FBgn0035186 CG13912 CG13912 RE20371<br>FBgn0010453 Wnt4 CG4698 RE26454<br>FBgn0029512 Aos1 CG12276 LD33652<br>FBgn0034878 CG3941 CG3941 LD15650<br>FBgn0000577 en CG9015 LD16125
trunk mesoderm anlage;	FBgn0024332 Mcm3 CG4206 LD07144<br>FBgn0002932 neur CG11988 LD45505<br>FBgn0037947 CG6930 CG6930 RE72719<br>FBgn0033992 CG11798 CG11798 LD23883<br>FBgn0000611 exd CG8933 LD03509<br>FBgn0000577 en CG9015 LD16125<br>FBgn0003346 Sd CG9999 LD16356<br>FBgn0015773 NetA CG18657 RE11206<br>FBgn0035186 CG13912 CG13912 RE20371<br>FBgn0000577 en CG9015 LD16125
anterior endoderm anlage;	FBgn0002932 neur CG11988 LD45505<br>FBgn0037947 CG6930 CG6930 RE72719<br>FBgn0015903 apt CG5393 LD45581<br>FBgn0000611 exd CG8933 LD03509<br>FBgn0003346 Sd CG9999 LD16356<br>FBgn0000659 fkh CG10002 RE03865<br>FBgn0035186 CG13912 CG13912 RE20371
embryonic Malpighian tubule;	FBgn0038118 NULL CG7855 RE03551<br>FBgn0000659 fkh CG10002 RE03865
ventral nerve cord primordium;	FBgn0040271 Sulf1 CG6725 LD03060<br>FBgn0024332 Mcm3 CG4206 LD07144

continued on next page...

Table 18 continued from previous page.

Anatomical Structures	Genes
	FBgn0013469 NULL CG12296 RE58581
	FBgn0036494 Toll-6 CG7250 LD08841
	FBgn0004868 Gdi CG4422 LD46767
	FBgn0002932 neur CG11988 LD45505
	FBgn0035542 CG11347 CG11347 GH28550
	FBgn0040271 Sulf1 CG6725 SD04414
	FBgn0004595 pros CG17228 GH11848
	FBgn0040230 dbo CG6224 RE13447
	FBgn0002561 l(1)sc CG3839 RE59335
	FBgn0037947 CG6930 CG6930 RE72719
	FBgn0037811 CG12592 CG12592 AT09724
	FBgn0003300 run CG1849 GH02614
	FBgn0004584 Rrp1 CG3178 LP05366
	FBgn0015903 apt CG5393 LD45581
	FBgn0033992 CG11798 CG11798 LD23883
	FBgn0000611 exd CG8933 LD03509
	FBgn0030507 CG11164 CG11164 RE50056
	FBgn0034878 CG3941 CG3941 LD15650
	FBgn0000577 en CG9015 LD16125
	FBgn0003346 Sd CG9999 LD16356
	FBgn0038118 NULL CG7855 RE03551
	FBgn0017453 Zn72D CG5215 GH12756
	FBgn0015773 NetA CG18657 RE11206
	FBgn0004567 slp2 CG2939 RE11345
	FBgn0035186 CG13912 CG13912 RE20371
	FBgn0010453 Wnt4 CG4698 RE26454
	FBgn0013725 phyl CG10108 RH04401
	FBgn0029512 Aos1 CG12276 LD33652
	FBgn0034878 CG3941 CG3941 LD15650
	FBgn0000577 en CG9015 LD16125
atrium;	
	FBgn0015903 apt CG5393 LD45581
	FBgn0000577 en CG9015 LD16125
	FBgn0000577 en CG9015 LD16125
adult midgut precursor;	
	FBgn0004595 pros CG17228 GH11848
lateral cord;	
	FBgn0040271 Sulf1 CG6725 LD03060
	FBgn0024332 Mcm3 CG4206 LD07144
	FBgn0036494 Toll-6 CG7250 LD08841
	FBgn0004868 Gdi CG4422 LD46767
	FBgn0002932 neur CG11988 LD45505
	FBgn0035542 CG11347 CG11347 GH28550
	FBgn0040271 Sulf1 CG6725 SD04414
	FBgn0004595 pros CG17228 GH11848
	FBgn0040230 dbo CG6224 RE13447

continued on next page...

Table 18 continued from previous page.

Anatomical Structures	Genes
	FBgn0002561 l(1)sc CG3839 RE59335
	FBgn0037947 CG6930 CG6930 RE72719
	FBgn0005677 dac CG4952 RE64054
	FBgn0003300 run CG1849 GH02614
	FBgn0004584 Rrp1 CG3178 LP05366
	FBgn0015903 apt CG5393 LD45581
	FBgn0033992 CG11798 CG11798 LD23883
	FBgn0000611 exd CG8933 LD03509
	FBgn0030507 CG11164 CG11164 RE50056
	FBgn0034878 CG3941 CG3941 LD15650
	FBgn0000577 en CG9015 LD16125
	FBgn0003346 Sd CG9999 LD16356
	FBgn0038118 NULL CG7855 RE03551
	FBgn0017453 Zn72D CG5215 GH12756
	FBgn0015773 NetA CG18657 RE11206
	FBgn0004567 slp2 CG2939 RE11345
	FBgn0036786 skl CG13701 RE14076
	FBgn0035186 CG13912 CG13912 RE20371
	FBgn0010453 Wnt4 CG4698 RE26454
	FBgn0029512 Aos1 CG12276 LD33652
	FBgn0034878 CG3941 CG3941 LD15650
	FBgn0000577 en CG9015 LD16125
posterior endoderm anlage;	
	FBgn0002932 neur CG11988 LD45505
	FBgn0002561 l(1)sc CG3839 RE59335
	FBgn0037947 CG6930 CG6930 RE72719
	FBgn0015903 apt CG5393 LD45581
	FBgn0000611 exd CG8933 LD03509
	FBgn0003346 Sd CG9999 LD16356
	FBgn0000659 fkh CG10002 RE03865
	FBgn0035186 CG13912 CG13912 RE20371
trunk mesoderm primordium;	
	FBgn0035542 CG11347 CG11347 GH28550
	FBgn0002561 l(1)sc CG3839 RE59335
	FBgn0033992 CG11798 CG11798 LD23883
	FBgn0000611 exd CG8933 LD03509
	FBgn0000577 en CG9015 LD16125
	FBgn0003346 Sd CG9999 LD16356
	FBgn0015773 NetA CG18657 RE11206
	FBgn0000577 en CG9015 LD16125
protocerebrum primordium;	
	FBgn0040271 Sulf1 CG6725 LD03060
	FBgn0024332 Mcm3 CG4206 LD07144
	FBgn0013469 NULL CG12296 RE58581
	FBgn0036494 Toll-6 CG7250 LD08841
	FBgn0004868 Gdi CG4422 LD46767

continued on next page...

Table 18 continued from previous page.

Anatomical Structures	Genes
	FBgn0002932 neur CG11988 LD45505
	FBgn0035542 CG11347 CG11347 GH28550
	FBgn0040271 Sulf1 CG6725 SD04414
	FBgn0004595 pros CG17228 GH11848
	FBgn0040230 dbo CG6224 RE13447
	FBgn0002561 l(1)sc CG3839 RE59335
	FBgn0037947 CG6930 CG6930 RE72719
	FBgn0037811 CG12592 CG12592 AT09724
	FBgn0005677 dac CG4952 RE64054
	FBgn0003300 run CG1849 GH02614
	FBgn0051670 CG31670 CG31670 GH14092
	FBgn0004584 Rrp1 CG3178 LP05366
	FBgn0015903 apt CG5393 LD45581
	FBgn0033992 CG11798 CG11798 LD23883
	FBgn0039211 CG13620 CG13620 SD03914
	FBgn0000611 exd CG8933 LD03509
	FBgn0030507 CG11164 CG11164 RE50056
	FBgn0034878 CG3941 CG3941 LD15650
	FBgn0000577 en CG9015 LD16125
	FBgn0003346 Sd CG9999 LD16356
	FBgn0038118 NULL CG7855 RE03551
	FBgn0000659 fkh CG10002 RE03865
	FBgn0017453 Zn72D CG5215 GH12756
	FBgn0015773 NetA CG18657 RE11206
	FBgn0004567 slp2 CG2939 RE11345
	FBgn0036786 skl CG13701 RE14076
	FBgn0038150 yellow-e3 CG17045 RE14803
	FBgn0035186 CG13912 CG13912 RE20371
	FBgn0010453 Wnt4 CG4698 RE26454
	FBgn0013725 phyl CG10108 RH04401
	FBgn0029512 Aos1 CG12276 LD33652
	FBgn0031971 CG7224 CG7224 GH18422
	FBgn0051670 CG31670 CG31670 GH14092
	FBgn0034878 CG3941 CG3941 LD15650
	FBgn0000577 en CG9015 LD16125
embryonic/larval garland cell;	FBgn0004595 pros CG17228 GH11848
embryonic dorsal pouch primordium;	FBgn0015903 apt CG5393 LD45581
embryonic/larval tracheal system;	FBgn0039211 CG13620 CG13620 SD03914
ventral epidermis primordium;	FBgn0036494 Toll-6 CG7250 LD08841
	FBgn0035542 CG11347 CG11347 GH28550
	FBgn0040230 dbo CG6224 RE13447
	FBgn0037947 CG6930 CG6930 RE72719

continued on next page...

Table 18 continued from previous page.

Anatomical Structures	Genes
	FBgn0037811 CG12592 CG12592 AT09724<br>FBgn0003300 run CG1849 GH02614<br>FBgn0015903 apt CG5393 LD45581<br>FBgn0033992 CG11798 CG11798 LD23883<br>FBgn0000611 exd CG8933 LD03509<br>FBgn0000577 en CG9015 LD16125<br>FBgn0004567 slp2 CG2939 RE11345<br>FBgn0038150 yellow-e3 CG17045 RE14803<br>FBgn0035186 CG13912 CG13912 RE20371<br>FBgn0010453 Wnt4 CG4698 RE26454<br>FBgn0000577 en CG9015 LD16125
neuroblasts of ventral nervous system;	FBgn0024332 Mcm3 CG4206 LD07144<br>FBgn0036494 Toll-6 CG7250 LD08841<br>FBgn0002932 neur CG11988 LD45505<br>FBgn0040271 Sulf1 CG6725 SD04414<br>FBgn0004595 pros CG17228 GH11848<br>FBgn0040230 dbo CG6224 RE13447<br>FBgn0002561 l(1)sc CG3839 RE59335<br>FBgn0003300 run CG1849 GH02614<br>FBgn0033992 CG11798 CG11798 LD23883<br>FBgn0030507 CG11164 CG11164 RE50056<br>FBgn0034878 CG3941 CG3941 LD15650<br>FBgn0003346 Sd CG9999 LD16356<br>FBgn0038118 NULL CG7855 RE03551<br>FBgn0035186 CG13912 CG13912 RE20371<br>FBgn0034878 CG3941 CG3941 LD15650
embryonic antennal sense organ;	FBgn0004868 Gdi CG4422 LD46767
hindgut anlage;	FBgn0040230 dbo CG6224 RE13447<br>FBgn0003300 run CG1849 GH02614<br>FBgn0015903 apt CG5393 LD45581<br>FBgn0000611 exd CG8933 LD03509<br>FBgn0000577 en CG9015 LD16125<br>FBgn0003346 Sd CG9999 LD16356<br>FBgn0000659 fkh CG10002 RE03865<br>FBgn0031971 CG7224 CG7224 GH18422<br>FBgn0000577 en CG9015 LD16125
